# Comparative Effectiveness of Interventions to Treat Cancer Treatment-Related Cognitive Impairment in Adult Cancer Survivors Following Systemic Therapy: A Systematic Review with Network Meta-Analyses

**DOI:** 10.3390/cancers17213430

**Published:** 2025-10-26

**Authors:** Dianna M. Wolfe, Candyce Hamel, Jason Berard, Areti Angeliki Veroniki, Becky Skidmore, Salmaan Kanji, Kiran Rabheru, Sharon F. McGee, Leta Forbes, Igor de Lima Machado, Michelle Liu, Deanna Saunders, Lisa Vandermeer, Mark Clemons, Brian Hutton

**Affiliations:** 1 Methodological and Implementation Research Program, Ottawa Hospital Research Institute, The Ottawa Hospital, Ottawa, ON K1H 8L6, Canada; dwolfe@ohri.ca (D.M.W.); cahamel@ohri.ca (C.H.); skanji@ohri.ca (S.K.); 2 Neuroscience Program, Ottawa Hospital Research Institute, The Ottawa Hospital, Ottawa, ON K1H 8L6, Canada; jberard@ohri.ca; 3 Brain and Mind Research Institute, University of Ottawa, Ottawa, ON K1H 8M5, Canada; 4 Center for Evidence Synthesis in Health, Department of Health Services, Policy, and Practice, School of Public Health, Brown University, Providence, RI 02912, USA; argie_veroniki@brown.edu; 5 Knowledge Translation Program, Li Ka Shing Knowledge Institute, St. Michael’s Hospital, Toronto, ON M5B 1W8, Canada; 6 Institute of Health Policy, Management and Evaluation, University of Toronto, Toronto, ON M5T 3M6, Canada; 7 Independent Researcher, Ottawa, ON K1H 8L6, Canada; becky.skidmore.rls@gmail.com; 8 Department of Pharmacy, The Ottawa Hospital, Ottawa, ON K1H 8L6, Canada; 9 Department of Psychiatry, University of Ottawa, Ottawa, ON K1H 8L6, Canada; krabheru@toh.ca; 10 Division of Medical Oncology, The Ottawa Hospital, Ottawa, ON K1H 8L6, Canada; shmcgee@toh.ca (S.F.M.); mclemons@toh.ca (M.C.); 11 Department of Medicine, University of Ottawa, Ottawa, ON K1H 8M5, Canada; 12 Ontario Health (Cancer Care Ontario), Toronto, ON M5G 2L3, Canada; leta.forbes@ontariohealth.ca; 13 Division of Medical Oncology, Durham Regional Cancer Centre, Lakeridge Health, Oshawa, ON L1G 2B9, Canada; 14 Cancer Research Program, Ottawa Hospital Research Institute, The Ottawa Hospital, Ottawa, ON K1H 8L6, Canadadsaunders@ohri.ca (D.S.); lvandermeer@ohri.ca (L.V.); 15 School of Epidemiology and Public Health, University of Ottawa, 600 Peter Morand Crescent, Ottawa, ON K1G 5Z3, Canada

**Keywords:** cancer-treatment-related cognitive impairment, network meta-analysis, cognitive rehabilitation, cancer survivorship, systematic review

## Abstract

**Simple Summary:**

Many cancer survivors experience lasting problems with memory, attention, and other aspects of cognitive functioning after chemotherapy or other systemic treatments—often referred to as cancer treatment-related cognitive impairment (CTRCI). To better understand which treatments may help, we reviewed evidence from randomized controlled trials testing psychological, pharmacological, and other interventions for adults with established CTRCI. Across 18 studies, a structured, therapist-led group program that combined patient education with cognitive rehabilitation showed the most consistent benefits, improving several areas of thinking and memory, while mindfulness-based interventions also showed some positive effects. In contrast, medications such as donepezil did not show benefit. Although the evidence remains limited and of low certainty, these findings suggest that group-based rehabilitation programs may offer the most promise for improving cognitive function in cancer survivors.

**Abstract:**

Background. Cancer treatment-related cognitive impairment (CTRCI) is a frequent and persistent consequence of systemic cancer therapy, adversely affecting quality of life and independence among cancer survivors. Methods. To clarify the relative effectiveness of available treatments, we conducted a systematic review and network meta-analyses of randomized controlled trials evaluating psychological, pharmacological, and other interventions for established CTRCI in adult survivors of non-central nervous system cancers. Eligible trials reported objective outcomes in one or more of eight cognitive domains, including learning, memory, processing speed, word generation, cognitive flexibility, attention, working memory, and abstraction. Results. Eighteen studies met inclusion criteria, with 14 trials (*n* = 1100) contributing to network meta-analyses of immediate post-intervention effects across seven domains. A therapist-guided group intervention combining patient education and cognitive rehabilitation consistently ranked highest and was associated with significantly improved learning, memory, processing speed, attention, and working memory compared with a waitlist control, although the certainty of evidence (CoE) was low to very low and largely based on a single trial. Mindfulness-based interventions were also associated with improved processing speed (low CoE). Donepezil was associated with no benefit versus placebo for any domain. Conclusions. While findings suggest that structured multimodal group interventions may represent the most promising strategy for CTRCI, CoE was low, and additional rigorous, standardized trials are required.

## 1. Introduction

Cancer-related cognitive impairment (CRCI) is a common and potentially debilitating consequence of cancer and its treatment. CRCI has well-documented adverse effects on quality of life, social participation, and daily functioning, often persisting long after treatment completion [1]. Multiple factors contribute to its development, including the direct effects of the cancer itself (e.g., tumour-derived inflammatory mediators) [2,3]; systemic therapies, such as chemotherapy and immunotherapy [2,4,5]; concurrent treatments [2,6]; and comorbid conditions such as anxiety, depression, fatigue, stress, and sleep disturbances [4,7,8]. Patient-level characteristics—including age, genetic factors, educational attainment, and racio-ethnic background—also influence susceptibility to CRCI [2,7,9,10].

Among the contributors to CRCI, systemic cancer treatments play a particularly prominent role. Cytotoxic agents can damage the central nervous system by causing neuroinflammation, neurotransmitter dysregulation, impaired neurogenesis, and disruption of the blood–brain barrier, leading to what is often described as cancer-treatment-related cognitive impairment (CTRCI) [6,9]. Patients receiving systemic therapy are significantly more likely to experience cognitive deficits than those who do not [11], with the type, intensity, and cumulative dose of chemotherapy further influencing the severity of impairment [2,5,9,12]. Pooled prevalence estimates indicate that between 21% and 35% of survivors exhibit objectively measured CTRCI at one year post-treatment across cancer types [11,13,14,15,16], with minimal improvement over time [9,13,16,17]. These findings highlight the widespread, persistent impact of systemic cancer therapy on cognitive function.

The International Cognition and Cancer Task Force (ICCTF) has identified learning and memory, processing speed, and executive functions as the core cognitive domains most affected in CTRCI and has issued recommendations for appropriate neuropsychological assessments to evaluate deficits in these areas [18]. Despite this guidance, primary studies investigating interventions for CRCI and CTRCI are highly heterogeneous—with substantial variation in the cognitive domains targeted, assessment tools employed, study designs, timing of follow-up, patient populations, intervention types, and risk of bias [19]. Existing systematic reviews have typically either performed meta-analyses despite this clinical and methodological heterogeneity [20,21,22,23,24,25,26,27,28] or have been limited to descriptive summaries without quantitative synthesis [19,29,30,31,32,33].

To date, no systematic review has rigorously synthesized the evidence on interventions for CTRCI using methods designed to address clinical and methodological heterogeneity. We conducted a systematic review and network meta-analysis (NMA) to evaluate the relative effects of treatments for persistent CTRCI in adult survivors of non-central nervous system cancers who had received systemic therapy. Our analysis focused on objective cognitive outcomes, applying stringent inclusion criteria and analytic methods to minimize heterogeneity and enhance the validity of our findings.

### Review Question

What are the relative benefits associated with psychological, pharmacological, and other interventions for cancer-treatment-related cognitive impairment (hereafter referred to as ‘CTRCI’) in adult non-central nervous system (CNS) cancer survivors post systemic therapy?

## 2. Methods

### 2.1. Protocol, Registration, and Reporting

The review process was guided by the established systematic review methodology of the Cochrane Collaboration [34]. A review protocol was developed and published a priori [35], and registered on the Open Science Framework (OSF; https://osf.io/ qvbs8; accessed on 13 October 2023). We describe minor protocol amendments that occurred during the review process in File S1. Reporting within this manuscript is in accordance with the Preferred Reporting Items for Systematic Reviews and Meta-analysis (PRISMA) 2020 statement [36] and the PRISMA Extension for NMA [37].

### 2.2. Patient and Public Involvement

Multiple organizations representing people with lived experience were consulted during the review process, including Ontario Health, the CURE Foundation, and the Canadian Association of Nursing Oncology/Association Canadienne des Infirmières en Oncologie.

### 2.3. Study Eligibility Criteria

The Population–Intervention–Comparator–Outcome–Study design (PICOS) criteria defining study eligibility have been summarized previously in our published protocol [35] and in File S2. Briefly, randomized controlled trials (RCTs; both parallel-group and cross-over) evaluating any intervention for the treatment of existing CTRCI in adult (≥18 years) cancer survivors who had previously completed systemic treatment were included. Studies of patients actively receiving systemic therapy other than long-term hormonal therapy were excluded because the focus of such studies would be on CTRCI prevention rather than treatment. We excluded studies of survivors of CNS tumours or metastases to reduce clinical heterogeneity related to the direct effects of CNS tumours on cognitive impairment. Studies that selected participants based on a CTRCI-associated condition (e.g., fatigue, insomnia, depression, stress, anxiety) or that included a mix of patients with and without CTRCI were included if they reported relevant cognitive function subgroup analyses for patients with CTRCI at baseline.

Interventions of interest included any psychological (e.g., cognitive rehabilitation/training, cognitive behavioural therapy), pharmacological (e.g., donepezil, methylphenidate), and other therapies (e.g., mindfulness-based interventions (MBI), music therapy, exercise) compared to a waitlist/usual care control, placebo, or other active intervention. To be eligible, studies must have reported quantitative data in sufficient detail for at least one of thirteen objective cognitive function domains or subdomains of interest: verbal learning, verbal memory, visual learning, visual memory, processing speed, complex psychomotor speed, executive function (conceptual knowledge, mental flexibility/task switching, abstraction, inhibition, fluency), attention, or working memory. Only studies published as full texts in either English or French were included.

### 2.4. Data Sources and Search Strategy

An experienced medical information specialist developed and tested the search strategies through an iterative process in consultation with the review team. Another senior information specialist peer reviewed the MEDLINE strategy prior to execution using the PRESS Checklist [38].

Using the multifile option and deduplication tool available on the Ovid platform, we searched Ovid MEDLINE^®^ ALL, Embase Classic + Embase, APA PsycInfo, and the Cochrane Central Register of Controlled Trials (CENTRAL). We also searched CINAHL on Ebsco. We applied a combination of controlled vocabulary (e.g., “Chemotherapy-Related Cognitive Impairment”, “Antineoplastic Agents”, “Cognitive Dysfunction”) and keywords (e.g., “chemo fog”, “systematic treatment”, “mental recall”), adjusting the vocabulary and syntax as necessary across the databases. We applied an amended version of the 2008 Cochrane highly sensitive search strategy (sensitivity- and precision-maximizing version) in all databases but CENTRAL, which is prefiltered for randomized and controlled clinical trials. We did not restrict by language but did limit results to the publication year 1980 to the present, and where applicable, removed animal-only and opinion pieces. We performed all searches on 6 August 2023 and updated them on 15 October 2024. A search of the grey literature was undertaken for approximately 35 reviewer-hours between 29 March and 8 April 2025 [39]. Additionally, the reference lists of systematic reviews identified in the topic area were hand searched for potentially relevant RCTs. Specific details regarding the search strategies and detailed grey literature search methods are provided in File S3.

### 2.5. Study Selection Process

Citations identified through the literature searches were amalgamated and de-duplicated in reference management software (EndNote Version 9.3.3 [40]) prior to being uploaded to an online systematic review platform (DistillerSR^®^ Version 2025.1.2, Evidence Partners, Ottawa, ON, Canada; 2025. https://www.evidencepartners.com). Screening of titles and abstracts (Level 1) and full texts (Level 2) was conducted by two independent reviewers (amongst DW, CH, BH, LV, ML, DS) and operationalized in DistillerSR^®^ using forms piloted in batches of 50 citations (Level 1) and 5 full texts (Level 2) to ensure consistent application of eligibility criteria across reviewers. Screening questions were adjusted as necessary, and the piloting process continued until reviewer agreement reached approximately 95%. During the screening process, conflicts were resolved through discussion or input from a third reviewer (BH).

Given the high yield of the search process (>17,000 citations), study selection at Level 1 was supported by artificial intelligence/machine learning (AI/ML) tools within the DistillerSR^®^ platform [41]. The AI/ML tool was used to inform prioritized screening (i.e., it found the most likely relevant studies first and promoted them for review) and to serve as a secondary screener once metrics suggested that most or all of the relevant studies had been fully screened. Details regarding the AI workflow, including training and stopping parameters, are provided in File S2.

### 2.6. Data Extraction

Data extraction was performed by a single reviewer into standardized forms in Excel^®^ for Microsoft 365 MOS (Microsoft Corporation, Redmond, WA, USA), with verification by a second reviewer. Conflicts were resolved through discussion or consultation with a third reviewer. The following data were sought: study characteristics (e.g., year of publication, country, funding source, objective), patient demographics (e.g., age, sex, cancer type, education level, baseline measures of cognitive function, fatigue, depression), intervention and comparator characteristics (e.g., description, dose, frequency, duration, setting), and outcome data (e.g., assessment tool used, follow-up time, group level data at baseline and follow-up, including sample size, mean score, standard deviation).

#### Cognitive Function Assessment Tools of Interest

Previous reviews of cognitive function across a variety of medical conditions have demonstrated the challenge of classifying the vast number of neuropsychological tests reported in the literature into cognitive domains of interest [42,43,44,45,46]. To streamline data extraction, we identified assessment tools of interest to our review a priori. We initially mapped each neuropsychological test reported in each included study to obtain an understanding of the diversity of tools. Recognizing that individual neuropsychological tests tend to assess multiple cognitive domains and subdomains, based on a previously accepted approach [43], we categorized each test according to the cognitive domain that it was decided to assess best, utilizing a combination of content expertise and by referencing publisher test manuals, test compendiums [47], and gold-standard reference texts on neuropsychological assessment procedures [48]. We focused our scales of interest to (1) those recommended by the ICCTF [18] [i.e., Hopkins Verbal Learning Test-Revised (HVLT-R), the Trail Making Test (TMT) parts A and B, and the Controlled Oral Word Association test (COWAT)], (2) other scales that measured one of the 13 cognitive domains of interest and that were identified by our clinical content experts to have been validated and/or in common use in clinical research, and (3) scales with readily available normative data and clear documentation within the study (e.g., which subscales were used). Neuropsychological tests administered via online platforms were excluded from synthesis unless the specific subtests were explicitly identified, commonly used in the literature, and supported by readily available normative data. The final selection of tests (File S2) was used to guide data extraction.

### 2.7. Risk of Bias and Certainty of Evidence Assessments

Assessment of risk of bias (ROB) for objective cognitive function outcomes was conducted using the Cochrane Risk of Bias tool (version 1) [49], with verification by a second reviewer. Conflicts were resolved through discussion. A high ROB due to lack of blinding was assessed for studies where blinding was not possible (i.e., for many NPIs). A high ROB due to incomplete outcome data was assessed if data from ≥15% of participants were not available at follow-up and the missing data were not balanced between groups. Bias due to selective outcome reporting was assessed by comparing outcomes reported in the study protocol to those reported in the final published manuscript. Baseline imbalances between groups in key confounders (e.g., depression, education level, use of psychotropic medication) were assessed as other sources of potential bias. Given our interest in whether data used in our syntheses could bias our results, we assessed ROB of the raw data that were extracted as opposed to the bias of the results reported in the study. Therefore, where adjusted findings were reported that corrected for baseline imbalances between groups, we assigned a high ROB even though the adjusted findings reported within the study may have low ROB. An overall judgement of ROB for objective cognitive function outcomes was determined by the highest domain-level ROB assessment.

For the primary analyses of each outcome, certainty of evidence was assessed with the online, automated Confidence in Network Meta-Analysis (CINeMA) framework [50,51] (https://cinema.ispm.unibe.ch/# (accessed on 22 October 2025)) as *high*, *moderate*, *low*, or *very low*. We assessed imprecision, heterogeneity, and incoherence at a clinically important standardized mean difference (SMD) of 0.50. For all treatment comparisons, we assessed there to be ‘Some concerns’ for both reporting bias (i.e., publication bias) and indirectness. The mapping of study-level RoB judgments to CINeMA’s within-study bias domain followed the standard CINeMA algorithm, using the “Average” risk option to weight evidence contributions across comparisons. Comparisons with >50% contribution from high-risk studies were rated as “major concerns,” 25–50% as “some concerns,” and <25% as “no concerns” (see File S4 for detailed procedures). Other detailed methods for the process of CINeMA assessment, including the framework from which final confidence ratings were derived, can be found in File S4.

### 2.8. Data Synthesis and Statistical Methods

#### 2.8.1. Network Meta-Analysis Feasibility Assessment

Data cleaning and preliminary exploration of study characteristics and reported neuropsychiatric assessment tools were conducted in Excel^®^ for Microsoft 365 MOS (Microsoft Corporation). To assess the feasibility of NMA, we first defined outcomes of interest within the 14 cognitive domains and subdomains identified for data extraction, followed by classification/coding of interventions and network geometry, and explorations of between-study heterogeneity.

***Outcomes of interest:*** While a core outcome set for CTRCI trials has not been published, the International Cognition and Cancer Task Force (ICCTF) recommends the following cognitive domains be assessed, based upon the frontal subcortical profile of the effects of chemotherapy: learning and memory; processing speed; executive function, specifically complex attention; and working memory [18]. Previous reviews of cognitive function across other medical conditions [42,43,44,45,46] have adopted an approach proposed by Lezak et al. (2004) [48] that defined numerous categories of cognitive function, including executive function (abstraction, cognitive flexibility, inhibition, planning), verbal memory (immediate and delayed), visual memory (immediate and delayed), verbal fluency/skills, attention, processing speed, working memory, complex psychomotor ability, visuospatial ability/skills, intelligence, and motor speed. We used a hybrid approach to selection of objective cognitive function outcomes by identifying ICCTF-recommended cognitive domains within those proposed by Lezak et al. [48]. While this exercise was mainly clinically based, considerations were also made regarding data availability that could drive concerns of network structure and sparseness. In collaboration with our clinical experts (JB, KR), where possible, we grouped or split cognitive domains to achieve relative homogeneity of assessment tools and their measured cognitive constructs within each outcome, while aiming to retain sufficient data for quantitative analysis. For example, the verbal learning and visual learning domains were grouped to create a ‘learning’ outcome, while the executive function domain was split into “word generation,” “cognitive flexibility” (made up of mental flexibility/task switching and response inhibition/interference), and “abstraction” outcomes.

***Between-study heterogeneity and network transitivity:*** For each outcome of interest, we used evidence tables and box plots to subjectively explore between-study heterogeneity and transitivity of key effect modifiers, including study populations (i.e., age, sex, education level, proportion with breast cancer, months since completion of chemotherapy, and baseline measures of cognitive impairment, depression, anxiety, and fatigue) and study characteristics (i.e., treatment duration, assessment tool(s), follow-up time, ROB). Where sufficient data were reported, key effect modifiers were also assessed semi-objectively using recently published methodology [52] that calculates and visually portrays dissimilarities between treatment comparisons in study-level participant and methodological characteristics.

***Intervention classification/coding and network geometry***: Pharmacological interventions were classified according to drug name, dose, frequency, and duration of treatment. In collaboration with our content experts, we classified non-pharmacological interventions (NPIs) based on the details reported to allow us to identify the major intervention component that was being assessed (e.g., education, cognitive training/rehabilitation) and the context of the intervention (i.e., therapist-guided vs. self-guided, group vs. individual). We provide a detailed rationale for our approach and the considerations and frameworks underlying the formation of intervention groups for the network in File S2 [53,54,55,56,57]. The following characteristics were used to consider whether control group interventions should be considered one or multiple treatment nodes in the network: notification of being waitlisted vs. no waitlist (i.e., care as usual), contact with study researchers, provision of support from clinicians, provision of behavioural/educational guidance, and provision of placebo. Lumping and splitting of active and control interventions was conducted based on clinical relevance as well as consideration of geometry of the evidence network across outcomes. In consultation with clinical experts, placebo-controlled trials of pharmacologic therapies were synthesized separately from waitlist-controlled trials of NPIs, due to concerns that differences in the nature of the control conditions may lead to differential control-group responses. When the number of interventions was larger than the number of included studies in a network or the transitivity assumption was not appropriate for NMA, we deemed NMA to be infeasible and instead conducted pairwise meta-analyses (PWMAs) or descriptive syntheses.

#### 2.8.2. Data Optimization Strategies

Given the variability of assessment tools used within and across studies as well as the mixture of reporting formats for outcome data (e.g., group-level final follow-up values and mean changes from baseline), we employed several methods to optimize the amount of data available for quantitative analyses. Where multiple assessment tools were used within a study to measure the same cognitive domain (i.e., outcome), for example, learning measured with the HVLT-R total recall and the Brief Visuospatial Memory Test (BVMT) total recall, to maximize data inclusion and avoid statistical dependencies, we used a reductionist approach [58] and aggregated outcome data using established methods [59,60] (File S2). Where multiple assessment tools were used across studies to measure the same cognitive domain, pooled effect estimates were estimated as standardized mean differences (SMDs) in PWMAs and NMAs, where small, moderate, and large effects were represented by SMD thresholds of 0.20, 0.50, and 0.80, respectively [61]. Finally, we pooled group-level change scores (i.e., change from baseline) with mean scores at follow-up in PWMAs and NMAs, and conducted sensitivity analyses to determine the impact of including change scores on SMD effect estimates [34,62,63,64].

#### 2.8.3. Direct Intervention Comparisons

We conducted random-effects PWMAs for each head-to-head comparison reported for each outcome of interest. Between-study variance (tau-squared; τ^2^) was estimated using restricted maximum likelihood estimation (REML) methods and the Q-profile approach [65], with statistical heterogeneity estimated using I^2^ (i.e., the proportion of observed variability in effect estimates that could be explained by variability in the true effects of the included studies) [34,66]. We considered heterogeneity to be substantial when τ^2^ was greater than 50% and the *p*-value of the Q-statistic was less than 0.10 [34]. When I^2^ was greater than zero and three or more studies were included in the meta-analysis, the Knapp–Hartung–Sidik–Jonkman method was used to estimate the 95% confidence intervals (CIs) of the effect estimates [67].

#### 2.8.4. Approach to NMA

Where feasible, we conducted random-effects NMAs in the frequentist framework using the *netmeta* package in R (version 4.3.3), assuming a common between-study variance [68,69,70,71]. After consultation with our clinical experts, we focused our analyses on short-term intervention effects captured by data reported immediately post-intervention (e.g., after the final CBT session of a series of eight weekly sessions), and the durability of intervention effects captured by data reported at the longest follow-up time, reported as secondary analyses. Study contrasts that were identified as suggestive of contributing to intransitivity and/or inconsistency were flagged and removed in sensitivity analyses. Sensitivity analyses with change scores removed were also conducted as described above. If clinically meaningful changes in effects or reductions in heterogeneity were found in sensitivity analyses, careful consideration was given to any sources of clinical or methodological heterogeneity contributed by these study-contrasts to determine whether they should be retained in final analyses.

For the NMAs presented, we tested the consistency assumption globally using the design-by-treatment interaction test [72] and locally using the loop-specific methods [73]. Between-study variance (τ^2^) was estimated using REML [65] and compared against the empirically derived predictive distribution for nonpharmacological interventions targeting mental health outcomes, as reported by Rhodes et al. (2015; i.e., 95% range: 0.001–2.58) [74]. Relative treatment effects were reported as SMDs, with 95% CIs and PIs, and P-scores were estimated as secondary measures of effect. The findings of all final NMA models were summarized in league tables, as well as forest plots of treatment effects relative to that of a waitlist control. A rank heat plot demonstrated treatment ranks across all outcomes [75]. Further explorations of the impacts of key effect modifiers [35] by meta-regression or subgroup analysis could not be undertaken due to insufficient data. Due to the sparsity of data across networks, we were unable to conduct investigations of publication bias.

## 3. Results

**Figure 1:**
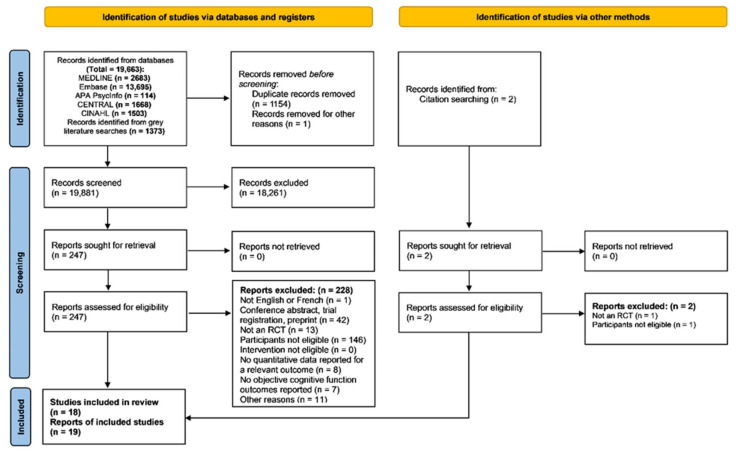
PRISMA flow diagram outlining the study selection process.

Of 19,883 abstract screened, 249 full texts were retrieved, and 19 reports of 18 studies were ultimately included in the review (Figure 1) [76,77,78,79,80,81,82,83,84,85,86,87,88,89,90,91,92,93,94]. Articles excluded during full-text screening are reported in File S5, with reasons for exclusion. Four were excluded from NMAs of immediate post-intervention data due to reporting of only long-term follow-up data [90] or being disconnected from the primary evidence network (i.e., control groups of a pharmacological therapy [87,91] and a NPI [94] were substantially different from waitlist), leaving 14 studies (*n* = 1100 patients) for inclusion in our primary NMAs [76,77,78,79,80,81,82,83,84,86,88,89,92,93].

The diagram details the number of records identified, screened, assessed for eligibility, and included in the review, along with reasons for exclusion of full-text reports. “Other reasons” for exclusion of full-text reports included the use of non-relevant outcome measures (e.g., platform-based scores or composite scores spanning multiple cognitive domains) and insufficient reporting of statistical methods, which precluded interpretation of outcome data.

### 3.1. Study Characteristics

The 14 studies included in NMAs were published between 2012 and 2024. Three studies were designed as pilot/feasibility trials [76,84,92], and none were associated with industry funding. The study protocol or registration was unavailable for five studies [77,78,82,83,92], and details regarding conflicts of interest were reported by all but one study [82].

Key features of the study populations are presented in Table 1, with additional information available in the Supporting Files of extracted data; details of our explorations of transitivity are presented in File S6. Overall, studies enrolled patients whose mean age ranged from 45.5 [92] to 59.2 [78] years and who had completed chemotherapy on average 11.2 [76] to 58.1 [77] months prior to randomization. All studies were conducted entirely or partially [77,78,80,93] in patients with breast cancer, as is also reflected by the proportions of female participants (median 100%, range 83% to 100%). In most studies, the average baseline cognitive function of participants was observed to be severely impaired (*n* = 11) [76,77,78,79,80,83,84,88,89,92,93], while baseline cognitive function was moderately impaired in one study [86] and mildly impaired in two studies [81,82]. Where reported, baseline depression and anxiety were generally absent (*n* = 7 [76,79,81,83,84,86,93] of 12 and 7 [76,77,78,79,83,84,93] of 10 studies reporting, respectively); the highest baseline depression and anxiety scores were moderate (*n* = 1) [92] and severe (*n* = 1) [88], respectively. Fatigue was more prevalent at baseline.

**Figure 2:**
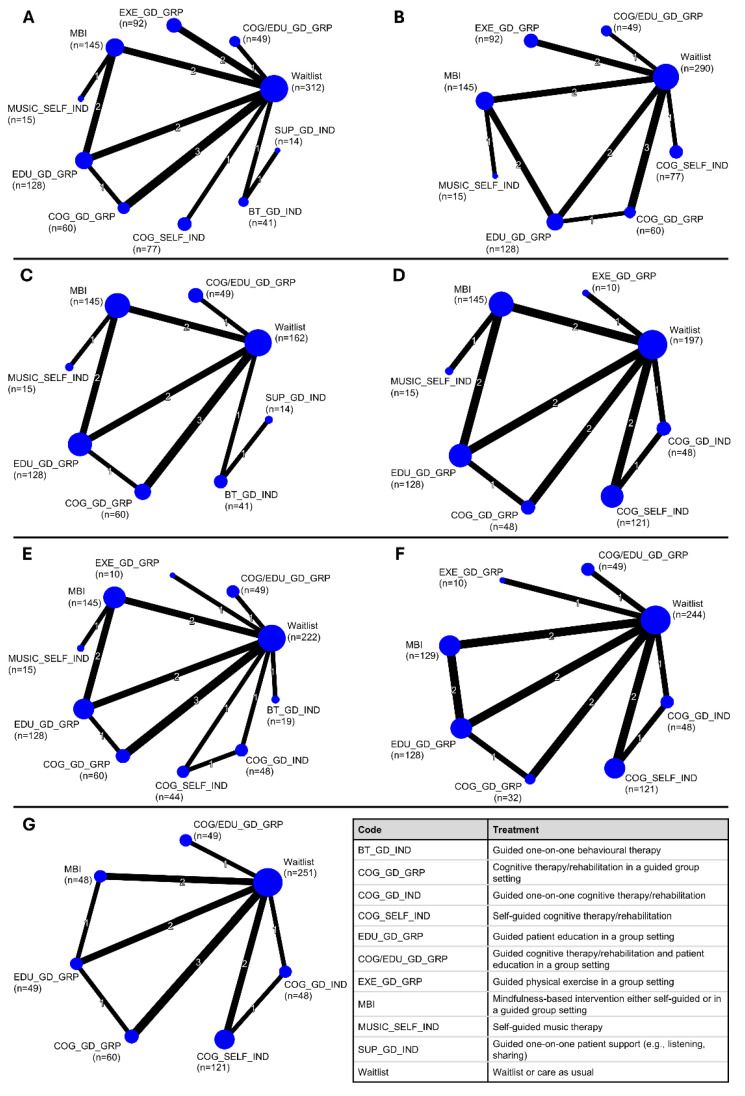
Evidence networks underlying network meta-analyses for each cognitive domain evaluated at immediate post-intervention. (**A**) Learning, (**B**), Memory, (**C**) Processing Speed, (**D**) Word Generation, (**E**) Cognitive Flexibility, (**F**) Attention, (**G**) Working Memory. Node size reflects the total number of participants randomized to each intervention, and edge (connecting line) thickness represents the number of trials informing each pair-wise comparison.

Ultimately, we identified eight outcomes of interest with data reported in at least one study: learning, memory, processing speed, word generation, cognitive flexibility, attention, working memory, and abstraction. Within those outcomes, the 14 studies informing NMAs compared waitlist control and ten active interventions: guided one-on-one behavioural therapy (from here forward referred to as BT_GD_IND), guided cognitive training/rehabilitation in a group setting (COG_GD_GRP), guided one-on-one cognitive training/rehabilitation (COG_GD_IND), self-guided cognitive training/rehabilitation (COG_SELF_IND), guided patient education in a group setting (EDU_GD_GRP), guided cognitive training/rehabilitation and patient education in a group setting (COG/EDU_GD_GRP), guided physical exercise in a group setting (EXE_GD_GRP), mindfulness-based interventions either self-guided or in a guided group setting (MBI), self-guided music therapy (MUSIC_SELF_IND), and guided one-on-one patient support (SUP_GD_IND). Detailed descriptions of intervention implementation are provided within the detailed data collected during the review. Across outcomes, networks were generally star-shaped around waitlist, with many single-study connections and nodes of small sample sizes (Figure 2). The average intervention duration for exercise studies [76,86] was 25 weeks and 7.4 weeks for non-exercise NPIs.

The assessment tools used to measure the cognitive domains of interest were highly variable (File S2). Regarding how missing outcome data were handled, eight studies conducted complete case analyses [76,77,78,80,81,83,86,92], three studies used linear mixed models that allowed missing data [79,88,89], and the remaining studies used either a last-observation-carried-forward [84] or linear interpolation approach [82] to missing data, or reported only raw data without analysis [93].

Data availability was greater for immediate post-intervention outcome measures than for those measures at long-term follow-up.

**Figure 3:**
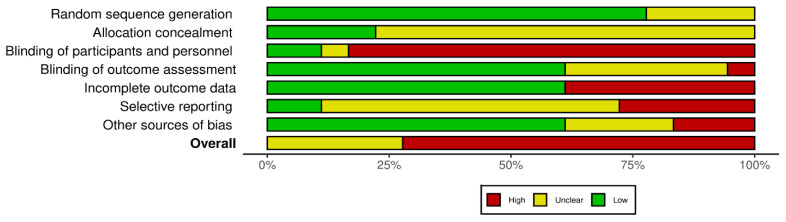
Summary of study-level risk of bias assessments using the Cochrane Risk of Bias tool (ROB-1). Proportions of included studies rated at low (green), unclear (yellow), and high (red) risk of bias are shown across seven domains: random sequence generation, allocation concealment, blinding (of participants/personnel and outcome assessors), incomplete outcome data, selective reporting, and other biases. An overall risk of bias summary is also provided.

Most studies were appraised to be at high ROB for objective cognitive function outcomes, with lack of blinding of participants and personnel and incomplete outcome data being the main sources of bias (Figure 3). Study-level ROB appraisals are provided in File S7.

### 3.2. Results from NMAs at Immediate Post-Intervention

Network meta-analyses were conducted on seven cognitive domains: learning, memory, processing speed, word generation, cognitive flexibility, attention, and working memory; supporting statistical information from NMAs are provided in File S8, while findings are described below. Only a single study reported data for abstraction, which has been summarized descriptively below.

Global and local tests demonstrated no evidence of inconsistency within NMAs (File S8), though power was limited. Subjective assessments of transitivity suggested that the COG/EDU_GD_GRP vs. waitlist comparison (*n* = 1 study [78]) differed substantially from other comparisons with respect to population demographics (older, highly educated male and female survivors of various cancers with long-standing, severe baseline cognitive impairment and fatigue). Semi-objective transitivity explorations suggested that within-comparison intransitivity (i.e., where more than one study provided direct evidence for a comparison, the studies differed in key effect modifiers) was present for EXE_GD_GRP vs. waitlist and MBI vs. waitlist. Additional information related to the exploration of transitivity is provided in File S6.

**Figure 4:**
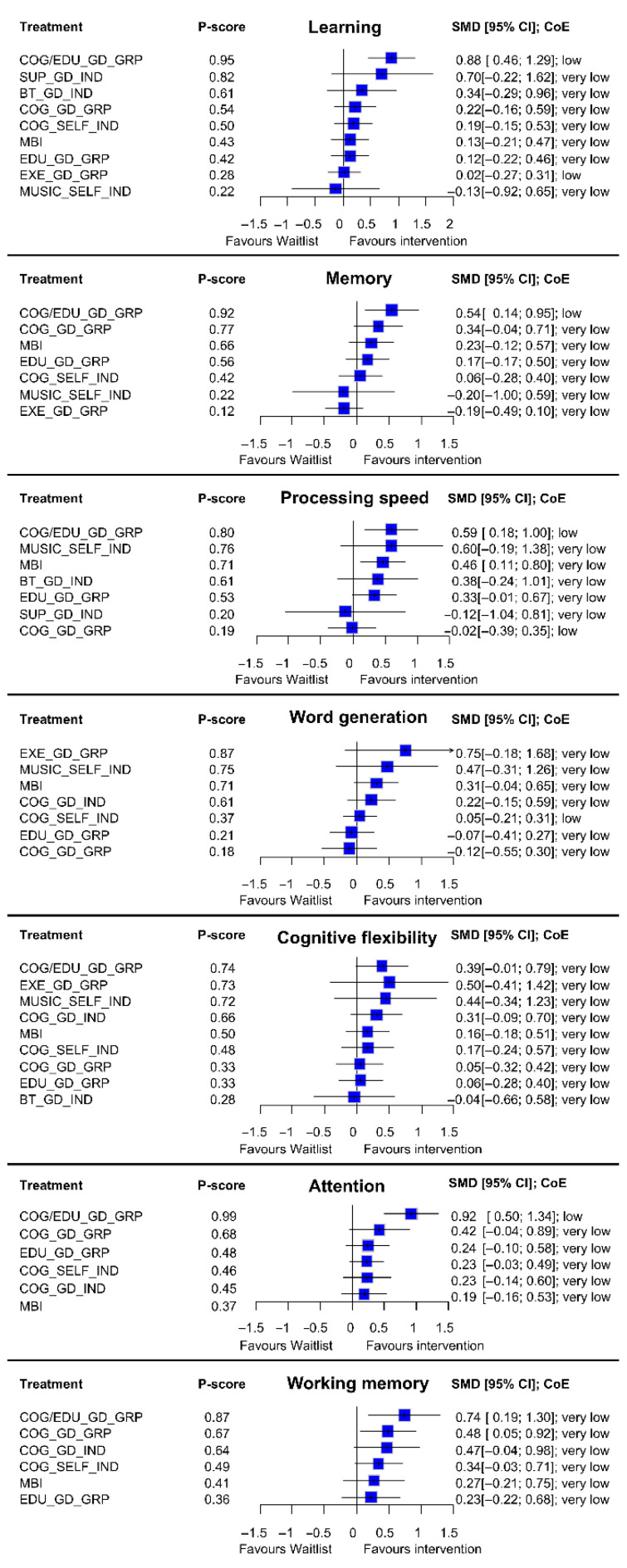
Forest plots presenting standardized mean differences (SMDs; blue boxes) and 95% confidence intervals (CIs; horizontal bars) for each intervention compared with waitlist control, based on network meta-analyses across seven cognitive domains. Each plot reflects pooled effect estimates from eligible studies within a specific cognitive domain. The certainty of evidence (CoE) for each comparison is rated using the CINeMA framework and indicated alongside the point estimates. Interventions are ordered by *p*-score within each domain to facilitate comparison of relative magnitude and precision of effects.

Between-study heterogeneity ranged from zero to low across all NMAs (τ^2^ range = 0.00 to 0.04). Due to sparsity of the evidence, meta-regression analyses were not feasible. Certainty of evidence assessments for all comparisons vs. waitlist are provided in Figure 4 and for all other comparisons in File S8. The most common reasons for downgrading of certainty of evidence were within-study bias, reporting bias, and indirectness.

#### 3.2.1. Learning

Thirteen studies incorporating 933 participants, 10 interventions, and 17 pairwise comparisons were included in the NMA (Figure 2A). Findings driven largely by a single trial suggested that COG/EDU_GD_GRP was the most effective intervention compared to waitlist (SMD = 0.88, 95% CI = 0.46 to 1.29; low certainty) (Figure 4). No other interventions demonstrated a significant difference from waitlist. Amongst all comparisons of active interventions, only COG/EDU_GD_GRP demonstrated significant improvements compared to COG_GD_GRP, COG_SELF_IND, MBI, EDU_GD_GRP, EXE_GD_GRP, and MUSIC_SELF_IND, driven by indirect evidence (File S8).

#### 3.2.2. Memory

Eleven studies incorporating 856 participants, 8 interventions, and 15 pairwise comparisons were included in the NMA (Figure 2B). Findings driven largely by a single trial suggested that COG/EDU_GD_GRP was the most effective intervention compared to waitlist (SMD = 0.54, 95% CI = 0.14 to 0.95; low certainty) (Figure 4). No other interventions demonstrated a significant difference from waitlist. Amongst all comparisons of active interventions, only COG/EDU_GD_GRP and COG_GD_GRP demonstrated significant improvements compared to EXE_GD_GRP, driven by indirect evidence (File S8).

#### 3.2.3. Processing Speed

Twelve studies incorporating 776 participants, 11 interventions, and 18 pairwise comparisons were included in the NMA (Figure 2C). Findings driven largely by a single trial suggested that COG/EDU_GD_GRP was the most effective intervention compared to waitlist (SMD = 0.59, 95% CI = 0.18 to 1.00; low certainty) (Figure 4). As well, MBI demonstrated a significant improvement compared to waitlist control (SMD = 0.46, 0.11 to 0.80; low certainty; *n* = 2 RCTs). Amongst all comparisons of active interventions, only COG/EDU_GD_GRP and MBI demonstrated significant improvements compared to COG_GD_GRP driven by indirect evidence (File S8).

#### 3.2.4. Word Generation

Nine studies incorporating 712 participants, 8 interventions, and 15 pairwise comparisons were included in the NMA (Figure 2D). Findings driven largely by a single trial suggested that EXE_GD_GRP was the most effective intervention compared to waitlist, although the difference was not significant (SMD = 0.75, 95% CI = −0.18 to 1.68; very low certainty) (Figure 4). No interventions demonstrated a significant difference from waitlist. Amongst all comparisons of active interventions, only MBI demonstrated a significant improvement compared to EDU_GD_GRP driven by indirect evidence (File S8).

#### 3.2.5. Cognitive Flexibility

Eleven studies incorporating 740 participants, 10 interventions, and 17 pairwise comparisons were included in the NMA (Figure 2E). Findings driven largely by a single trial suggested that COG/EDU_GD_GRP was the most effective intervention compared to waitlist, although the difference was not significant (SMD = 0.39, 95% CI = −0.01 to 0.79; low certainty) (Figure 4). No interventions demonstrated significant differences compared to either waitlist or active interventions (File S8).

#### 3.2.6. Attention

Eight studies incorporating 742 participants, 7 interventions, and 14 pairwise comparisons were included in the NMA (Figure 2F). Findings driven largely by a single trial suggested that COG/EDU_GD_GRP was the most effective intervention compared to waitlist (SMD = 0.92, 95% CI = 0.50 to 1.34; low certainty) (Figure 4). No other interventions demonstrated a significant difference from waitlist. Only COG/EDU_GD_GRP demonstrated significant improvements compared to other active interventions, including EDU_GD_GRP, COG_SELF_IND, COG_GD_IND, and MBI driven by indirect evidence (File S8).

#### 3.2.7. Working Memory

Eight studies incorporating 626 participants, 7 interventions, and 14 pairwise comparisons were included in the NMA (Figure 2G). Findings driven largely by a single trial suggested that COG/EDU_GD_GRP was the most effective intervention compared to waitlist (SMD = 0.74, 95% CI = 0.19 to 1.30; very low certainty) (Figure 4). Also, COG_GD_GRP demonstrated significant improvements compared to waitlist (SMD = 0.48, 0.05 to 0.92; very low certainty; *n* = 3 RCTs). No interventions demonstrated significant differences compared to other active interventions (File S8).

### 3.3. Results from NMAs at Long-Term Follow-Up

In addition to analyses of data measured immediately post-intervention, analyses using longer-term follow-up data were performed. In general, these NMAs were informed by fewer RCTs, and in some cases, one or more intervention groups were lost from the network given the sparsity of evidence. These losses included the highest-ranking treatment (COG/EDU_GD_GRP) from the analyses presented above, which had been supported by only a single trial in immediate post-intervention analyses, as well as EXE_GD_GRP and COG_GD_IND. Overall, treatment effect estimates and clinical interpretations were relatively similar to those from analyses immediate post-intervention, with minor numerical shifts observed given changes in the underlying data and network geometry. Additional information, including league tables and network diagrams have been reported in File S9.

### 3.4. Summary of Findings Across Outcomes

**Figure 5:**
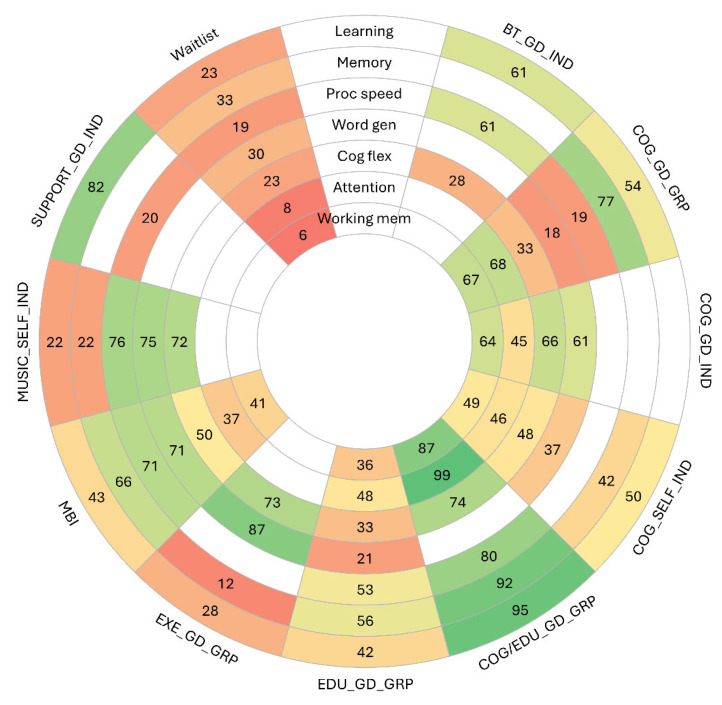
Rank-heat plot of P-scores for interventions across seven cognitive domains: learning, memory, processing speed, word generation, cognitive flexibility, attention, and working memory. P-scores represent the relative ranking of each intervention based on network meta-analytic estimates, ranging from 0 (least effective) to 100 (most effective). Higher P-scores indicate stronger evidence of benefit relative to other interventions in the network. Colour intensity reflects the magnitude of the *p*-score, with green indicating higher ranks and red indicating lower ranks. Blank cells are shown in cases where there was no evidence for the associated intervention.

For all outcomes for which COG/EDU_GD_GRP had data available, COG/EDU_GD_GRP was the top ranked intervention (driven by a single RCT), while waitlist control was consistently one of the lowest ranked interventions (see rank-heat plot provided in Figure 5). Patterns in the observed *p*-score values across outcomes suggest that for the remaining interventions, the benefits of different treatment options were varied dependent upon the domain of cognition under study. Increasingly labour-intensive, multi-dimensional interventions (e.g., guided group interventions) in general may offer additional clinical benefits relative to simpler interventions. The consistently low ranking of the waitlist control group suggests that the administration of interventions, in general, provides some clinical benefits to patients (as opposed to no treatment), while the best choice of intervention may require consideration of the aspects of cognition that require improvement.

### 3.5. Syntheses of Additional Evidence

#### 3.5.1. Abstraction Domain

One study [79] comparing COG_SELF_IND (*n* = 77) to waitlist (*n* = 59) reported data for the abstraction domain using the Delis-Kaplan Executive Function System (D-KEFS) 20 Questions Test total score. There was no difference between groups immediately post-intervention (SMD = 0.11, 95% CI = −0.23 to 0.45; *p* = 0.517). The study authors reported findings from a mixed linear model with time nested within individuals, incorporating data from three time points (baseline, immediate post-intervention, and 20 weeks post-intervention) and found no group*time interaction effect (SMD = 0.10, F = 0.7; *p* = 0.495).

#### 3.5.2. Pharmacological Therapies

Two studies [87,91] evaluated donepezil versus placebo and reported data adjusted for baseline covariates for the learning, memory, processing speed, cognitive flexibility, word generation, and working memory domains. In PWMAs, no significant effects of donepezil were found for any outcome (File S9). One of these studies [87] reported data for the attention domain and found no significant effect of donepezil in repeated measures analysis of covariance.

#### 3.5.3. Other Non-Pharmacological Therapies

One study [94] compared exposure to bright white light (*n* = 20) to exposure to dim red light (*n* = 22) and reported data for processing speed, cognitive flexibility, word generation, and attention. No significant differences between groups were found for any cognitive domain immediately post-intervention: processing speed (mean difference (MD) in z-scores = −0.32, 95% CI = −0.90 to 0.26; *p* = 0.274), cognitive flexibility (MD z-scores = −0.60, 95% CI = −1.44 to 0.34, *p* = 0.156), word generation (MD z-scores = −0.25, 95% CI = −0.90 to 0.40, *p* = 0.439), or attention (MD z-scores = 0.03, 95% CI = −0.62 to 0.68, *p* = 0.926). The study authors reported findings from mixed linear models, incorporating data from three time points (baseline, immediate post-intervention, and 8 weeks post-intervention) and found no significant group*time interaction effects except for visual memory (partial eta-squared = 0.09; F = 3.77, numerator *df* = 2, denominator *df* = 77; *p* = 0.03). Findings for other cognitive domains have been reported in File S9.

## 4. Discussion

Our systematic review incorporating NMAs of interventions for CTRCI identified 18 unique studies of chronic CTRCI related to systemic cancer therapy that encompassed 1593 cancer survivors. We classified the evaluated interventions into ten distinct non-pharmacological treatments, one pharmacological treatment, and one waitlist/usual care control. Across all seven outcomes for which NMA was performed, there were few NPIs that demonstrated significant benefits over waitlist, and evidence supporting these findings was judged of low to very low certainty. Patient education plus cognitive rehabilitation/training in a therapist-guided group setting (i.e., COG/EDU_GD_GRP) was suggested to significantly improve learning, memory, processing speed, attention, and working memory compared to waitlist and some active interventions. However, a single study of 116 participants with potential transitivity issues provided direct evidence for this intervention vs. waitlist [78]. Future trials evaluating this intervention may yield treatment effects that are smaller than those observed in the existing trial or even null, as suggested by 95% prediction intervals. Mindfulness-based interventions also significantly improved processing speed over waitlist with very low certainty of evidence; however, no other NPIs demonstrated significant benefits over the waitlist control for any outcome. Similarly, in PWMAs, donepezil, the only pharmacological therapy evaluated in our included studies, did not demonstrate significant benefits over placebo for any outcome. Overall, findings from NMAs appeared to reinforce several important considerations: (1) provision of some form of intervention for patients offers more benefits than no intervention at all; (2) interventions that are more labour intensive and multi-dimensional may provide greater clinical benefits; and (3) for several interventions, the extent of clinical gains reached may vary depending upon the aspects of cognitive function for which improvements are being sought. Our findings support the clinical utility of multi-dimensional, therapist-guided interventions, particularly those involving cognitive rehabilitation and patient education in group settings.

A recent umbrella review found 64 systematic reviews synthesizing evidence on the effects of NPIs on CTRCI [95]. The umbrella review [95] did not distinguish between reviews focusing specifically on cognitive impairment attributable to systemic cancer therapy and those addressing the general effects of cancer, with or without systemic therapy. However, only nine of the 64 reviews (14%) restricted eligibility to primary studies that exclusively enrolled participants with baseline cognitive deficits. Thus, most existing systematic reviews do not focus strictly on the treatment of established and persistent CTRCI after systemic therapy, as specified by the eligibility criteria of our review. Whether interventions aimed at preventing or treating CTRCI during systemic therapy—or addressing cognitive impairment in patients who have not received systemic therapy—are equally effective for managing CTRCI in patients who have undergone systemic therapy remains unclear. However, the umbrella review concluded that, overall, NPIs were effective in improving CTRCI, with the strongest evidence supporting cognitive training or rehabilitation, while multi-modal or complex interventions showed promise. These general findings are consistent with our review, where COG/EDU_GD_GRP—a complex intervention incorporating cognitive rehabilitation—showed the greatest efficacy, although this was based on a single trial.

A 2016 Cochrane review [19] using similar eligibility criteria to the current review and planned PWMAs; however, most were deemed infeasible by the study authors due to between-study heterogeneity in interventions and outcomes. We identified this as a potential limitation during protocol development and, through collaboration with content experts, developed strategies to optimize data for NMA while maintaining clinical relevance. Guided by the ICCTF’s identification of cognitive domains most affected by CTRCI [18], we defined outcomes that aligned with their recommendations and ensured consistency across available assessment tools. Because some ICCTF-specified domains (e.g., executive function) were broad, we refined them into subdomains with relatively homogeneous neuropsychological tests to minimize clinical heterogeneity and ensure sufficient data for analysis. We limited NMAs to assessment tools recommended by the ICCTF or those validated and commonly used in clinical research with available documentation, reducing clinical heterogeneity and focusing on tools that best measured the domain of interest. Interventions were grouped into clinically defined categories based on the underlying therapeutic principles and clinical context, combining similar therapies to form more robust networks while preserving aspects of clinical context that may modify treatment effects. Finally, we applied statistical methods to optimize data usage, including generation of composite scores when a study reported two tools that assessed one of our outcomes, use of SMDs to incorporate multiple tools per analysis, and inclusion of studies reporting either follow-up or change-from-baseline data.

Regarding systematic reviews that planned NMAs, five have been published to date [21,22,23,24]. However, all included mixed populations, including (a) participants who did and did not receive systemic cancer therapy [20,24] and/or (b) participants who were currently receiving or had previously received systemic cancer therapy [20,21,22,23,24], making direct comparison with findings from the current review difficult. The top-ranked NPIs varied across reviews, likely reflecting differences in study populations, intervention classifications, and outcome definitions/cognitive domains. Moreover, many of these NMAs did not report methods or study characteristics in sufficient detail to enable comprehensive comparisons of populations, interventions, or outcomes. Only one previous NMA [20] appraised the certainty of evidence using an established framework like GRADE (Grading of Recommendations Assessment, Development, and Evaluation) [96] or CINeMA [50]. In summary, while all NMAs support the general benefit of NPIs such as cognitive training/rehabilitation and mindfulness for CTRCI, our review provides a unique and rigorous focus on specific objective cognitive domains in post-treatment cancer survivors with existing CTRCI.

Future trials would benefit greatly from the development of a core outcome set for CTRCI, along with recommendations for standardized assessment tools. While the ICCTF published formal guidance in 2011 [18], it has not been updated and may no longer be broadly recognized or easily accessible to researchers. In our review, several eligible studies published after 2011 were ultimately excluded because they did not assess an objective cognitive function domain of interest using an eligible tool [97,98,99,100,101,102,103,104,105,106]. Similarly, a recent systematic review of CTRCI in lung cancer patients found that only 33% of studies applied ICCTF criteria to identify CTRCI through neuropsychological testing [14]. Other systematic reviews have highlighted the large number and heterogeneity of objective cognitive domains and assessment tools used in clinical research [107,108]. The development of an updated core outcome set, endorsed and promoted by an international initiative such as COMET (Core Outcome Measures in Effectiveness Trials: https://www.comet-initiative.org/), could provide much-needed standardization and help ensure the methodological consistency of future clinical trials investigating CTRCI.

Future clinical trials could be strengthened by explicitly considering key effect modifiers within their patient populations. Consistent reporting of baseline comorbidities—such as fatigue, anxiety, and depression—using standardized measures would facilitate comparison of populations across trials and enhance the assessment of heterogeneity in evidence syntheses. Furthermore, the influence of chemotherapy class and cumulative dose on the effectiveness of CTRCI interventions remains unclear, as these factors cannot yet be disentangled from overall treatment effects. Stratifying randomization by chemotherapy class, adjusting for chemotherapy class and dose in statistical models, and reporting subgroup analyses based on chemotherapy exposure would substantially improve our understanding of the determinants of CTRCI and the effectiveness of its treatments.

### Strengths and Limitations

Our systematic review is distinguished by its focused eligibility criteria, including only cancer survivors with persistent CTRCI following systemic therapy; a comprehensive literature search yielding nearly 20,000 citations, with judicious use of AI to expedite screening; engagement of content experts to rigorously classify interventions, cognitive domains/outcomes of interest, and the neuropsychological tools that best assess those outcomes; extensive assessment of transitivity using both subjective and semi-objective approaches; and a structured appraisal of certainty of evidence through the CINeMA framework [50].

Limitations arose partly from issues inherent in the available evidence and partly from methodological choices aimed at reducing clinical heterogeneity and optimizing the data for synthesis. Most of the trials included in our review focused on breast cancer survivors, limiting the generalizability of findings to other cancer populations or to male patients. Several included studies provided insufficient reporting of intervention details and neurocognitive test characteristics, which may have led to misclassification of interventions or misassignment of assessment tools to outcome domains. In addition, several of the reported interventions were multidimensional and may have involved components spanning more than one classification group. Elevated ROB across all trials reduced the certainty of all evidence to low to very low. Our focus on post-systemic therapy cancer survivors and the inclusion of only studies that assessed objective cognitive function using established neurocognitive tools helped reduce clinical heterogeneity but also limited the body of evidence available for synthesis. Also, the normative data used to generate t-scores for composite outcomes may not have been fully matched to the sex, age, or educational level of each study population, although the impact of this on our findings was likely minimal [109]. Although publication bias could not be formally assessed, selective reporting and small-study effects remain possible due to the presence of small pilot/ feasibility studies. We reflected this limitation by rating all comparisons as having “some concerns” for reporting bias in CINeMA. Finally, network geometry, sparse data, and insufficient reporting of key effect modifiers in trials precluded meta-regression and sensitivity analyses to further explore clinical heterogeneity.

## 5. Conclusions

This systematic review with NMA uniquely focused on interventions for persistent CTRCI in adult non-central nervous system cancer survivors who had completed systemic therapy, using objective cognitive function outcomes. Across seven cognitive domains, few NPIs demonstrated significant benefits over waitlist, with evidence consistently rated as low to very low certainty. Specifically, patient education plus cognitive rehabilitation/training in a therapist-guided group setting (COG/EDU_GD_GRP) was the top-ranked intervention, showing significant improvements in learning, memory, processing speed, attention, and working memory; however, its efficacy was predominantly supported by a single study, and future trials may yield smaller or null effects. While MBI also improved processing speed, donepezil—the only pharmacological therapy evaluated—showed no significant benefits over placebo for any outcome. The overall evidence base was limited by the stringent eligibility criteria, insufficient reporting of intervention and neurocognitive test details, and pervasive risk of bias in included studies, contributing to the low certainty of findings.

Moving forward, standardizing a core outcome set for CTRCI trials with recommended assessment tools and improving comprehensive reporting of patient and intervention characteristics are critical to enhance the consistency and reliability of future research in this vital area of cancer survivorship. Until stronger evidence emerges, clinicians may consider recommending structured, therapist-guided cognitive rehabilitation programs—particularly those that incorporate educational components—for cancer survivors experiencing CTRCI.

## Figures and Tables

**Table 1:** Characteristics of 14 studies included in network meta-analyses at immediate post-intervention.

Study Country Randomized Controlled Trial (RCT) or Feasibility Trial Funding	Study Objective	Study Dates	Interventions (Sample Size Per Arm in Network Meta-Analyses)	Intervention Duration; Follow-up Time(s) Since Intervention	Cognitive Impairment Eligibility Criteria	Mean (SD) Months Since End of Chemotherapy; Mean (SD) Age (Years); % with Breast Cancer	Systemic Cancer Therapies: Chemo, Radiation, Hormone, Targeted	Education	Baseline Comorbidities: Cognitive Impairment; Depression; Anxiety; Fatigue
Lengacher et al., 2024 [88] USA RCT Non-industry	“…to evaluate if BCS [breast cancer survivors] assigned to either the MBSR(BC) [Mindfulness-Based Stress Reduction for breast cancer survivors], Breast Cancer Education Support (BCES), or Usual Care (UC) regimens experienced greater improvements at 6, 12, and 26 weeks on objective and subjective cognitive performance”	October 2015 to July 2020 ^†^	MBI (*n* = 81) EDU_GD_GRP (*n* = 79)	6 weeks; 0 and 5 months	A positive response to at least one of two scaled questions from the European Organization for Research and Treatment of Cancer Quality of Life questionnaire (EORTC-QLQ): (1) “Please rate on a scale from 0 to 10, the difficulty level you have in concentrating on things, like reading a newspaper or watching television?”, (2) “Please rate on a scale from 0 to 10, the difficulty level you have in remembering things.” (0 = no difficulty, 10 = very difficult)	~24.1 (NR); 56.4 (10.6); 100%	100% 77% Not reported Not reported	Less than a college degree: 21.7%, Some college or AA degree: 28.8%, College degree: 25.0%, Graduate/ professional school: 23.6%	Severe; Mild; Severe; Mild
Melis et al., 2023 [89] Belgium RCT Non-industry	…to evaluate “the potential of MBI to reduce CTRCI [cancer-related cognitive impairment]”	1 Oct 2018 ^‡^	Waitlist (*n* = 32) MBI (*n* = 36) EDU_GD_GRP (*n* = 31)	8 weeks; 0 and 3 months	Significant cognitive complaints (Cognitive Failures Questionnaire [CFQ] total score > 42.9 [mean + 1 SD] or at least two of the four extra CFQ questions >mean 1 + SD	25 (14.4); 48.4 (8.7); 100%	100% 73% 71% Not reported	Secondary school: 26.5%, Higher education: 73.5%	Severe; Not reported; Not reported; Not reported
Vardy et al., 2023 [93] Australia RCT Non-industry	“…to evaluate two cognitive rehabilitation programs used in non-cancer populations against standard-of-care control in solid tumour survivors self-reporting cognitive impairment after chemotherapy”	September 2015 to November 2020 ^§^	Waitlist (*n* = 16) COG_GD_GRP (*n* = 20) EDU_GD_GRP (*n* = 18)	6 weeks; 0 and 6 months	Self-reported cognitive change on the 2-item EORTC-QLQ-C30 Cognitive Functioning scale as “quite a bit” or greater in one or both domains	26.3 (11.9); 54.5 (8.8); 92.3%	100% 71% 72% Not reported	mean (SD) years: 14.4 (3.3)	Severe; None; None; Mild
Cherrier et al., 2022 [78] USA RCT Non-industry	…to examine “the effectiveness of a behavioral skills training intervention to improve objectively measured cognitive performance as well as individual participant defined cognitive symptoms in cancer survivors”	Not reported	Waitlist (*n* = 47) COG/EDU_GD_GRP (*n* = 49)	7 weeks; 0 months	Subjective concern about declines in cognitive functioning related to a diagnosis of cancer and/or cancer-related treatment (obtained through phone screening by requiring a Yes response to the question: “Do you have concerns about your memory or other thinking abilities related to your cancer or cancer treatment?”	52.8 (63.6); 59.2 (10.4); Not reported	89% 54% Not reported Not reported	mean (SD) years: 16.8 (2.6)	Severe; Mild; None; Moderate
Koevoets et al., 2022 [86] Netherlands RCT Non-industry	…to investigate “whether exercise training improves cognition in chemotherapy-exposed breast cancer patients 2–4 years after diagnosis”	December 2016 and September 2020 ^¶^	Waitlist (*n* = 82) EXE_GD_GRP (*n* = 82)	26 weeks; 0 months	Needed to self-report cognitive problems after cancer diagnosis, which was confirmed by lower than expected performance on neuropsychological testing	31.2 (7.8); 52.3 (8.6); 100%	100% 75% 61% 21%	High: 43.1%, Middle: 55.8%, Low: 0, Missing: 1.1%	Moderate; None; Mild; None
Dos Santos et al., 2020 [80] France RCT Non-industry	“…to evaluate the impact of computer-assisted CR [cognitive rehabilitation] on subjective and objective cognition, QOL [quality of life], anxiety, and depression among cancer patients treated with chemotherapy and reporting cognitive complaints”	September 2012 to July 2017 ^†^	Waitlist (*n* = 51) COG_GD_IND (*n* = 48) COG_SELF_IND (*n* = 44)	12 weeks; 0 months	Reported cognitive complaint (during or within five years of chemotherapy completion)	11.4 (2.3); 51.3 (10.3); 83.8%	100% 79% 59% Not reported	Primary school: 5.4%, Middle school: 16.2%, High school: 16.8%, University: 50.3%, Unknown: 11.4%	Severe; Mild; Moderate; None
Henneghan et al., 2020 [84,85] USA Feasibility/pilot Non-industry	“...to determine the feasibility of delivering an eight-week daily KK [Kirtan Kriya] intervention program remotely to BCS” and “...to evaluate the preliminary effects of the program on cognitive function (performance and perceived), psychological functioning (anxiety, depression, stress), fatigue, and self-efficacy in BCS compared to classical music listening (ML)”	October 2018 to July 2019 ^†^	MUSIC_SELF_IND (*n* = 15) MBI (*n* = 16)	8 weeks; 0 and 2 months	Reported cognitive deficits (a minimum of five cognitive symptoms occurring “sometimes” or more, per the Perceived Deficits Questionnaire	31.4 (19.4); 49.5 (9.9); 100%	100% 84% Previous: 65% Current: 67% 42%	Bachelors or higher: 71.0%	Severe; None; None; Mild
Van der Gucht et al., 2020 [92] Belgium Feasibility/pilot Non-industry	“…to investigate the effect of a blended-care mindfulness-based intervention (MBI) on chemotherapy-related cognitive impairment and functional brain changes”	Not reported	Waitlist (*n* = 14) MBI (*n* = 12)	8 weeks; 0 and 3 months	Had significant cognitive complaints, as measured by the CFQ: a CFQ total score > 42.9 or a score greater than the accepted mean score ± 1 SD on ≥2 CFQ extra questions.	19 (Not reported); 45.5 (6); 100%	100% 36% 79% Not reported	Secondary school: 45%; Higher education degree: 45%; Never finished secondary school: 10%	Severe; Moderate; Not reported; Mild
Campbell et al., 2018 [76] Canada Feasibility/pilot Non-industry	…to test “the effect of a 24-week aerobic exercise intervention compared to usual lifestyle control on measures of cancer-associated cognitive impairment in early stage BCS reporting persistent cognitive concerns”	January 2011 to June 2013 ^†^	Waitlist (*n* = 9) EXE_GD_GRP (*n* = 10)	24 weeks; 0 and 6 months	Self-report cognitive impairment	11.2 (6.7); 52.4 (6.2); 100%	100% 90% Not reported Not reported	Vocational training/some college or less: 31.6%, College graduate: 43.4%, Some postgraduate or more: 21.1%	Severe; None; None; Mild
Damholdt et al., 2016 [79] Denmark RCT Non-industry	…to examine “effects of web-based cognitive training in a large sample of breast cancer survivors, applying a broad cognitive training program with baseline and short- and long-term telephone-based neuropsychological assessments”	March 2013 to December 2014 ^†^	Waitlist (*n* = 59) COG_SELF_IND (*n* = 77)	6 weeks; 0 and 5 months	Subjective complaints of cognitive impairment and scoring above the sample median (≥27) on the CFQ	Months since diagnosis: 55.4 (21.9); 54.8 (8.6); 100%	83% 85% 69% Not reported	Municipal primary and lower secondary school, incl. apprenticeships: 24.2%, Short (<3 years): 21.7%, Medium (3–4 years): 33.1%, Long (+5 years): 19.7%	Severe; None; None; Not reported
Ferguson et al., 2016 [83] USA RCT Non-industry	…to evaluate Memory and Attention Adaptation Training (MAAT), a cognitive behavioral therapy, compared with a supportive therapy control	Not reported	SUP_GD_IND (*n* = 14) BT_GD_IND (*n* = 22)	8 weeks; 0 and 2 months	Reported cognitive problems attributed to chemotherapy; A score of ≤10 on the FACT-Cog Impact of Quality of Life Scale	52.6 (46.3); 54.6 (12.12); 100%	100% Not reported Not reported Not reported	mean (SD) years: 15.6 (2.8)	Severe; None; None; Moderate
Ercoli et al., 2015 [81] USA RCT Non-industry	…to evaluate “the efficacy of a cognitive rehabilitation (CR) intervention compared with a waitlist (WL) control condition on cognitive complaints, neuropsychological and brain functioning in breast cancer survivors (BCS)”	January 2012 and April 2013 ^†^	Waitlist (*n* = 16) COG_GD_GRP (*n* = 28)	5 weeks; 0 and 2 months	Self-reported cognitive difficulties interfering with everyday activities	Months since diagnosis: 33.6 (13.2); 53.8 (8.2); 100%	77% 75% Currently: 71% 26%	Less than college: 25%, College graduate: 20.8%, Post-college graduate: 54.2%	Mild; None; Not reported; Not reported
Cherrier et al., 2013 [77] USA RCT Non-industry	…to examine “the effectiveness of a group-based cognitive rehabilitation intervention in cancer survivors”	Not reported	Waitlist (*n* = 16) COG_GD_GRP (*n* = 12)	7 weeks; 0.25 and 0.5 months	Subjective concern about declines in cognitive functioning related to a diagnosis of cancer and/or cancer related treatment, which was assessed by a Yes response to the question “Do you have concerns about your memory or other thinking abilities following cancer treatment?”	58.1 (12); 58.9 (2.4); Not reported	89% 46% Not reported Not reported	mean (SD) years: 17.1 (0.5)	Severe; Mild; None; Severe
Ferguson et al., 2012 [82] USA RCT Non-industry	“To evaluate the efficacy of a brief cognitive-behavioral therapy (CBT) that is being developed for management of cognitive dysfunction following chemotherapy among breast cancer survivors”	Not reported	Waitlist (*n* = 21) BT_GD_IND (*n* = 19)	8 weeks; 0 and 2 months	Complaint of memory and attention problems following chemotherapy	>18; 50.3 (6.4); 100%	100% Not reported Not reported Not reported	mean (SD) years: 16.4 (2.4)	Mild; Not reported; Not reported; Not reported

The table summarizes key features of each study. Methods to determine severity of baseline comorbidities have been described in File S2. AA = associate’s degree; BC = breast cancer; BCES = breast cancer education support; BCS = breast cancer survivor; BT_GD_IND = guided one-on-one behavioural therapy; CBT = cognitive behavioural therapy; CFQ = Cognitive Failures Questionnaire; COG/EDU_GD_GRP = guided cognitive therapy/rehabilitation and patient education in a group setting; COG_GD_GRP = cognitive therapy/rehabilitation in a guided group setting; COG_GD_IND = guided one-on-one cognitive therapy/rehabilitation; COG_SELF_IND = self-guided cognitive therapy/rehabilitation; CR = cognitive rehabilitation; CTRCI = cancer-related cognitive impairment; EDU_GD_GRP = guided patient education in a group setting; EORTC-QLQ = European Organization for Research and Treatment of Cancer Quality of Life questionnaire; EXE_GD_GRP = guided physical exercise in a group setting; FACT-Cog = Functional Assessment of Cancer Therapy-Cognitive Function; KK = Kirtan Kriya; MAAT = Memory and Attention Adaptation Training; MBI = mindfulness-based intervention either self-guided or in a guided group setting; MBSR(BC) = mindfulness-based stress reduction for breast cancer survivors; ML = music listening; MUSIC_SELF_IND = self-guided music therapy; QOL = quality of life; RCT = randomized controlled trial; SD = standard deviation; SUP_GD_IND = guided one-on-one patient support (e.g., listening, sharing); UC = usual care; WL = waitlist ^†^ Enrolment or recruitment date; ^‡^ Trial start date; ^§^ Intervention administration dates. ^¶^ Data collection dates with mild (*n* = 5 [76,84,88,92,93] of 10), moderate (*n* = 2 [78,83] of 10), and severe (*n* = 1 [77] of 10) fatigue reported. The estimated study-level average number of years of education ranged from 13.0 to 17.1 years (mean = 15.1 years).

## Data Availability

The dataset extracted and analyzed during this study is available as a Supplementary File submitted with this manuscript.

## Supplementary Materials

The following supporting information can be downloaded at: https://www.mdpi.com/article/10.3390/cancers17213430/s1: File S1: Protocol Amendments. File S2: Detailed Review Methods. File S3: Literature Search Methods and Strategies. File S4: Detailed Methods for CINeMA Certainty of Evidence Appraisal. File S5: Articles Excluded During Full Text Screening. File S6: Findings from Transitivity Exploration. File S7: Study-level Risk of Bias Appraisals. File S8: Network meta-analysis-supporting statistical data from immediate post-intervention analyses and certainty of evidence (CINeMA) appraisals. File S9: Additional Findings. A spreadsheet of all extracted data from the review is also provided.
