# Supplementary material for: Comparative Effectiveness of Interventions to Treat Cancer Treatment-Related Cognitive Impairment in Adult Cancer Survivors Following Systemic Therapy: A Systematic Review with Network Meta-Analyses
Source: Cancers (Basel). 2025 Oct 26;17(21):3430. doi: 10.3390/cancers17213430 (PMC12609492; doi:10.3390/cancers17213430)
Supplement: Supplementary file 1 [file cancers-17-03430-s001.zip › cancers-3917557-Supplement Files S1 through S9 Oct22_2025.pdf]

cancers

MDPI

Supplement, Files S1 – S9

File S1: Protocol Amendments

File S2: Detailed review methods

- Detailed study eligibility criteria
- AI screening methods
- Cognitive function assessment tools of interest
- Data elements extracted
- Intervention classification
- Methods to standardize measures of baseline comorbidities and education attained
- Composite outcome data generation

File S3: Literature search methods and strategies

- Original search strategies; grey literature search methods

File S4: Detailed methods for CINeMA certainty of evidence appraisal

File S5: Articles excluded during full-text screening

File S6: Findings from transitivity exploration

File S7: Study-level risk of bias appraisals

File S8: Network meta-analysis: supporting statistical data from immediate post-intervention analyses and certainty of evidence (CINeMA) appraisals

- Primary network meta-analyses immediate post-intervention: findings for all treatment comparisons; explorations of heterogeneity; network meta-analysis: sensitivity analyses; publication bias; tests of inconsistency; certainty of evidence (CINeMA) appraisals.

File S9: Additional findings

- Results from longer-term follow-up network meta-analyses
- Studies evaluating pharmacological interventions

Table of abbreviations found in supplemental files

Code	Treatment
BT_GD_IND	Guided one-on-one behavioural therapy
COG_GD_GRP	Cognitive therapy/rehabilitation in a guided group setting
COG_GD_IND	Guided one-on-one cognitive therapy/rehabilitation
COG_SELF_IND	Self-guided cognitive therapy/rehabilitation
EDU_GD_GRP	Guided patient education in a group setting
COG/EDU_GD_GRP	Guided cognitive therapy/rehabilitation and patient education in a group setting
EXE_GD_GRP	Guided physical exercise in a group setting
MBI	Mindfulness-based intervention either self-guided or in a guided group setting
MUSIC_SELF_IND	Self-guided music therapy
SUP_GD_IND	Guided one-on-one patient support (e.g., listening, sharing)

WL	Waitlist or care as usual

File S1: Protocol amendments

The following amendments were made to the planned review following publication of our protocol.

- We used the AI/ML tool in DistillerSR to exclude 6,577 references without a human reviewer after having reviewed 10,417 in duplicate and finding only three potentially relevant abstracts in the last 6,481 references screened.
- We assessed the risk of bias due to baseline imbalances between groups in key confounders for the data that we used in our analyses and not necessarily how the researchers analyzed (i.e., if they reported adjusted analyses and we used their raw data, and there were imbalances between groups at baseline, we assessed unclear/high ROB for the raw data that we used, even if they had reported an adjusted analysis to compensate for the imbalances). Therefore, our assessments reflect risk of bias in the data and not necessarily in the study methods. See “Risk of bias and certainty of evidence assessments” section in main text.
- To limit the risk of overestimating precision, we used final follow-up sample sizes in analyses because most studies experienced attrition over the study period.
- As outlined in the main text (see section titled ‘Network meta-analysis feasibility assessment’), we used a clinical approach that was informed by the available data to derive the final outcomes of interest. Also, as outlined in ‘Assessment tools of interest,’ we excluded some neuropsychological tests based on consultation with our clinical experts.
- Adherence was removed as an outcome of interest due to insufficient outcome data.
- We combined the waitlist and care-as-usual control groups together when structuring evidence networks, but considered provision of supportive or educational care to be separate active interventions.
- In addition to subjective transitivity assessments (e.g., box plots), we used a recently published methodology[1] to semi-objectively assess non-statistical heterogeneity/transitivity by calculating and visually portraying dissimilarities between treatment comparisons in study-level participant and methodological characteristics.
-

File S2: Detailed review methods

Detailed study eligibility criteria

PICOS domain	Inclusion criteria
**Population**	Adults (≥ 18 years) who were diagnosed with cancer of any type (except central nervous system (CNS)-related tumors/metastases) during adulthood and who received prior systemic treatment (i.e., chemotherapy, hormonal/endocrine therapy, or immunotherapy), alone or in combination with other treatments (e.g., concurrent radiotherapy), and who experienced cancer treatment-related cognitive impairment (CTRCI) as defined by any criteria.
**Intervention**	Interventions of any dose, frequency, or duration to treat existing CTRCI (i.e., not prevent future CTRCI), including the following, alone or in combination:<br>• Psychological interventions: cognitive behavioral therapy (CBT), cognitive rehabilitation or training, transcranial direct current stimulation, etc.<br>• Pharmacological interventions: donepezil, methylphenidate, modafinil, armodafinil, erythropoietin, fluoxetine, memantine, ramipril, lithium, pioglitazone, etc.<br>• Other interventions: exercise programs, mindfulness-based stress reduction (MBSR), music therapy, Tai chi/qigong, yoga, acupuncture, light therapy, herbal supplements, nutraceuticals, etc.<br><br>We excluded studies that did not report sufficient intervention detail (e.g., missing descriptions of psychological interventions, missing dosages of pharmacological interventions).
**Comparator**	Waitlist, placebo, no treatment, or any of the active interventions above
**Outcomes**	Objective measures of at least one of the following 14 cognitive domains or subdomains: verbal learning, verbal memory, visual learning, visual memory, processing speed, complex psychomotor speed, executive function (overall, conceptual knowledge, mental flexibility/task switching, abstraction, inhibition, language/fluency), attention, or working memory. For final inclusion in the review, studies must have measured one of the above outcomes using one or more of the assessment tools of interest (see table below).
**Study design**	Parallel-group and cross-over randomized controlled trials
**Language and publication type**	English or French; not a conference abstract, letter, commentary, or trial registration

AI screening methods

Given the high yield of the search process (> 17,000 citations), study selection at Level 1 was supported by artificial intelligence/machine learning (AI/ML) tools within the DistillerSR® platform[2]. An initial set of 200 citations, of which several were known to be relevant, were screened by two reviewers and used to train the AI/ML tool to identify potentially relevant citations. Following the training exercise, relevance scores for the remaining citations were generated by the AI/ML tool, reflecting the probability of meeting Level 1 eligibility criteria. Dual independent screening of the remaining citations proceeded in order from high to low relevance score, with the AI/ML tool adjusting relevance scores and reordering citations after each subsequent set of 200 citations had been fully reviewed. Conflicts were resolved frequently throughout this process to optimize continued training of the AI/ML tool. By prioritizing screening of the citations most likely to be relevant, the rate at which citations meeting Level 1 eligibility criteria were identified fell over

time, and the proportion that we had found of the predicted relevant references estimated by the AI/ML tool increased. When this proportion reached 96% (218 of 227 predicted relevant references found) the AI/ML tool was employed to act as a reviewer to exclude the remaining references (n = 13,704). These references were screened by a single human reviewer to identify any citations inadvertently excluded by the AI/ML tool. Citations found through the search update in 2024 were ranked by relevance score and, given the low volume, all were screened by human reviewers. An automated check for screening errors was conducted using the AI audit tool following completion of Level 1 screening.

Cognitive function assessment tools of interest

ICCTF-recommended domain	**Review outcome**	**Assessment tools of interest within review subdomain**	**Number of studies reporting data immediately post-intervention**
Learning and memory	**Learning**	Hopkins Verbal Learning Test-Revised (HVLT-R): immediate or total recall	7 (5 in NMA)
		Rey Auditory Verbal Learning Test (RAVLT): immediate or total recall	6 (6 in NMA)
		California Verbal Learning Test (CVLT): total	2 (2 in NMA)
		Story Recall: immediate recall	0
		Brief Visuospatial Memory Test-Revised (BVMT-R): total recall	3 (3 in NMA)
	**Memory**	HVLT-R: delayed recall	7 (5 in NMA)
		RAVLT: delayed recall	6 (6 in NMA)
		Story Recall: delayed recall	1 (1 in NMA)
		BVMT-R: delayed recall	3 (3 in NMA)
		ROCF: delayed recall	1 (0 in NMA)
Processing speed	**Processing speed**	Trail Making Test (TMT): Part A	11 (6 in NMA)
		Stroop test: Color score, Word score, or Color-word score	1 (1 in NMA)
		Delis-Kaplan Executive Function System (D-KEFS) Color-word inhibition: Color naming, Word reading, or Color + Word	1 (0 in NMA)
		Symbol digit Modalities Test (SDMT)	2 (2 in NMA)
		Wechsler Adult Intelligence Scale (WAIS)-III: Digit Symbol-Coding	6 (5 in NMA)
		WAIS-IV: Coding	1 (0 in NMA)
Executive function	**Cognitive flexibility**	TMT: Part B	12 (9 in NMA)
		RAVLT: interference	0
		D-KEFS Color-word switching	1 (1 in NMA)
		D-KEFS Color-word inhibition	1 (0 in NMA)
		D-KEFS Color-word interference	1 (1 in NMA)

ICCTF-recommended domain	**Review outcome**	**Assessment tools of interest within review subdomain**	**Number of studies reporting data immediately post-intervention**
		Stroop test: interference	3 (3 in NMA)
		Wisconsin Card Sorting Test (WCST): perseverative errors	1 (1 in NMA)
	**Word generation**	Controlled Oral Word Association Test (COWAT): total letters	8 (6 in NMA)
		COWAT: category animal	1 (1 in NMA)
		D-KEFS: verbal fluency	1 (0 in NMA)
		D-KEFS: animals	1 (0 in NMA)
		F-A-S test and various “letter fluency tests” and “verbal fluency tests” for different languages	3 (3 in NMA)
		Animal naming other than COWAT or D-KEFS	2 (2 in NMA)
	**Abstraction**	D-KEFS 20 Questions Test: abstract, total	1 (no NMA)
Attention	**Attention**	WAIS-III Digit Span: forward	6 (5 in NMA)
		WAIS-IV Digit Span: forward	3 (2 in NMA)
		Wechsler Memory Scale (WMS)-III Digit Span: forward	1 (1 in NMA)
		WMS-III Spatial Span: forward	1 (1 in NMA)
Working memory	**Working memory**	Paced Auditory Serial Addition Test (PASAT) #2	1 (1 in NMA)
		WAIS-III Digit Span: backward	5 (3 in NMA)
		WAIS-IV Digit Span: backward	2 (2 in NMA)
		WAIS-III Letter Number Sequencing	4 (4 in NMA)
		WAIS-IV Digit span: ordering	1 (1 in NMA)
		WMS-III Digit Span: backward	0
		WMS-III Spatial Span: backward	0

Data elements extracted

Data category	Data elements
Study characteristics	First author’s last name, year of publication, country of conduct, funding source(s), declaration of conflicts of interest (yes/no), availability of protocol (yes/no), study objective, inclusion/exclusion criteria, study dates, timing of randomization with respect to cancer treatment completion, duration of intervention, follow-up times reported, study limitations as reported by authors, and author conclusions.

Data category	Data elements
Patient demographics (by group unless otherwise stated)	Overall and group-level sample sizes, cancer type, cancer stage, baseline measures of inflammation, types of systemic cancer treatment received, prior oncological surgical intervention, cancer treatment goal (curative vs palliative), age, percent female, race/ethnicity, marital status, socioeconomic status (e.g., employment status, household income), education level, comorbidities present (including menopausal status), baseline global cognitive function (i.e., test that was used to screen for study eligibility), baseline measures of related psychological conditions (i.e., depression, anxiety, fatigue, distress), and baseline selective serotonin reuptake inhibitor (SSRI) or other anti-depressant use.
Intervention/control characteristics	Intervention type (i.e., psychological, pharmacological, or other); non-pharmacological intervention (NPI) description, duration, frequency, and setting; pharmacological dose, duration, frequency, and route of administration; and control description (e.g., waitlist; for NPIs, whether there was contact with research team during follow-up).
Outcome data	Scale/subscale used to measure construct, follow-up time since completion of intervention, group level outcome data (i.e., baseline sample size and mean [standard deviation (SD)] score, follow-up sample size and mean (SD) score, within-group mean (SD) change, overall effect of time across all groups), between-group comparisons [group sample sizes at follow-up, effect estimate (e.g., Cohen's d, mean difference) and standard error (SE), 95% confidence interval (CI), and/or p-value], global between-group test if > 2 groups, the statistical test(s) used for between-group testing, whether an adjusted analysis was reported, and how missing data were handled (e.g., complete case analysis, last observation carried forward, linear mixed model allowing missing data).

Intervention classification

Inconsistencies in intervention classification and reporting are well-documented in the literature[3,4]. Challenges in this area arise given that interventions are typically complex, and can contain multiple, often interacting components. Several competing frameworks have been proposed to address these issues[5-7], though often this guidance is focused only on a specific intervention format (e.g., only behavioural interventions). These frameworks can also be difficult to apply given that research publications often provide few details about the interventions being evaluated or their methods of delivery. The present review's approach to intervention classification was to identify—from the details provided by the study authors—the major intervention component that was being evaluated in each study (e.g., cognitive training, behavioural modification). Recognizing that the interventions were often multidimensional, with components from different methodologies being incorporated together in the same intervention, the rationale for these groupings stemmed from the objective to group interventions together that relied on common guiding principles. For example, all mindfulness-based interventions were grouped together as all involved components of mindfulness-based approaches to improving psychological and mental well-being. A distinction was made between pharmacological and non-pharmacological interventions (NPI), with NPIs being further subdivided based on their major intervention component, whether they were guided or self-guided, and whether they were group-based or one-on-one. These further splits allowed for a more nuanced examination of how interventions that incorporated components of peer and/or instructional support (i.e., group-based and/or guided) compared to those interventions that were more individualized and allowed for a deeper and more personalized experience (i.e., self-guided and/or one-on-one). These groupings/splits were made based on the details provided by the study authors in each publication. Some interventions may fit in more than one group. Using the details provided, and with a primary focus on determining

the major underlying intervention component, we sought to achieve a balance in our groupings between the principles of each intervention, while considering the geometry of the network and efforts to avoid data sparsity.

Methods to standardize measures of baseline comorbidities and education attained

Given the substantial heterogeneity of reporting formats for various key effect modifiers (i.e., baseline cognitive function, depression, anxiety, fatigue, education level), we estimated standardized study-level measures for each covariate. For cognitive measures, when arm-level means were reported, the study-level mean was calculated as the sample size weighted average. Study-level means were then compared to mild, moderate, and severe cutoffs for the relevant assessment tool that were developed in consultation with our content experts and the published literature (see **tables** below). Education level data had highly heterogeneous reporting formats that were often country specific. Where categorical data were reported (e.g., elementary school, high school, college/university degree), we estimated the number of years of education in each category, then calculated the weighted average years of education across all categories of a study. Estimates of the number of years for each educational category are presented in the table below.

Table. Cutoffs for baseline cognitive impairment

Scale	Mild Impairment Cutoff	Moderate Impairment Cutoff	Severe Impairment Cutoff	Cutoffs Published?
**CFQ (Cognitive Failures Questionnaire)**	~33–38 (upper quartile)	~39–44 (above 1 SD)	>44 (upper 10–15%)	No
**Wechsler Test of Adult Reading (WTAR)**	<90 (below avg)	<85 (low avg)	<70 (very low)	No
**MDASI-MM (Memory Module)**	1–3 (mild)	4–6 (moderate)	7–10 (severe)	Yes
**FACT-Cog PCI**	54–63 (mild)	44–53 (moderate)	<44 (severe)	Yes
**FACT-Cog v3 Total**	125–140 (mild)	105–124 (moderate)	<105 (severe)	No
**PAOFI**	~10–19 (mild)	20–29 (moderate)	≥30 (severe)	No
**MASQ**	105–119 (mild)	90–104 (moderate)	<90 (severe)	No

Table. Cutoffs for baseline depression

Scale	Mild Depression Cutoff	Moderate Depression Cutoff	Severe Depression Cutoff	Cutoffs Published?
HADS Depression	8–10	11–14	15–21	Yes
PHQ-9	5–9	10–14	15–27	Yes
CES-D	16–26	27–39	≥40	Yes
PROMIS-Depression (T-scores)	T-score 55–59.9	T-score 60–69.9	T-score ≥70	Yes
PROMIS-Depression (raw scores assuming a 5-point Likert scale)	2.0-2.9	3.0-3.9	4.0-5.0	
DASS-21 (Total)	10–13	14–20	21+	Yes
DASS-21 Depression	10–13	14–20	21+	Yes
BDI	10–18	19–29	30–63	Yes
BDI-II	14–19	20–28	29–63	Yes

Table. Cutoffs for baseline anxiety

Scale	Mild Anxiety Cutoff	Moderate Anxiety Cutoff	Severe Anxiety Cutoff	Cutoffs Published?
HADS Anxiety	8–10	11–14	15–21	Yes
BAI (Beck Anxiety Inventory)	8–15	16–25	26–63	Yes
STAI-Trait	39–45	46–52	≥53	Yes
PROMIS Anxiety (T-score)	55–59	60–69	≥70	Yes
State Anxiety Inventory (S-AI)	39–45	46–52	≥53	Yes
Spielberger State Anxiety Scale (STAS)	36–49	50–59	≥60	Yes
DASS-21 Anxiety Subscale	8–9	10–14	≥15	Yes
SCL-ANX4 (CMD Screening Questionnaire)	~5–6	~7–9	≥10	No

Table. Cutoffs for baseline fatigue

Scale	Mild Fatigue Cutoff	Moderate Fatigue Cutoff	Severe Fatigue Cutoff	Cutoffs Published?
FACT-Fatigue	34–40	28–33	<28	Yes

**FACIT-Fatigue**	34–40	28–33	<28	Yes
**EORTC QLQ-C30 Fatigue (3-item)**	40-59	60-70	>70	Yes
**FACT-Anemia (20-item)**	33-42	25-32	<25	No
**PROMIS Fatigue (T-score)**	55–59	60–69	≥70	Yes
**Checklist Individual Strength-Fatigue**	35–39	40–44	≥45	Yes
**MDASI-Fatigue**	1–3	4–6	7–10	Yes
**Brief Fatigue Inventory**	1–3	4–6	7–10	Yes

Table. Categories of education levels reported in the included studies and their estimated numbers of years required

**Education category**	**Estimated number of years**
No education	0
Primary school	6
Secondary school	12
Higher education	16
University/college	16
Postgraduate	18
Less than a college degree	12
College degree or higher/Bachelors or higher/Higher education degree	17
High	16
Middle	12
Low	10
Middle school	8
Never finished secondary school	10
Graduate school	18
Vocational training/some college or less	14
Municipal primary and lower secondary school, incl. apprenticeships	10
Short	12
Medium	14
Long	16
High school education or more	14
Graduate/professional school	18
High school graduate/GED or less	11

Composite outcome data generation

Where two assessment tools were used within a study to measure the same cognitive domain (i.e., outcome), for example, learning measured with the HVLT-R total recall and the Brief Visuospatial Memory Test (BVMT) total recall, to maximize data inclusion and avoid statistical dependencies, we used a reductionist approach[8] and aggregated outcome

data following established methods[9,10]. First, the raw scores to be aggregated were converted to t-scores using normative data obtained from publisher test manuals, when available, or by using other sources (e.g., validation studies looking to generate normative data for the cognitive test of interest, specific to the country or population of interest). Where possible, we used normative data for the appropriate age, sex, and education level of study participants. Composite sample sizes and means were calculated as the average sample size and mean score of the two tests, while composite score standard deviations were calculated using the formula

$$\sqrt{\frac{1}{4} * (sd_1{}^2 + sd_2{}^2 + 2 * \rho * sd_1 * sd_2)},$$

where the estimated correlation between the two tests, ϱ, was assumed to be 0.7[11].

File S3: Literature search methods and strategies

Original search strategies

6 Aug 2023

Ovid Multifile

Database: Embase Classic+Embase, Ovid MEDLINE(R) ALL, APA PsycInfo, EBM Reviews - Cochrane Central Register of Controlled Trials

Search Strategy:
----
1 Chemotherapy-Related Cognitive Impairment/ (187)
2 (chemofog* or chemobrain* or chemo-fog* or chemo-brain*).tw,kw,kf. (1245)
3 (cancer adj3 fog).tw,kw,kf. (19)
4 (CTRCI adj10 (chemother* or chemo-ther* or cognit* or impair*)).tw,kw,kf. (604)
5 (CRND adj10 (cancer* or neurocognit* or neuro-cognit* or dysfunction*)).tw,kw,kf. (12)
6 (CICI adj10 (chemother* or chemo-ther* or cognit* or impair*)).tw,kw,kf. (174)
7 ((chemotherapy-induced or chemo-therapy-induced or chemotherapy-related or chemo-therapy related or chemo-induced or chemo-related) adj5 ((cognit* or neurocogniti* or neuro-cogniti* or neuropsycholog* or neuro-psycholog* or memory or memories or neurobehavio?r* or neuro-behavio?r* or (problem? adj1 solv*) or attention or concentrat*) adj5 (deficit* or declin* or disorder* or dysfunction* or impair* or decrement* or disturb* or problem* or sequela*))).tw,kw,kf. (1126)
8 or/1-7 [CTRCI/CRND/CICI - Pt 1] (2398)
9 exp Neoplasms/ (9997204)
10 (neoplas* or cancer* or tumour* or tumor* or carcinoma* or malignan* or metasta* or oncolog*).tw,kw,kf. (10283980)
11 (adenoma? or adenocarcinoma? or adeno-carcinoma? or blastoma? or carcinosarcoma? or carcino-sarcoma? or hepatoblastoma? or hepato-blastoma? or leukemia? or leukaemia? or lymphoma? or melanoma? or mesenchymoma? or mesothelioma? or sarcoma? or thymoma?).tw,kw,kf. (2427353)
12 or/9-11 [CANCER] (13015726)
13 exp Neoplasms/dt [drug therapy] (1508898)
14 exp Antineoplastic Agents/ (4311354)
15 (antineoplastic* or anti-neoplastic* or chemotherap* or chemo-therap*).tw,kw,kf. (1452250)
16 ((anticancer* or anti-cancer* or antitumo?r* or anti-tumo?r*) adj3 (agent? or drug? or medication? or pharmaceutic* or pharma-ceutic*)).tw,kw,kf. (229253)
17 (systemic adj2 (therap* or treatment*)).tw,kw,kf. (157355)
18 (targeted adj2 (therap* or treatment*)).tw,kw,kf. (264041)
19 ((hormone or hormonal or endocrine) adj2 (agent? or therap* or treatment*)).tw,kw,kf. (208953)
20 Survivorship/ (9120)
21 Cancer Survivors/ (39216)
22 survivor*.tw,kw,kf. (376485)

23 or/13-22 [SYSTEMIC TREATMENTS, INCL CANCER SURVIVORSHIP] (5980306)
24 12 and 23 [CANCER - SYSTEMIC TREATMENTS] (3630342)
25 Cognition Disorders/ (174812)
26 Cognitive Dysfunction/ (224877)
27 Cognition/de [drug effects] (13568)
28 Auditory Perceptual Disorders/ (24105)
29 Brain/de [drug effects] (68968)
30 ((brain or consciousness or mental or mind) adj3 (fog* or cloud* or fuzz*)).tw,kw,kf. (3039)
31 Attention/ (280283)
32 exp Neurobehavioral Manifestations/ (844207)
33 ((cognit* or neurocogniti* or neuro-cogniti* or neuropsycholog* or neuro-psycholog* or memory or memories or neurobehavio?r* or neuro-behavio?r* or (problem? adj1 solv*) or attention or concentrat*) adj5 (deficit* or declin* or disorder* or dysfunction* or impair* or decrement* or disturb* or problem* or sequela*)).tw,kw,kf. (1144330)
34 exp Mental Processes/ (6008088)
35 mental process*.tw,kw,kf. (11602)
36 (conceptual adj (function? or process* or think*)).tw,kw,kf. (2642)
37 (information process* adj2 human?).tw,kw,kf. (4541)
38 Executive Function/ (91717)
39 executive function*.tw,kw,kf. (131736)
40 exp Learning/ (1392408)
41 learning.tw,kw,kf. (1592141)
42 exp Memory/ (674142)
43 (memory or memories).tw,kw,kf. (1027836)
44 (mental* adj (recall* or recogni* or retention* or retain*)).tw,kw,kf. (418)
45 Neuropsychological Tests/ (113193)
46 Trail Making Test/ (6820)
47 (Hopkins Verbal Learning Test Revised or HVLT-R).tw,kw,kf. (1439)
48 (Trail Making Test or TMT).tw,kw,kf. (26766)
49 (Controlled Oral Word Association Test or COWAT).tw,kw,kf. (2041)
50 (California Verbal Learning Test or CVLT or CVLT-II).tw,kw,kf. (5253)
51 (Rey Auditory Verbal Learning Test or RAVT).tw,kw,kf. (4067)
52 (Brief Visuospatial Memory Revised or BVMT-R).tw,kw,kf. (737)
53 (Delis-Kaplan Executive Function or D-KEFS).tw,kw,kf. (1676)
54 ((Wechsler Adult Intelligence Scale or WAIS-IV) and coding).tw,kw,kf. (421)
55 (Symbol Digit Modalities Test or SDMT).tw,kw,kf. (5805)
56 or/25-55 [COGNITION / PROCESSES / TESTS] (8872679)
57 24 and 56 [CANCER - SYSTEMIC TREATMENTS - COGNITION / PROCESSES / TESTS - CTRCI/CRND/CICI - Pt 2] (192750)
58 8 or 57 [CTRCI/CRND/CICI - PARTS 1-2] (193156)
59 (exp Child/ or exp Infant/) not (exp Adult/ or Adolescent/) (3623433)
60 58 not 59 [CHILD-, INFANT-ONLY REMOVED] (184959)
61 (controlled clinical trial or randomized controlled trial or pragmatic clinical trial or equivalence trial).pt. (689424)

62 "Clinical Trials as Topic"/ (335087)
63 exp "Controlled Clinical Trials as Topic"/ (482198)
64 (randomi#ed or randomi#ation? or randomly or RCT or placebo*).tw,kw,kf. (4479528)
65 ((singl* or doubl* or trebl* or tripl*) adj (mask* or blind* or dumm*)).tw,kw,kf. (863266)
66 trial.ti. (1143316)
67 or/61-66 (5360708)
68 60 and 67 [RCTs] (25739)
69 exp Animals/ not Humans/ (17983438)
70 68 not 69 [ANIMAL-ONLY REMOVED] (18756)
71 (editorial or news or newspaper item).pt. (1658488)
72 (letter not (letter and randomized controlled trial)).pt. (2517667)
73 70 not (71 or 72) [OPINION PIECES REMOVED] (18404)
74 limit 73 to yr="1980-current" (18356)
75 74 use medall [MEDLINE RECORDS] (2483)
76 chemotherapy-related cognitive impairment/ (187)
77 (chemofog* or chemobrain* or chemo-fog* or chemo-brain*).ti,kw,kf. (781)
78 (cancer adj3 fog).ti,kw,kf. (12)
79 (CTRCI adj10 (chemother* or chemo-ther* or cognit* or impair*)).ti,kw,kf. (80)
80 (CRND adj10 (cancer* or neurocognit* or neuro-cognit* or dysfunction*)).ti,kw,kf. (0)
81 (CICI adj10 (chemother* or chemo-ther* or cognit* or impair*)).ti,kw,kf. (21)
82 ((chemotherapy-induced or chemo-therapy-induced or chemotherapy-related or chemo-therapy related or chemo-induced or chemo-related) adj5 ((cognit* or neurocogniti* or neuro-cogniti* or neuropsycholog* or neuro-psycholog* or memory or memories or neurobehavio?r* or neuro-behavio?r* or (problem? adj1 solv*) or attention or concentrat*) adj5 (deficit* or declin* or disorder* or dysfunction* or impair* or decrement* or disturb* or problem* or sequela*))).ti,kw,kf. (546)
83 or/76-82 [CTRCI/CRND/CICI - Pt 1] (1307)
84 exp neoplasm/ (9997204)
85 (neoplas* or cancer* or tumour* or tumor* or carcinoma* or malignan* or metasta* or oncolog*).ti,kw,kf. (6714089)
86 (adenoma? or adenocarcinoma? or adeno-carcinoma? or blastoma? or carcinosarcoma? or carcino-sarcoma? or hepatoblastoma? or hepato-blastoma? or leukemia? or leukaemia? or lymphoma? or melanoma? or mesenchymoma? or mesothelioma? or sarcoma? or thymoma?).ti,kw,kf. (1441608)
87 or/84-86 [CANCER] (11291187)
88 exp neoplasm/dt [drug therapy] (1508898)
89 exp antineoplastic agent/ (4311354)
90 (antineoplastic* or anti-neoplastic* or chemotherap* or chemo-therap*).ti,kw,kf. (492672)
91 ((anticancer* or anti-cancer* or antitumo?r* or anti-tumo?r*) adj3 (agent? or drug? or medication? or pharmaceutic* or pharma-ceutic*)).ti,kw,kf. (58817)
92 systemic therapy/ (82065)
93 (systemic adj2 (therap* or treatment*)).ti,kw,kf. (29803)
94 (targeted adj2 (therap* or treatment*)).ti,kw,kf. (66272)
95 cancer hormone therapy/ (26631)
96 ((hormone or hormonal or endocrine) adj2 (agent? or therap* or treatment*)).ti,kw,kf. (75048)
97 survivorship/ (9120)

98 cancer survivor/ (42673)
99 survivor*.ti,kw,kf. (123432)
100 or/88-99 [SYSTEMIC TREATMENTS, INCL CANCER SURVIVORSHIP] (5077470)
101 87 and 100 [CANCER - SYSTEMIC TREATMENTS] (2858601)
102 cognitive defect/ (215851)
103 "clouding of consciousness"/ (684)
104 ((brain or consciousness or mental or mind) adj3 (fog* or cloud* or fuzz*)).ti,kw,kf. (596)
105 attention/ (280283)
106 ((cognit* or neurocogniti* or neuro-cogniti* or neuropsycholog* or neuro-psycholog* or memory or memories or neurobehavio?r* or neuro-behavio?r* or (problem? adj1 solv*) or attention or concentrat*) adj5 (deficit* or declin* or disorder* or dysfunction* or impair* or decrement* or disturb* or problem* or sequela*)).ti,kw,kf. (344499)
107 exp mental function/ (4734132)
108 mental process*.ti,kw,kf. (1405)
109 (conceptual adj (function? or process* or think*)).ti,kw,kf. (475)
110 (information process* adj2 human?).ti,kw,kf. (2800)
111 executive function/ (91717)
112 executive function test/ (1527)
113 executive function*.ti,kw,kf. (43337)
114 exp learning/ (1392408)
115 learning.ti,kw,kf. (675306)
116 exp memory/ (674142)
117 exp verbal memory test/ (7302)
118 (memory or memories).ti,kw,kf. (395721)
119 (mental* adj (recall* or recogni* or retention* or retain*)).ti,kw,kf. (316)
120 neuropsychological test/ (174830)
121 trail making test/ (6820)
122 exp verbal memory test/ (7302)
123 (Hopkins Verbal Learning Test Revised or HVLT-R).ti,kw,kf. (126)
124 (Trail Making Test or TMT).ti,kw,kf. (3968)
125 (Controlled Oral Word Association Test or COWAT).ti,kw,kf. (188)
126 (California Verbal Learning Test or CVLT or CVLT-II).ti,kw,kf. (804)
127 (Rey Auditory Verbal Learning Test or RAVT).ti,kw,kf. (517)
128 (Brief Visuospatial Memory Revised or BVMT-R).ti,kw,kf. (43)
129 Delis-Kaplan executive function system/ (104073)
130 (Delis-Kaplan Executive Function or D-KEFS).ti,kw,kf. (289)
131 Wechsler adult intelligence scale/ (12403)
132 ((Wechsler Adult Intelligence Scale or WAIS-IV) and coding).ti,kw,kf. (6)
133 symbol digit modalities test/ (105904)
134 (Symbol Digit Modalities Test or SDMT).ti,kw,kf. (692)
135 or/102-134 [COGNITION / PROCESSES / TESTS] (6474248)
136 101 and 135 [CANCER - SYSTEMIC TREATMENTS - COGNITION / PROCESSES / TESTS - CTRCI/CRND/CICI - Pt 2] (119631)
137 83 or 136 [CTRCI/CRND/CICI - PARTS 1-2] (120226)

138 (exp child/ or exp adolescent/) not exp adult/ (4533657)
139 137 not 138 [CHILD-, INFANT-ONLY REMOVED] (113409)
140 exp randomized controlled trial/ or controlled clinical trial/ (1703979)
141 clinical trial/ (1658296)
142 exp "controlled clinical trial (topic)"/ (268199)
143 (randomi#ed or randomi#ation? or randomly or RCT or placebo*).ti,kw,kf. (1546692)
144 ((singl* or doubl* or trebl* or tripl*) adj (mask* or blind* or dumm*)).ti,kw,kf. (329997)
145 trial.ti. (1143316)
146 or/140-145 (4086878)
147 139 and 146 [RCTs] (21732)
148 (exp animal/ or exp animal experimentation/ or exp animal model/ or exp animal experiment/ or nonhuman/ or exp vertebrate/) not (exp human/ or exp human experimentation/ or exp human experiment/) (13181979)
149 147 not 148 [ANIMAL-ONLY REMOVED] (21675)
150 editorial.pt. (1438305)
151 letter.pt. not (letter.pt. and randomized controlled trial/) (2511284)
152 149 not (150 or 151) [OPINION PIECES REMOVED] (20979)
153 conference abstract.pt. (4847411)
154 152 not 153 [CONFERENCE ABSTRACTS REMOVED] (14231)
155 limit 154 to yr="1980-current" (14202)
156 155 use emczd [EMBASE RECORDS] (13347)
157 (chemofog* or chemobrain* or chemo-fog* or chemo-brain*).tw,id. (1039)
158 (cancer adj3 fog).tw,id. (17)
159 (CTRCI adj10 (chemother* or chemo-ther* or cognit* or impair*)).tw,id. (582)
160 (CRND adj10 (cancer* or neurocognit* or neuro-cognit* or dysfunction*)).tw,id. (12)
161 (CICI adj10 (chemother* or chemo-ther* or cognit* or impair*)).tw,id. (167)
162 ((chemotherapy-induced or chemo-therapy-induced or chemotherapy-related or chemo-therapy related or chemo-induced or chemo-related) adj5 ((cognit* or neurocogniti* or neuro-cogniti* or neuropsycholog* or neuropsycholog* or memory or memories or neurobehavio?r* or neuro-behavio?r* or (problem? adj1 solv*) or attention or concentrat*) adj5 (deficit* or declin* or disorder* or dysfunction* or impair* or decrement* or disturb* or problem* or sequela*))).tw,id. (1090)
163 or/157-162 [CTRCI/CRND/CICI - Pt 1] (2219)
164 exp Neoplasms/ (9997204)
165 (neoplas* or cancer* or tumour* or tumor* or carcinoma* or malignan* or metasta* or oncolog*).tw,id. (10104964)
166 (adenoma? or adenocarcinoma? or adeno-carcinoma? or blastoma? or carcinosarcoma? or carcino-sarcoma? or hepatoblastoma? or hepato-blastoma? or leukemia? or leukaemia? or lymphoma? or melanoma? or mesenchymoma? or mesothelioma? or sarcoma? or thymoma?).tw,id. (2375345)
167 or/164-166 [CANCER] (12957318)
168 Antineoplastic Drugs/ (328576)
169 Chemotherapy/ (217401)
170 (antineoplastic* or anti-neoplastic* or chemotherap* or chemo-therap*).tw,id. (1411899)
171 ((anticancer* or anti-cancer* or antitumo?r* or anti-tumo?r*) adj3 (agent? or drug? or medication? or pharmaceutic* or pharma-ceutic*)).tw,id. (222075)
172 (systemic adj2 (therap* or treatment*)).tw,id. (155272)

173 (targeted adj2 (therap* or treatment*)).tw,id. (246965)
174 ((hormone or hormonal or endocrine) adj2 (agent? or therap* or treatment*)).tw,id. (202797)
175 Survivors/ (109776)
176 survivor*.tw,id. (372306)
177 or/168-176 [SYSTEMIC TREATMENTS, INCL CANCER SURVIVORSHIP] (2638825)
178 167 and 177 [CANCER - SYSTEMIC TREATMENTS] (1966456)
179 Cognitive Impairment/ (295164)
180 ((brain or consciousness or mental or mind) adj3 (fog* or cloud* or fuzz*)).tw,id. (2941)
181 exp Attention/ (521245)
182 ((cognit* or neurocogniti* or neuro-cogniti* or neuropsycholog* or neuro-psycholog* or memory or memories or neurobehavio?r* or neuro-behavio?r* or (problem? adj1 solv*) or attention or concentrat*) adj5 (deficit* or declin* or disorder* or dysfunction* or impair* or decrement* or disturb* or problem* or sequela*)).tw,id. (1126506)
183 exp Cognitive Processes/ (934247)
184 mental process*.tw,id. (11068)
185 (conceptual adj (function? or process* or think*)).tw,id. (2606)
186 (information process* adj2 human?).tw,id. (2157)
187 Executive Function/ (91717)
188 executive function*.tw,id. (124871)
189 exp Learning/ (1392408)
190 learning.tw,id. (1532946)
191 Memory Disorders/ (54333)
192 exp Memory/ (674142)
193 (memory or memories).tw,id. (1008871)
194 (mental* adj (recall* or recogni* or retention* or retain*)).tw,id. (109)
195 Neuropsychological Assessment/ (20472)
196 Cognitive Assessment/ (9695)
197 Cognitive Measures/ (78)
198 Executive Functioning Measures/ (35)
199 "Memory and Learning Measures"/ (142)
200 (Hopkins Verbal Learning Test Revised or HVLT-R).tw,id. (1431)
201 (Trail Making Test or TMT).tw,id. (26136)
202 (Controlled Oral Word Association Test or COWAT).tw,id. (1982)
203 (California Verbal Learning Test or CVLT or CVLT-II).tw,id. (5208)
204 (Rey Auditory Verbal Learning Test or RAVT).tw,id. (3959)
205 (Brief Visuospatial Memory Revised or BVMT-R).tw,id. (734)
206 (Delis-Kaplan Executive Function or D-KEFS).tw,id. (1642)
207 Wechsler Adult Intelligence Scale/ (12403)
208 ((Wechsler Adult Intelligence Scale or WAIS-IV) and coding).tw,id. (413)
209 (Symbol Digit Modalities Test or SDMT).tw,id. (5759)
210 or/179-209 [COGNITION / PROCESSES / TESTS] (4848785)
211 178 and 210 [CANCER - SYSTEMIC TREATMENTS - COGNITION / PROCESSES / TESTS - CTRCI/CRND/CICI - Pt 2] (45056)
212 163 or 211 [CTRCI/CRND/CICI - PARTS 1-2] (45496)

213 exp Randomized Controlled Trials/ (468257)
214 Clinical Trials/ (125218)
215 Placebo/ (417700)
216 ((singl* or doubl* or trebl* or tripl*) adj (mask* or blind* or dumm*)).tw,id. (834059)
217 (randomi#ed or randomi#ation? or randomly or RCT or placebo*).tw,id. (4414622)
218 trial.ti. (1143316)
219 or/213-218 (5119430)
220 212 and 219 [RCTs] (5822)
221 limit 220 to yr="1980-current" (5815)
222 221 use medall,emczd,cctr (5536)
223 221 not 222 [PSYCINFO RECORDS] (279)
224 Chemotherapy-Related Cognitive Impairment/ (187)
225 (chemofog* or chemobrain* or chemo-fog* or chemo-brain*).ti,ab,kw. (1214)
226 (cancer adj3 fog).ti,ab,kw. (16)
227 (CTRCI adj10 (chemother* or chemo-ther* or cognit* or impair*)).ti,ab,kw. (583)
228 (CRND adj10 (cancer* or neurocognit* or neuro-cognit* or dysfunction*)).ti,ab,kw. (12)
229 (CICI adj10 (chemother* or chemo-ther* or cognit* or impair*)).ti,ab,kw. (170)
230 ((chemotherapy-induced or chemo-therapy-induced or chemotherapy-related or chemo-therapy related or chemo-induced or chemo-related) adj5 ((cognit* or neurocogniti* or neuro-cogniti* or neuropsycholog* or neuro-psycholog* or memory or memories or neurobehavio?r* or neuro-behavio?r* or (problem? adj1 solv*) or attention or concentrat*) adj5 (deficit* or declin* or disorder* or dysfunction* or impair* or decrement* or disturb* or problem* or sequela*))).ti,ab,kw. (1086)
231 or/224-230 [CTRCI/CRND/CICI - Pt 1] (2344)
232 exp Neoplasms/ (9997204)
233 (neoplas* or cancer* or tumour* or tumor* or carcinoma* or malignan* or metasta* or oncolog*).ti,ab,kw. (10177786)
234 (adenoma? or adenocarcinoma? or adeno-carcinoma? or blastoma? or carcinosarcoma? or carcino-sarcoma? or hepatoblastoma? or hepato-blastoma? or leukemia? or leukaemia? or lymphoma? or melanoma? or mesenchymoma? or mesothelioma? or sarcoma? or thymoma?).ti,ab,kw. (2398392)
235 or/232-234 [CANCER] (12989419)
236 exp Neoplasms/dt [drug therapy] (1508898)
237 exp Antineoplastic Agents/ (4311354)
238 (antineoplastic* or anti-neoplastic* or chemotherap* or chemo-therap*).ti,ab,kw. (1445744)
239 ((anticancer* or anti-cancer* or antitumo?r* or anti-tumo?r*) adj3 (agent? or drug? or medication? or pharmaceutic* or pharma-ceutic*)).ti,ab,kw. (222192)
240 (systemic adj2 (therap* or treatment*)).ti,ab,kw. (155529)
241 (targeted adj2 (therap* or treatment*)).ti,ab,kw. (247113)
242 ((hormone or hormonal or endocrine) adj2 (agent? or therap* or treatment*)).ti,ab,kw. (203906)
243 Survivorship/ (9120)
244 Cancer Survivors/ (39216)
245 survivor*.ti,ab,kw. (373195)
246 or/236-245 [SYSTEMIC TREATMENTS, INCL CANCER SURVIVORSHIP] (5963993)
247 235 and 246 [CANCER - SYSTEMIC TREATMENTS] (3617863)
248 Cognition Disorders/ (174812)

249 Cognitive Dysfunction/ (224877)
250 Cognition/de [drug effects] (13568)
251 Auditory Perceptual Disorders/ (24105)
252 Brain/de [drug effects] (68968)
253 ((brain or consciousness or mental or mind) adj3 (fog* or cloud* or fuzz*)).ti,ab,kw. (2931)
254 Attention/ (280283)
255 exp Neurobehavioral Manifestations/ (844207)
256 ((cognit* or neurocogniti* or neuro-cogniti* or neuropsycholog* or neuro-psycholog* or memory or memories or neurobehavio?r* or neuro-behavio?r* or (problem? adj1 solv*) or attention or concentrat*) adj5 (deficit* or declin* or disorder* or dysfunction* or impair* or decrement* or disturb* or problem* or sequela*)).ti,ab,kw. (1114063)
257 exp Mental Processes/ (6008088)
258 mental process*.ti,ab,kw. (11240)
259 (conceptual adj (function? or process* or think*)).ti,ab,kw. (2533)
260 (information process* adj2 human?).ti,ab,kw. (4366)
261 Executive Function/ (91717)
262 executive function*.ti,ab,kw. (128930)
263 exp Learning/ (1392408)
264 learning.ti,ab,kw. (1508676)
265 exp Memory/ (674142)
266 (memory or memories).ti,ab,kw. (1002544)
267 (mental* adj (recall* or recogni* or retention* or retain*)).ti,ab,kw. (309)
268 Neuropsychological Tests/ (113193)
269 Trail Making Test/ (6820)
270 (Hopkins Verbal Learning Test Revised or HVLT-R).ti,ab,kw. (1434)
271 (Trail Making Test or TMT).ti,ab,kw. (26492)
272 (Controlled Oral Word Association Test or COWAT).ti,ab,kw. (2031)
273 (California Verbal Learning Test or CVLT or CVLT-II).ti,ab,kw. (5233)
274 (Rey Auditory Verbal Learning Test or RAVT).ti,ab,kw. (4042)
275 (Brief Visuospatial Memory Revised or BVMT-R).ti,ab,kw. (735)
276 (Delis-Kaplan Executive Function or D-KEFS).ti,ab,kw. (1662)
277 ((Wechsler Adult Intelligence Scale or WAIS-IV) and coding).ti,ab,kw. (403)
278 (Symbol Digit Modalities Test or SDMT).ti,ab,kw. (5794)
279 or/248-278 [COGNITION / PROCESSES / TESTS] (8799035)
280 247 and 279 [CANCER - SYSTEMIC TREATMENTS - COGNITION / PROCESSES / TESTS - CTRCI/CRND/CICI - Pt 2] (191375)
281 231 or 280 [CTRCI/CRND/CICI - PARTS 1-2] (191784)
282 (exp Child/ or exp Infant/) not (exp Adult/ or Adolescent/) (3623433)
283 281 not 282 [CHILD-, INFANT-ONLY REMOVED] (183613)
284 limit 283 to yr="1980-current" (181916)
285 284 use cctr [CENTRAL RECORDS] (2522)
286 75 or 156 or 223 or 286 [ALL DATABASES] (18631)
287 limit 286 to yr="2018-current" (5694)
288 remove duplicates from 287 (4913)

289 limit 286 to yr="2010-2017" (5562)
290 remove duplicates from 289 (4891)
291 limit 286 to yr="2003-2009" (5700)
292 remove duplicates from 291 (5353)
293 286 not (287 or 289 or 291) (1675)
294 remove duplicates from 293 (1470)
295 288 or 290 or 292 or 294 [TOTAL UNIQUE RECORDS] (16627)
296 295 use medall [MEDLINE UNIQUE RECORDS] (2476)
297 295 use emczd [EMBASE UNIQUE RECORDS] (12597)
298 295 use psyh [PSYCINFO UNIQUE RECORDS] (98)
299 295 use cctr [CENTRAL UNIQUE RECORDS] (1456)

****

CINAHL

#	Query	Limiters/Expanders	Last Run Via	Results
S69	S67 AND S68	Expanders - Apply related words; Apply equivalent subjects<br>Search modes - Boolean/Phrase	Interface - EBSCOhost Research Databases<br>Search Screen - Advanced Search<br>Database - CINAHL	1,406
S68	DT 1980-3000	Expanders - Apply related words; Apply equivalent subjects<br>Search modes - Boolean/Phrase	Interface - EBSCOhost Research Databases<br>Search Screen - Advanced Search<br>Database - CINAHL	8,473,219
S67	S65 NOT S66	Expanders - Apply related words; Apply equivalent subjects<br>Search modes - Boolean/Phrase	Interface - EBSCOhost Research Databases<br>Search Screen -	1,406

			Advanced Search Database - CINAHL	
S66	PT editorial	Expanders - Apply related words; Apply equivalent subjects<br>Search modes - Boolean/Phrase	Interface - EBSCOhost Research Databases Search Screen - Advanced Search Database - CINAHL	333,550
S65	S57 AND S64	Expanders - Apply related words; Apply equivalent subjects<br>Search modes - Boolean/Phrase	Interface - EBSCOhost Research Databases Search Screen - Advanced Search Database - CINAHL	1,415
S64	S58 OR S59 OR S60 OR S61 OR S62 OR S63	Expanders - Apply related words; Apply equivalent subjects<br>Search modes - Boolean/Phrase	Interface - EBSCOhost Research Databases Search Screen - Advanced Search Database - CINAHL	587,333
S63	TI trial	Expanders - Apply related words; Apply equivalent subjects<br>Search modes - Boolean/Phrase	Interface - EBSCOhost Research Databases Search Screen - Advanced Search	182,235

			Database - CINAHL	
S62	TI ((singl* or doubl* or trebl* or tripl*) W0 (mask* or blind* or dumm*)) OR AB ((singl* or doubl* or trebl* or tripl*) W0 (mask* or blind* or dumm*))	Expanders - Apply related words; Apply equivalent subjects<br>Search modes - Boolean/Phrase	Interface - EBSCOhost Research Databases Search Screen - Advanced Search Database - CINAHL	58,721
S61	TI (randomi?ed or randomi?ation# or randomly or RCT or placebo*) OR AB (randomi?ed or randomi?ation# or randomly or RCT or placebo*)	Expanders - Apply related words; Apply equivalent subjects<br>Search modes - Boolean/Phrase	Interface - EBSCOhost Research Databases Search Screen - Advanced Search Database - CINAHL	408,224
S60	(MH "Randomized Controlled Trials+")	Expanders - Apply related words; Apply equivalent subjects<br>Search modes - Boolean/Phrase	Interface - EBSCOhost Research Databases Search Screen - Advanced Search Database - CINAHL	137,500
S59	(MH "Double-Blind Studies") OR (MH "Single-Blind Studies") OR (MH "Triple-Blind Studies")	Expanders - Apply related words; Apply equivalent subjects<br>Search modes - Boolean/Phrase	Interface - EBSCOhost Research Databases Search Screen - Advanced Search Database - CINAHL	69,976

S58	(MH "Clinical Trials")	Expanders - Apply related words; Apply equivalent subjects<br>Search modes - Boolean/Phrase	Interface - EBSCOhost Research Databases<br>Search Screen - Advanced Search<br>Database - CINAHL	186,355
S57	S8 OR S56	Expanders - Apply related words; Apply equivalent subjects<br>Search modes - Boolean/Phrase	Interface - EBSCOhost Research Databases<br>Search Screen - Advanced Search<br>Database - CINAHL	9,343
S56	S24 AND S55	Expanders - Apply related words; Apply equivalent subjects<br>Search modes - Boolean/Phrase	Interface - EBSCOhost Research Databases<br>Search Screen - Advanced Search<br>Database - CINAHL	9,220
S55	S25 OR S26 OR S27 OR S28 OR S29 OR S30 OR S31 OR S32 OR S33 OR S34 OR S35 OR S36 OR S37 OR S38 OR S39 OR S40 OR S41 OR S42 OR S43 OR S44 OR S45 OR S46 OR S47 OR S48 OR S49 OR S50 OR S51 OR S52 OR S53 OR S54	Expanders - Apply related words; Apply equivalent subjects<br>Search modes - Boolean/Phrase	Interface - EBSCOhost Research Databases<br>Search Screen - Advanced Search<br>Database - CINAHL	761,351
S54	TI ("Symbol Digit Modalities Test" or SDMT) OR AB ("Symbol Digit Modalities Test" or SDMT)	Expanders - Apply related words;	Interface - EBSCOhost	336

		Apply equivalent subjects<br>Search modes - Boolean/Phrase	Research Databases<br>Search Screen - Advanced Search<br>Database - CINAHL	
S53	(MH "Wechsler Adult Intelligence Scale-Revised") OR (MH "Wechsler Memory Scale-Revised")	Expanders - Apply related words; Apply equivalent subjects<br>Search modes - Boolean/Phrase	Interface - EBSCOhost Research Databases<br>Search Screen - Advanced Search<br>Database - CINAHL	1,330
S52	TI ("Delis-Kaplan Executive Function" or "D-KEFS") OR AB ("Delis-Kaplan Executive Function" or "D-KEFS")	Expanders - Apply related words; Apply equivalent subjects<br>Search modes - Boolean/Phrase	Interface - EBSCOhost Research Databases<br>Search Screen - Advanced Search<br>Database - CINAHL	143
S51	TI ("Brief Visuospatial Memory Revised" or "BVMT-R") OR AB ("Brief Visuospatial Memory Revised" or "BVMT-R")	Expanders - Apply related words; Apply equivalent subjects<br>Search modes - Boolean/Phrase	Interface - EBSCOhost Research Databases<br>Search Screen - Advanced Search<br>Database - CINAHL	28
S50	TI ("Rey Auditory Verbal Learning Test" or RAVT) OR AB ("Rey Auditory Verbal Learning Test" or RAVT)	Expanders - Apply related words; Apply equivalent subjects	Interface - EBSCOhost Research Databases	348

		Search modes - Boolean/Phrase	Search Screen - Advanced Search Database - CINAHL	
S49	TI ("California Verbal Learning Test" or CVLT or "CVLT-II") OR AB ("California Verbal Learning Test" or CVLT or "CVLT-II")	Expanders - Apply related words; Apply equivalent subjects Search modes - Boolean/Phrase	Interface - EBSCOhost Research Databases Search Screen - Advanced Search Database - CINAHL	335
S48	TI ("Controlled Oral Word Association Test" or COWAT) OR AB ("Controlled Oral Word Association Test" or COWAT)	Expanders - Apply related words; Apply equivalent subjects Search modes - Boolean/Phrase	Interface - EBSCOhost Research Databases Search Screen - Advanced Search Database - CINAHL	138
S47	TI ("Hopkins Verbal Learning Test Revised" or "HVLT-R") OR AB ("Hopkins Verbal Learning Test Revised" or "HVLT-R")	Expanders - Apply related words; Apply equivalent subjects Search modes - Boolean/Phrase	Interface - EBSCOhost Research Databases Search Screen - Advanced Search Database - CINAHL	113
S46	TI ("Trail Making Test" or TMT) OR AB ("Trail Making Test" or TMT)	Expanders - Apply related words; Apply equivalent subjects Search modes - Boolean/Phrase	Interface - EBSCOhost Research Databases Search Screen -	1,932

			Advanced Search Database - CINAHL	
S45	(MH "Memory and Learning Tests")	Expanders - Apply related words; Apply equivalent subjects Search modes - Boolean/Phrase	Interface - EBSCOhost Research Databases Search Screen - Advanced Search Database - CINAHL	81
S44	(MH "Neuropsychological Tests")	Expanders - Apply related words; Apply equivalent subjects Search modes - Boolean/Phrase	Interface - EBSCOhost Research Databases Search Screen - Advanced Search Database - CINAHL	39,259
S43	TI (mental* W0 (recall* or recogni* or retention* or retain*)) OR AB (mental* W0 (recall* or recogni* or retention* or retain*))	Expanders - Apply related words; Apply equivalent subjects Search modes - Boolean/Phrase	Interface - EBSCOhost Research Databases Search Screen - Advanced Search Database - CINAHL	7
S42	TI (memory or memories) OR AB (memory or memories)	Expanders - Apply related words; Apply equivalent subjects Search modes - Boolean/Phrase	Interface - EBSCOhost Research Databases Search Screen - Advanced Search	60,214

			Database - CINAHL	
S41	(MH "Memory+")	Expanders - Apply related words; Apply equivalent subjects<br>Search modes - Boolean/Phrase	Interface - EBSCOhost Research Databases<br>Search Screen - Advanced Search<br>Database - CINAHL	37,102
S40	TI learning OR AB learning	Expanders - Apply related words; Apply equivalent subjects<br>Search modes - Boolean/Phrase	Interface - EBSCOhost Research Databases<br>Search Screen - Advanced Search<br>Database - CINAHL	154,188
S39	(MH "Learning+")	Expanders - Apply related words; Apply equivalent subjects<br>Search modes - Boolean/Phrase	Interface - EBSCOhost Research Databases<br>Search Screen - Advanced Search<br>Database - CINAHL	129,557
S38	TI executive W0 function* OR AB executive W0 function*	Expanders - Apply related words; Apply equivalent subjects<br>Search modes - Boolean/Phrase	Interface - EBSCOhost Research Databases<br>Search Screen - Advanced Search<br>Database - CINAHL	12,579

S37	(MH "Executive Function")	Expanders - Apply related words; Apply equivalent subjects<br>Search modes - Boolean/Phrase	Interface - EBSCOhost Research Databases<br>Search Screen - Advanced Search<br>Database - CINAHL	6,711
S36	TI (information W0 process*) N2 human# OR AB (information W0 process*) N2 human#	Expanders - Apply related words; Apply equivalent subjects<br>Search modes - Boolean/Phrase	Interface - EBSCOhost Research Databases<br>Search Screen - Advanced Search<br>Database - CINAHL	66
S35	TI (conceptual W0 (function# or process* or think*)) OR AB (conceptual W0 (function# or process* or think*))	Expanders - Apply related words; Apply equivalent subjects<br>Search modes - Boolean/Phrase	Interface - EBSCOhost Research Databases<br>Search Screen - Advanced Search<br>Database - CINAHL	150
S34	TI mental W0 process* OR AB mental W0 process*	Expanders - Apply related words; Apply equivalent subjects<br>Search modes - Boolean/Phrase	Interface - EBSCOhost Research Databases<br>Search Screen - Advanced Search<br>Database - CINAHL	406
S33	(MH "Mental Processes+")	Expanders - Apply related words;	Interface - EBSCOhost	452,456

		Apply equivalent subjects<br>Search modes - Boolean/Phrase	Research Databases<br>Search Screen - Advanced Search<br>Database - CINAHL	
S32	TI ((cognit* or neurocogniti* or neuro-cogniti* or neuropsycholog* or neuro-psycholog* or memory or memories or neurobehavio#r* or neuro-behavio#r* or (problem# N1 solv*) or attention or concentrat*) N5 (deficit* or declin* or disorder* or dysfunction* or impair* or decrement* or disturb* or problem* or sequela*)) OR AB ((cognit* or neurocogniti* or neuro-cogniti* or neuropsycholog* or neuro-psycholog* or memory or memories or neurobehavio#r* or neuro-behavio#r* or (problem# N1 solv*) or attention or concentrat*) N5 (deficit* or declin* or disorder* or dysfunction* or impair* or decrement* or disturb* or problem* or sequela*))	Expanders - Apply related words; Apply equivalent subjects<br>Search modes - Boolean/Phrase	Interface - EBSCOhost Research Databases<br>Search Screen - Advanced Search<br>Database - CINAHL	108,143
S31	(MH "Neurobehavioral Manifestations+")	Expanders - Apply related words; Apply equivalent subjects<br>Search modes - Boolean/Phrase	Interface - EBSCOhost Research Databases<br>Search Screen - Advanced Search<br>Database - CINAHL	110,056
S30	(MH "Attention")	Expanders - Apply related words; Apply equivalent subjects<br>Search modes - Boolean/Phrase	Interface - EBSCOhost Research Databases<br>Search Screen - Advanced Search<br>Database - CINAHL	18,460
S29	TI ((brain or consciousness or mental or mind) N3 (fog* or cloud* or fuzz*)) OR AB ((brain or consciousness or mental or mind) N3 (fog* or cloud* or fuzz*))	Expanders - Apply related words; Apply equivalent subjects	Interface - EBSCOhost Research Databases	252

		Search modes - Boolean/Phrase	Search Screen - Advanced Search Database - CINAHL	
S28	(MH "Brain/DE")	Expanders - Apply related words; Apply equivalent subjects Search modes - Boolean/Phrase	Interface - EBSCOhost Research Databases Search Screen - Advanced Search Database - CINAHL	5,082
S27	(MH "Auditory Perceptual Disorders")	Expanders - Apply related words; Apply equivalent subjects Search modes - Boolean/Phrase	Interface - EBSCOhost Research Databases Search Screen - Advanced Search Database - CINAHL	2,021
S26	(MH "Cognition/DE")	Expanders - Apply related words; Apply equivalent subjects Search modes - Boolean/Phrase	Interface - EBSCOhost Research Databases Search Screen - Advanced Search Database - CINAHL	3,560
S25	(MH "Cognition Disorders")	Expanders - Apply related words; Apply equivalent subjects Search modes - Boolean/Phrase	Interface - EBSCOhost Research Databases Search Screen -	34,389

			Advanced Search Database - CINAHL	
S24	S12 AND S23	Expanders - Apply related words; Apply equivalent subjects<br>Search modes - Boolean/Phrase	Interface - EBSCOhost Research Databases Search Screen - Advanced Search Database - CINAHL	208,151
S23	S13 OR S14 OR S15 OR S16 OR S17 OR S18 OR S19 OR S20 OR S21 OR S22	Expanders - Apply related words; Apply equivalent subjects<br>Search modes - Boolean/Phrase	Interface - EBSCOhost Research Databases Search Screen - Advanced Search Database - CINAHL	318,740
S22	TI survivor* OR AB survivor*	Expanders - Apply related words; Apply equivalent subjects<br>Search modes - Boolean/Phrase	Interface - EBSCOhost Research Databases Search Screen - Advanced Search Database - CINAHL	56,220
S21	(MH "Cancer Survivors")	Expanders - Apply related words; Apply equivalent subjects<br>Search modes - Boolean/Phrase	Interface - EBSCOhost Research Databases Search Screen - Advanced Search	13,076

			Database - CINAHL	
S20	(MH "Survivorship")	Expanders - Apply related words; Apply equivalent subjects<br>Search modes - Boolean/Phrase	Interface - EBSCOhost Research Databases<br>Search Screen - Advanced Search<br>Database - CINAHL	1,116
S19	TI ((hormone or hormonal or endocrine) N2 (agent# or therap* or treatment*)) OR AB ((hormone or hormonal or endocrine) N2 (agent# or therap* or treatment*))	Expanders - Apply related words; Apply equivalent subjects<br>Search modes - Boolean/Phrase	Interface - EBSCOhost Research Databases<br>Search Screen - Advanced Search<br>Database - CINAHL	17,599
S18	TI (targeted N2 (therap* or treatment*)) OR AB (targeted N2 (therap* or treatment*))	Expanders - Apply related words; Apply equivalent subjects<br>Search modes - Boolean/Phrase	Interface - EBSCOhost Research Databases<br>Search Screen - Advanced Search<br>Database - CINAHL	19,546
S17	TI (systemic N2 (therap* or treatment*)) OR AB (systemic N2 (therap* or treatment*))	Expanders - Apply related words; Apply equivalent subjects<br>Search modes - Boolean/Phrase	Interface - EBSCOhost Research Databases<br>Search Screen - Advanced Search<br>Database - CINAHL	13,708

S16	TI ((anticancer* or anti-cancer* or antitumo#r* or anti-tumo#r*) N3 (agent# or drug# or medication# or pharmaceutic* or pharma-ceutic*)) OR AB ((anticancer* or anti-cancer* or antitumo#r* or anti-tumo#r*) N3 (agent# or drug# or medication# or pharmaceutic* or pharma-ceutic*))	Expanders - Apply related words; Apply equivalent subjects<br>Search modes - Boolean/Phrase	Interface - EBSCOhost Research Databases<br>Search Screen - Advanced Search<br>Database - CINAHL	6,948
S15	TI (antineoplastic* or anti-neoplastic* or chemotherap* or chemo-therap*) OR AB (antineoplastic* or anti-neoplastic* or chemotherap* or chemo-therap*)	Expanders - Apply related words; Apply equivalent subjects<br>Search modes - Boolean/Phrase	Interface - EBSCOhost Research Databases<br>Search Screen - Advanced Search<br>Database - CINAHL	90,511
S14	(MH "Antineoplastic Agents+")	Expanders - Apply related words; Apply equivalent subjects<br>Search modes - Boolean/Phrase	Interface - EBSCOhost Research Databases<br>Search Screen - Advanced Search<br>Database - CINAHL	138,478
S13	(MH "Neoplasms+/DT")	Expanders - Apply related words; Apply equivalent subjects<br>Search modes - Boolean/Phrase	Interface - EBSCOhost Research Databases<br>Search Screen - Advanced Search<br>Database - CINAHL	120,579
S12	S9 OR S10 OR S11	Expanders - Apply related words;	Interface - EBSCOhost	805,199

		Apply equivalent subjects<br>Search modes - Boolean/Phrase	Research Databases<br>Search Screen - Advanced Search<br>Database - CINAHL	
S11	TI (adenoma# or adenocarcinoma# or adeno-carcinoma# or blastoma# or carcinosarcoma# or carcino-sarcoma# or hepatoblastoma# or hepato-blastoma# or leukemia# or leukaemia# or lymphoma# or melanoma# or mesenchymoma# or mesothelioma# or sarcoma# or thymoma#) OR AB (adenoma# or adenocarcinoma# or adeno-carcinoma# or blastoma# or carcinosarcoma# or carcino-sarcoma# or hepatoblastoma# or hepato-blastoma# or leukemia# or leukaemia# or lymphoma# or melanoma# or mesenchymoma# or mesothelioma# or sarcoma# or thymoma#)	Expanders - Apply related words; Apply equivalent subjects<br>Search modes - Boolean/Phrase	Interface - EBSCOhost Research Databases<br>Search Screen - Advanced Search<br>Database - CINAHL	122,317
S10	TI (neoplas* or cancer* or tumour* or tumor* or carcinoma* or malignan* or metasta* or oncolog*) OR AB (neoplas* or cancer* or tumour* or tumor* or carcinoma* or malignan* or metasta* or oncolog*)	Expanders - Apply related words; Apply equivalent subjects<br>Search modes - Boolean/Phrase	Interface - EBSCOhost Research Databases<br>Search Screen - Advanced Search<br>Database - CINAHL	737,183
S9	(MH "Neoplasms")	Expanders - Apply related words; Apply equivalent subjects<br>Search modes - Boolean/Phrase	Interface - EBSCOhost Research Databases<br>Search Screen - Advanced Search<br>Database - CINAHL	92,431
S8	S1 OR S2 OR S3 OR S4 OR S5 OR S6 OR S7	Expanders - Apply related words; Apply equivalent subjects	Interface - EBSCOhost Research Databases	343

		Search modes - Boolean/Phrase	Search Screen - Advanced Search Database - CINAHL	
S7	TI (("chemotherapy-induced" or "chemo-therapy-induced" or "chemotherapy-related" or "chemo-therapy related" or "chemo-induced" or "chemo-related") N5 ((cognit* or neurocogniti* or neuro-cogniti* or neuropsycholog* or neuro-psycholog* or memory or memories or neurobehavio#r* or neuro-behavio#r* or (problem# N1 solv*) or attention or concentrat*) N5 (deficit* or declin* or disorder* or dysfunction* or impair* or decrement* or disturb* or problem* or sequela*))) OR AB (("chemotherapy-induced" or "chemo-therapy-induced" or "chemotherapy-related" or "chemo-therapy related" or "chemo-induced" or "chemo-related") N5 ((cognit* or neurocogniti* or neuro-cogniti* or neuropsycholog* or neuro-psycholog* or memory or memories or neurobehavio#r* or neuro-behavio#r* or (problem# N1 solv*) or attention or concentrat*) N5 (deficit* or declin* or disorder* or dysfunction* or impair* or decrement* or disturb* or problem* or sequela*)))	Expanders - Apply related words; Apply equivalent subjects Search modes - Boolean/Phrase	Interface - EBSCOhost Research Databases Search Screen - Advanced Search Database - CINAHL	136
S6	TI (CICI N10 (chemother* or chemo-ther* or cognit* or impair*)) OR AB (CICI N10 (chemother* or chemo-ther* or cognit* or impair*))	Expanders - Apply related words; Apply equivalent subjects Search modes - Boolean/Phrase	Interface - EBSCOhost Research Databases Search Screen - Advanced Search Database - CINAHL	9
S5	TI (CRND N10 (cancer* or neurocognit* or neuro-cognit* or dysfunction*)) OR AB (CRND N10 (cancer* or neurocognit* or neuro-cognit* or dysfunction*))	Expanders - Apply related words; Apply equivalent subjects Search modes - Boolean/Phrase	Interface - EBSCOhost Research Databases Search Screen - Advanced Search Database - CINAHL	1
S4	TI (CTRCI N10 (chemother* or chemo-ther* or cognit* or impair*)) OR AB (CTRCI N10 (chemother* or chemo-ther* or cognit* or impair*))	Expanders - Apply related words; Apply equivalent subjects	Interface - EBSCOhost Research Databases	91

		Search modes - Boolean/Phrase	Search Screen - Advanced Search Database - CINAHL	
S3	TI cancer N3 fog OR AB cancer N3 fog	Expanders - Apply related words; Apply equivalent subjects Search modes - Boolean/Phrase	Interface - EBSCOhost Research Databases Search Screen - Advanced Search Database - CINAHL	4
S2	TI (chemofog* or chemobrain* or chemo-fog* or chemo-brain*) OR AB (chemofog* or chemobrain* or chemo-fog* or chemo-brain*)	Expanders - Apply related words; Apply equivalent subjects Search modes - Boolean/Phrase	Interface - EBSCOhost Research Databases Search Screen - Advanced Search Database - CINAHL	168
S1	(MH "Chemotherapy-Related Cognitive Impairment")	Expanders - Apply related words; Apply equivalent subjects Search modes - Boolean/Phrase	Interface - EBSCOhost Research Databases Search Screen - Advanced Search Database - CINAHL	21

Grey literature search methods

A search of the grey literature was undertaken for approximately 35 reviewer-hours, using the Grey Matters tool[12]. Between March 29 and April 8, 2025, we searched 83 websites that were listed under the following four categories: clinical trial registries, databases, health technology assessment (HTA) agencies, and Internet. Ten links did not work

(e.g., 'page not found') and 25 sites were not relevant, required a subscription, or were already searched as part of the main search (e.g. PubMed). We used search terms such as "cognitive function", "cognitive dysfunction", chemofog, and chemobrain. If search results were large, we added "AND cancer" to reduce the number of results. For feasibility, we filtered the results on the trial registries to those that were completed.

File S4: Detailed methods for CINeMA certainty of evidence appraisal

Certainty of the treatment-level effect estimates for each outcome were assessed using the Confidence in Network Meta-Analysis (CINeMA) framework (https://cinema.ispm.unibe.ch/#)[13,14]. For each outcome, within-study bias, reporting bias, indirectness, imprecision, heterogeneity, and incoherence domains were assessed to generate CINeMA certainty ratings ranging from high to moderate, low, and very low.

The following process was used for CINeMA assessment:

Within-study bias: The overall ROB assessment for each study was uploaded and we chose the "Average" ROB assessment for each treatment comparison to derive ratings for each comparison.

Reporting bias: We selected "Some concerns" of reporting bias for all treatment comparisons because (a) fewer than 10 studies were available for all treatment comparisons, precluding assessment of publication bias; (b) many of the included studies were designed as pilot/feasibility studies rather than to assess treatment efficacy; and (c) we identified registrations for completed clinical trials that did not have published findings.

Indirectness: For all outcomes, we assessed "Some concerns" of indirectness because our review question focused on adults of all ages and sexes, with any non-CNS cancer; however, the majority of studies included in NMAs enrolled predominantly women with an average age over 50 years with breast cancer. We also conducted a sensitivity analysis of the CINeMA assessment by assuming all treatment comparisons had Low risk of indirectness.

Imprecision, heterogeneity, and incoherence: We assessed imprecision, heterogeneity, and incoherence at a clinically important SMD of 0.50.

Final confidence rating: For final confidence ratings, due to commonalities between some CINeMA domains, concerns regarding both Imprecision and Heterogeneity were considered to be a single concern at the highest level assessed, and concerns regarding both Indirectness and Incoherence were considered to be a single concern at the highest level assessed. The following system was used to assess final confidence ratings:

- 1, 2, or 3 ratings of "Some concerns" = Moderate
- 1 "Major concerns" = Low
- 4 "Some concerns" = Low
- 1 "Some concerns" and 1 "Major concerns" = Low
- 2 "Major concerns" = Very low
- 1 "Some concerns" and 2 "Major concerns" = Very low
- 2 or more "Some concerns" and 1 "Major concerns" = Very low

File S5: Articles excluded during full-text screening

Conference abstract

Bellens, A., Sabbe, B., Van Dam, P.. A randomized study comparing two different training programs. Annals of oncology. 2018;29: https://doi.org/10.1093/annonc/mdy444.011.

Bray, V. J., Dhillon, H. M., Bell, M., Kabourakis, M., Rice, H., Yip, D., Boyle, F. M., Price, M., Vardy, J. L.. Evaluation of a web-based cognitive rehabilitation programme (CRP) in cancer survivors reporting cognitive symptoms following chemotherapy. Journal of clinical oncology. 2015;33(15).

Bray, V. J., Dhillon, H. M., Bell, M., Kabourakis, M., Rice, H., Yip, D., Boyle, F., Price, M., Vardy, J. L.. Evaluation of a web based cognitive rehabilitation program (CRP) in cancer survivors reporting cognitive symptoms following chemotherapy. Asia-Pacific journal of clinical oncology. 2015;11:47 https://doi.org/10.1111/ajco.12397.

Bray, V. J., Dhillon, H. M., Bell, M., Kabourakis, M., Rice, H., Yip, D., Boyle, F., Price, M., Vardy, J. L.. Evaluation of a web based cognitive rehabilitation programme (CRP) in cancer survivors reporting cognitive symptoms following chemotherapy. Asia-Pacific journal of clinical oncology. 2015;11:67 https://doi.org/10.1111/ajco.12432.

Campbell, K.. Effect of exercise on cancer-associated cognitive dysfunction: a proof-of-concept randomized controlled trial. Journal of clinical oncology. 2014;32(15).

Carlson, L., Tamagawa, R., Stephen, J., Doll, R., Speca, M.. Randomized-controlled multi-site trial of mindfulness-based cancer recovery (MBCR) versus supportive expressive group therapy (SET) among breast cancer survivors (mindset): long-term follow-up results. Psycho-oncology. 2013;22:103 https://doi.org/10.1111/j.1099-1611.2013.3393.

Cheng, Kk- F., Siah, R., Zhang, M., Keng, S. L., Wong, W. H., Ho, R., Lim, H. E.. Effect of an e-home based symptom management and mindfulness training program on quality of life in breast cancer survivors: a randomized clinical trial. Journal of clinical oncology. 2021;39(15 SUPPL): https://doi.org/10.1200/JCO.2021.39.15suppl.12080.

Derry, H. M., Bennett, J. M., Jaremka, L. M., Peng, J., Andridge, R. R., Shapiro, C., Malarkey, W. B., Emery, C. F., Layman, R., Mrozek, E., Kiecolt-Glaser, J. K.. Yoga and self-reported cognitive problems: a randomized controlled trial for breast cancer survivors. Psychosomatic medicine. 2014;76(3):A https://doi.org/10.1097/PSY.0000000000000057.

Dodds, S., Pace, T. W. W., Bell, M., Fiero, M., Negi, L. T., Raison, C., Weihs, K.. Feasibility and effects of Cognitively-Based Compassion Training (CBCT) on psychological well-being in breast cancer survivors: a randomized, waitlist controlled pilot study. Psycho-oncology. 2015;24:96 https://doi.org/10.1002/pon.3873.

Dos Santos, M., Rigal, O., Leger, I., Licaj, I., Dauchy, S., Levy, C., Noal, S., Segura, C., Delcambre, C., Allouache, D., Parzy, A., Lange, M., Capel, A., Grellard, J. M., Clarisse, B., Lefel, J., Joly, F.. Cognitive rehabilitation program to improve cognition of cancer patients treated with chemotherapy: a randomized controlled multicenter trial. Journal of clinical oncology. 2019;37: https://doi.org/10.1200/JCO.2019.37.15suppl.11521.

Ferguson, R. J., Terhorst, L., Terkpertey, E., Bailey, J. N., Impagliazzo, C., McDonald, B. C.. Telehealth cognitive-

behavioral therapy for cancer-related cognitive impairment: 2024 update of a model for remote clinical trial participation. Journal of clinical oncology. 2024;42(16).

Galiano-Castillo, N., Cantarero-Villanueva, I., Fern, aacute, ndez-Lao, C., iacute, az, Rodr, iacute, guez, L., Del, Moral, Aacute, vila, R., Arroyo-Morales, M.. Feasibility and preliminary results of a telerehabilitation system (e-cuidate) in breast cancer survivors about cognition and quality of life. Psycho-oncology. 2014;23:255 https://doi.org/10.1111/j.1099-1611.2014.3696.

Gates, P., Green, H. J., Gough, K., Dhillon, H. M., Vardy, J. L., Dickinson, M., Krishnasamy, M., Guarnera, J., Livingston, P. M., White, V. M., Ugalde, A., Caeyenberghs, K.. Web-based cognitive rehabilitation intervention for cancer-related cognitive impairment following chemotherapy for aggressive lymphoma: a randomised pilot trial...Cancer Nurses Society of Australia (CNSA), 26th Annual Congress, June 19-21, 2024, Brisbane, Queensland. Australian journal of cancer nursing. 2024;25(1):16.

Goedendorp, M., Knoop, H., Gielissen, M., Verhagen, S., Bleijenberg, G.. Cognitive behavior therapy for fatigue in cancer survivors: recent findings and developments. Psycho-oncology. 2013;22:88 https://doi.org/10.1111/j.1099-1611.2013.3393.

Haynes, S., Ryan, E., Wood, H., Fallon, J., Cook, E., Bolutayo, K., Li, Y., Hudis, C., Traina, T., Ahles, T.. Rehabilitation of cognitive changes in breast cancer survivors. Psycho-oncology. 2013;22:115 https://doi.org/10.10002/pon.3245.

Janelsins, M. C., Peppone, L. J., Heckler, C. E., Sprod, L., Mohile, S. G., Chandwani, K. D., Kirshner, J. J., Reddy, P. S., Morrow, G. R., Mustian, K. M.. YOCAS yoga, fatigue, memory difficulty, and quality of life: results from a URCC CCOP randomized, controlled clinical trial among 358 cancer survivors. Journal of clinical oncology. 2012;30(15).

Janelsins, M., Peppone, L., Heckler, C., Sprod, L., Mohile, S., Morrow, G., Mustian, K.. YOCAS©® Yoga: improved memory and memory-mediated improvements in fatigue and quality of life (QOL) in a nationwide phase III RCT. Supportive care in cancer. 2013;21:S280 https://doi.org/10.1007/s00520-013-1798-3.

Janelsins, Michelle Christine, Gada, Umang, Culakova, Eva, Tejani, Mohamedtaki Abdulaziz, Ahles, Tim, Bautista, Javier, O'Rourke, Mark Allen, Berenberg, Jeffrey L., Le-Lindqwister, Nguyet, McCormack, Steven Eric, Rasheed, Husain, Morrow, Gary R., Mustian, Karen Michelle, Peppone, Luke Joseph, Mohile, Supriya Gupta, Dunne, Richard Francis. Nationwide phase II randomized controlled trial of low-dose ibuprofen vs placebo for cancer-related cognitive impairment (CTRCI). Journal of Clinical Oncology. 2023;41:12116-12116 10.1200/JCO.2023.41.16_suppl.12116.

Johns, S., Talib, T., Von Ah, D., Monahan, P., Tong, Y., Giesler, B.. Mindfulness-based stress reduction for post-treatment survivors with cancer-related cognitive impairment: results of a randomized controlled pilot trial. Psycho-oncology. 2015;24:97 https://doi.org/10.1002/pon.3873.

Jung, M., Cha, K., Kim, M., Lee, J., Park, E., Cui, X., Dlamini, N.. Vr-based restorative activities improve cognitive function in women with cognitive complaints after chemotherapy for breast cancer. Supportive care in cancer. 2021;29(SUPPL 1):S66 https://doi.org/10.1007/s00520-021-06285-8.

Koevoets, E., Schagen, S. B., De Ruiter, M. B., Geerlings, M. I., Witlox, L., Van Der Wall, E., Stuiver, M. M., Sonke, G. S.,

Velthuis, M., Van Der Palen, J., Jobsen, J. J., May, A. M., Monninkhof, E. M.. Effect of physical exercise on cognitive function after chemotherapy in patients with breast cancer: a randomized controlled trial (PAM study). Journal of clinical oncology. 2021;39(15 SUPPL): https://doi.org/10.1200/JCO.2021.39.15suppl.12015.

Lawrence, J., Griffin, L., Rapp, S., Messino, M. J., Balcueva, E. P., Curtis, A. E., Groteluschen, D. L., Samuel, T. A., Lesser, G., Case, D., Shaw, E. G.. Quality of life and cognitive dysfunction in breast cancer survivors on a feasibility study of donepezil versus placebo. Journal of clinical oncology. 2014;32(15).

Lengacher, C., Hueluer, G., Reich, R., Rodriguez, C., Kip, K., Nguyen, A. T., Moscoso, M., Meng, H., Park, J., Chauca, K., Joshi, A., Wittenberg, T., Callie, B., Torres, A., Denman, A., Acosta, M., Fonseca, T., Lucas, J., Bornstein, E., Bonamer, J.. Evaluating Mediators of a Mindfulness-Based Stress Reduction (MBSR (BC)) Program on Cognitive Functioning and Symptom Response Among Breast Cancer Survivors (BCS). Psycho-oncology. 2022;31(SUPPL 1):92 https://doi.org/10.1002/pon.5872.

Lengacher, C., Park, J., Reich, R., Alinat, C., Ramesar, S., Le, A., Paterson, C., Park, H., Kiluk, J., Han, H., Ismail-Khan, R., Kip, K.. Genetic profiles and their role in a mindfulness-based stress reduction program for breast cancer survivors (MBSR(BC)). Psycho-oncology. 2017;26:82 https://doi.org/10.1002/pon.4354.

Lengacher, C., Reich, R., Paterson, C., Ramesar, S., Johnson-Mallard, V., Jacobsen, P., Alinat, C., Park, J.. A symptom cluster trial, the effects of mindfulness-based stress reduction (MBSR(BC)) on symptom clusters among breast cancer survivors (BCS). Psycho-oncology. 2015;24:96 https://doi.org/10.1002/pon.3873.

Luctkar-Flude, M., Groll, D., Beckett, L., Tyerman, J., Giroux, J.. Preliminary results of a mixed methods study of effects of neurofeedback on cognitive impairment and fatigue in post-treatment breast cancer survivors. Journal of complementary and integrative medicine. 2018;15(4):eA15.

Mao, J. J., Liou, K., Root, J., Li, Q. S., Bao, T., Garland, S. N., Ahles, T.. Acupuncture versus cognitive behavioral therapy for cognitive impairment in cancer survivors with insomnia: implications for personalized medicine. Journal of clinical oncology. 2019;37: https://doi.org/10.1200/JCO.2019.37.15suppl.11522.

Monninkhof, E., Koevoets, E. W., De Leede, M. L., De Ruiter, M. D., Schagen, S. B., May, A. M.. Long-term effects of physical exercise on self-reported cognitive function, fatigue, and quality of life in chemotherapy-treated breast cancer patients. Annals of oncology. 2022;33:S626 https://doi.org/10.1016/j.annonc.2022.07.232.

Noyes, K., Sahler, O. J., Zapf, A., Depner, R., Huston, A., McNeal, D., Flores, T., Rashid, H. H., Fleming, F. J., Constine, L. S.. Problem-solving skills training in adult cancer survivors: bright IDEAS-AC. Journal of clinical oncology. 2021;39(15 SUPPL): https://doi.org/10.1200/JCO.2021.39.15suppl.e24109.

Noyes, K., Zapf, A., Depner, R., Flores, T., Huston, A., Rashid, H. H., McNeal, D., Sahler, O. J., Constine, L. S., Fleming, F. J.. Feasibility of fully remote administration of problem-solving skills training (PSST) to adult cancer survivors in community settings. Journal of clinical oncology. 2021;39(15 SUPPL): https://doi.org/10.1200/JCO.2021.39.15suppl.1536.

Smith, T., Ratcliff, K.. Comparing Outcomes of Two Computer-Assisted Cognitive Rehabilitation Programs. Archives of physical medicine and rehabilitation. 2019;100(10):e56 https://doi.org/10.1016/j.apmr.2019.08.155.

Vardy, J. L., Pond, G. R., Bell, M. L., Renton, C., Dixon, A., Dhillon, H. M.. A randomized controlled trial evaluating two cognitive rehabilitation approaches for cancer survivors with perceived cognitive impairment. Asia-Pacific journal of clinical oncology. 2022;18:98 https://doi.org/10.1111/ajco.13868.

Zhang, Z. J.. Electroacupuncture for chemotherapy-associated insomnia and related psychiatric symptoms in breast cancer patients: randomized controlled trials. Sleep medicine. 2024;115:175 https://doi.org/10.1016/j.sleep.2023.11.496.

Trial registration

. Cognitive Training for Cancer Related Cognitive Impairment in Breast Cancer Survivors. Cognitive Training for Cancer Related Cognitive Impairment in Breast Cancer Survivors: a Multi-Center Randomized Double-Blinded Controlled Trial. 2023.

. [Public title] Evaluation of a brain training programme in cancer survivors self reporting cognitive changes; [Scientific title] Evaluation of a web-based cognitive rehabilitation programme in cancer survivors with self reported cognitive impairment. ANZCTR [www.anzctr.org.au]. 2009.

. ALDH-2 Enzyme in CRF With Advanced GI Cancer. The Efficacy and Safety of Alcoholic Dehydrogenase (ALDH)-2 Enzyme Supplement in Chemotherapy-Related Fatigue With Advanced Gastrointestinal Cancer Patients: a 2-Period, Crossover, Single-Center Study. 2021.

. Nicotinic Treatment of Post-Chemotherapy Subjective Cognitive Impairment: a Pilot Study. 2014.

. Cognitively-Based Compassion Training for Breast Cancer Survivors. Cognitively-Based Compassion Training (CBCT) for the Improvement of Health Related Quality of Life, Fear of Illness Recurrence, Compassion and Self-compassion in Breast Cancer Survivors Sample. 2017.

. Study of Donepezil in Female Breast Cancer Survivors With Cognitive Dysfunction. A Feasibility Study of Donepezil in Female Breast Cancer Survivors With Self-Reported Cognitive Dysfunction Following Chemotherapy. 2011.

. Cancer and Disorders of Cognitive Functions and Quality of Life: "Cognitive Rehabilitation in Patients Suffering From Cancer and Treated With Chemotherapy".. 2013.

. Repurposing Riluzole for Cancer-Related Cognitive Impairment: a Pilot Trial. Repurposing Riluzole for Augmenting Brain-Derived Neuropathic Factor (BDNF) Levels and Cognitive Function in Breast Cancer Patients Experiencing Cancer-Related Cognitive Impairment: an Interventional Pilot Clinical Trial. 2024.

Pre-print with published full text

Zimmerman, C. S., Temereanca, S., Daniels, D., Penner, C., Cannonier, T., Jones, S. R., Kerr, C.. The Vitality Project: A Randomized Control Trial Comparing Effects of Qigong and Exercise/Nutrition Training on Fatigue, Emotional Health, and Stress in Fatigued Female Cancer Survivors. medRxiv. 2022: https://dx.doi.org/10.1101/2022.08.18.22278965.

Non-English/French

윤희, 상, 황 은, 아. 재가암 환자 통합건강증진 프로그램 개발 및 효과. Journal of Korean Academy of Community Health Nursing / Jiyeog Sahoe Ganho Hakoeji. 2021;32(1):51-63 10.12799/jkachn.2021.32.1.51.

Non-RCT primary study design

Alberts, N. M., Hadjistavropoulos, H. D., Dear, B. F., Titov, N.. Internet-delivered cognitive-behaviour therapy for recent cancer survivors: a feasibility trial. Psycho-Oncology. 2017;26(1):137-139 https://dx.doi.org/10.1002/pon.4032.

Engan, Harald, Skanke, Frode, Dahl, Vegar, Eimhjellen Ryen, Eli Astrid, Lindgren, Kersti, Aasvik, Julie, Sandmæl, Jon Arne. The Feasibility of Web-Based Working Memory Training in Patients With Cancer Attending Inpatient Rehabilitation. Integrative Cancer Therapies. 2023;22:1-13 10.1177/15347354231164401.

Ercoli, Linda M., Castellon, Steven A., Hunter, Aimee M., Kwan, Lorna, Kahn-Mills, Barbara A., Cernin, Paul A., Leuchter, Andrew F., Ganz, Patricia A.. Assessment of the feasibility of a rehabilitation intervention program for breast cancer survivors with cognitive complaints. Brain imaging and behavior. 2013;7(4):543-53 https://dx.doi.org/10.1007/s11682-013-9237-0.

Pang, L., Bi, Z., Jing, Y., Yin, X., Zhang, X., Yao, S., Zhao, J., Cheng, H.. Changes in cytokine levels in breast cancer patients with CTRCI before or after CALM intervention. American Journal of Cancer Research. 2021;11(11):5415-5427.

Park, K. H., Lee, H., Park, E. Y., Sung, J. H., Song, M. K., An, M., Bang, E., Baek, S. Y., Do, Y., Lee, S., Lim, Y.. Effects of an urban forest healing program on cancer-related fatigue in cancer survivors. Supportive Care in Cancer. 2024;32(1):4 https://dx.doi.org/10.1007/s00520-023-08214-3.

Poppelreuter, M, Weis, J, Mumm, A, Orth, HB, Bartsch, HH. Rehabilitation of therapy-related cognitive deficits in patients after hematopoietic stem cell transplantation.. Bone marrow transplantation. 2008;41(1): 10.1038/sj.bmt.1705884.

Poppelreuter, M., Weis, J., Bartsch, H. H.. Effects of specific neuropsychological training programs for breast cancer patients after adjuvant chemotherapy. Journal of psychosocial oncology. 2009;27(2):274-96 https://dx.doi.org/10.1080/07347330902776044.

Sleight, Alix G., Myers, Jamie S., Cook-Wiens, Galen, Baynes, Rachel, Jo, Mi-Yeoung, Asher, Arash. Loneliness as a risk factor for cancer-related cognitive impairment: a secondary data analysis from the Haze study. Disability and rehabilitation. 2023;45(14):2325-2328 https://dx.doi.org/10.1080/09638288.2022.2089738.

Commentary/letter/editorial/opinion piece

Carlson, L. E.. Mindfulness Meditation for Younger Breast Cancer Survivors: A Randomized Controlled Trial. Breast Diseases. 2015;26(4):294-296 https://dx.doi.org/10.1016/j.breastdis.2015.10.002.

Hughes, M. K.. Comparing standard versus prosocial internet support groups for patients with breast cancer: A randomized controlled trial of the helper therapy principle: Lepore SJ, Buzaglo JS, Lieberman MA, et al (Temple Univ, Philadelphia, PA; Cancer Support Community, Philadelphia, PA; Univ of California, San Francisco) J Clin Oncol 32:4081-4086, 2014. Breast Diseases. 2015;26(2):121-122 https://dx.doi.org/10.1016/j.breastdis.2015.04.012.

Lim, C. E. D., Cheng, N. C. L.. Effect of mindfulness based stress reduction in stage 0-III breast cancer survivors. Focus on Alternative and Complementary Therapies. 2014;19(4):219-220 https://dx.doi.org/10.1111/fct.12135.

Van Dyk, K., Ganz, P. A.. Cancer-Related Cognitive Impairment in Patients with a History of Breast Cancer. JAMA - Journal of the American Medical Association. 2021;326(17):1736-1737 https://dx.doi.org/10.1001/jama.2021.13309.

Other non-primary study design

Grossman, Paul, Zwahlen, Diana, Halter, Jorg P., Passweg, Jakob R., Steiner, Claudia, Kiss, Alexander. A mindfulness-based program for improving quality of life among hematopoietic stem cell transplantation survivors: feasibility and preliminary findings. Supportive care in cancer: official journal of the Multinational Association of Supportive Care in Cancer. 2015;23(4):1105-12 https://dx.doi.org/10.1007/s00520-014-2452-4.

Kwon, M., Zhu, J., Wilding, G. E., Dickerson, S. S., Dean, G. E.. Sleep-wake state discrepancy among cancer survivors with insomnia symptoms. Supportive Care in Cancer. 2024;32(1):2 https://dx.doi.org/10.1007/s00520-023-08177-5.

Ineligible population

Abrahams, H. J. G., Gielissen, M. F. M., Braamse, A. M. J., Bleijenberg, G., Buffart, L. M., Knoop, H.. Graded activity is an important component in cognitive behavioral therapy to reduce severe fatigue: results of a pragmatic crossover trial in cancer survivors. Acta Oncologica. 2019;58(12):1692-1698 https://dx.doi.org/10.1080/0284186X.2019.1659513.

Admiraal, Jolien M., van der Velden, Annette W. G., Geerling, Jenske I., Burgerhof, Johannes G. M., Bouma, Grietje, Walenkamp, Annemiek M. E., de Vries, Elisabeth G. E., Schroder, Carolien P., Reyners, Anna K. L.. Web-Based Tailored Psychoeducation for Breast Cancer Patients at the Onset of the Survivorship Phase: A Multicenter Randomized Controlled Trial. Journal of pain and symptom management. 2017;54(4):466-475 https://dx.doi.org/10.1016/j.jpainsymman.2017.07.009.

Akechi, T., Furukawa, T. A., Noma, H., Iwata, H., Toyama, T., Higaki, K., Matsuoka, H., Zenda, S., Iwatani, T., Akahane, K., Inoue, A., Sagara, Y., Uchida, M., Imai, F., Momino, K., Imaizumi, G., Yamaguchi, T., Mashiko, T., Miyaji, T., Horikoshi, M., Sakurai, N., Onishi, T., Kanemitsu, Y., Murata, T., Wanifuchi-Endo, Y., Kuroda, H., Nishikawa, R., Miyashita, M., Abe, M., Uchitomi, Y.. Optimizing smartphone psychotherapy for depressive symptoms in patients with cancer: Multiphase optimization strategy using a decentralized multicenter randomized clinical trial (J-SUPPORT 2001 Study). Psychiatry and Clinical Neurosciences. 2024;78(6):353-361 https://dx.doi.org/10.1111/pcn.13657.

Amidi, A., Buskbjerg, C. R., Damholdt, M. F., Dahlgaard, J., Thorndike, F. P., Ritterband, L., Zachariae, R.. Changes in sleep following internet-delivered cognitive-behavioral therapy for insomnia in women treated for breast cancer: A 3-year follow-up assessment. Sleep Medicine. 2022;96:35-41 https://dx.doi.org/10.1016/j.sleep.2022.04.020.

Arana-Chicas, E., Lin, P. J., Gada, U., Sun, H., Chakrabarti, A., Mattick, L. J., Rieth, K., Chay, C. H., Ruzich, J., Esparaz, B. T., Cupertino, A. P., Altman, B. J., Vertino, P. M., Mohile, S. G., Mustian, K. M.. The effect of YOCAS© yoga on cancer-related fatigue and quality of life in older (60+) vs. younger (<= 59) cancer survivors: Secondary analysis of a nationwide, multicenter, phase 3 randomized controlled trial. Journal of Geriatric Oncology. 2024;15(8):102076 https://dx.doi.org/10.1016/j.jgo.2024.102076.

Arch, J. J., Mitchell, J. L., Genung, S. R., Judd, C. M., Andorsky, D. J., Bricker, J. B., Stanton, A. L.. Randomized trial of acceptance and commitment therapy for anxious cancer survivors in community clinics: Outcomes and moderators. Journal of Consulting and Clinical Psychology. 2021;89(4):327-340 https://dx.doi.org/10.1037/ccp0000630.

Bandani-Susan, Bahar, Montazeri, Ali, Haghighizadeh, Mohammad Hossein, Araban, Marzieh. The effect of mobile health educational intervention on body image and fatigue in breast cancer survivors: a randomized controlled trial. Irish journal of medical science. 2022;191(4):1599-1605 https://dx.doi.org/10.1007/s11845-021-02738-5.

Barton, D. L., Atherton, P. J., Satele, D. V., Qin, R., Dakhil, S., Pipe, T., Hobday, T., Fee-Schroeder, K., Loprinzi, C. L.. A randomized phase II trial evaluating two non-pharmacologic interventions in cancer survivors for the treatment of sleep-wake disturbances: NCCTG N07C4 (Alliance). Supportive Care in Cancer. 2020;28(12):6085-6094 https://dx.doi.org/10.1007/s00520-020-05461-6.

Barton, D. L., Liu, H., Dakhil, S. R., Linquist, B., Sloan, J. A., Nichols, C. R., McGinn, T. W., Stella, P. J., Seeger, G. R., Sood, A., Loprinzi, C. L.. Wisconsin ginseng (Panax quinquefolius) to improve cancer-related fatigue: A randomized, double-blind trial, N07C2. Journal of the National Cancer Institute. 2013;105(16):1230-1238 https://dx.doi.org/10.1093/jnci/djt181.

Bender, Catherine M., Sereika, Susan M., Gentry, Amanda L., Cuglewski, Cheryl, Duquette, Jennie, Grove, George, Cummings, Meredith, Cho, Myeong-Ga, Brufsky, Adam M., McAuliffe, Priscilla, Budway, Raye J., Diego, Emilia J., Evans, Steven, Rosenzweig, Margaret Q., Marsland, Anna L., Conley, Yvette P., Erickson, Kirk. Effects of aerobic exercise on neurocognitive function in postmenopausal women receiving endocrine therapy for breast cancer: The Exercise Program in Cancer and Cognition randomized controlled trial. Psycho-oncology. 2024;33(2): https://dx.doi.org/10.1002/pon.6298.

Bower, J. E., Garet, D., Sternlieb, B., Ganz, P. A., Irwin, M. R., Olmstead, R., Greendale, G.. Yoga for persistent fatigue in breast cancer survivors: A randomized controlled trial. Cancer. 2012;118(15):3766-3775 https://dx.doi.org/10.1002/cncr.26702.

Bower, Julienne E., Partridge, Ann H., Wolff, Antonio C., Cole, Steve W., Irwin, Michael R., Thorner, Elissa D., Joffe, Hadine, Petersen, Laura, Crespi, Catherine M., Ganz, Patricia A.. Improving biobehavioral health in younger breast cancer survivors: Pathways to Wellness trial secondary outcomes. Journal of the National Cancer Institute. 2023;115(1):83-92 https://dx.doi.org/10.1093/jnci/djac180.

Bower, Julienne E., Partridge, Ann H., Wolff, Antonio C., Thorner, Elissa D., Irwin, Michael R., Joffe, Hadine, Petersen, Laura, Crespi, Catherine M., Ganz, Patricia A.. Targeting Depressive Symptoms in Younger Breast Cancer Survivors: The Pathways to Wellness Randomized Controlled Trial of Mindfulness Meditation and Survivorship Education. Journal of clinical oncology : official journal of the American Society of Clinical Oncology. 2021;39(31):3473-3484 https://dx.doi.org/10.1200/JCO.21.00279.

Bulfone, T., Quattrin, R., Zanotti, R., Regattin, L., Brusaferro, S.. Effectiveness of music therapy for anxiety reduction in women with breast cancer in chemotherapy treatment. Holistic nursing practice. 2009;23(4):238-242.

Campo, R. A., Agarwal, N., LaStayo, P. C., O'Connor, K., Pappas, L., Boucher, K. M., Gardner, J., Smith, S., Light, K. C., Kinney, A. Y.. Levels of fatigue and distress in senior prostate cancer survivors enrolled in a 12-week randomized controlled trial of Qigong. Journal of Cancer Survivorship. 2014;8(1):60-69 https://dx.doi.org/10.1007/s11764-013-0315-5.

Carlson, L. E., Tamagawa, R., Speca, M., Faris, P., Doll, R., Stephen, J., Drysdale, E.. Randomized controlled trial of

mindfulness-based cancer recovery versus supportive expressive group therapy for distressed survivors of breast cancer (MINDSET). Journal of Clinical Oncology. 2013;31(25):3119-3126 https://dx.doi.org/10.1200/JCO.2012.47.5210.

Carlson, Linda E., Beattie, Tara L., Giese-Davis, Janine, Faris, Peter, Tamagawa, Rie, Fick, Laura J., Degelman, Erin S., Speca, Michael. Mindfulness-based cancer recovery and supportive-expressive therapy maintain telomere length relative to controls in distressed breast cancer survivors. Cancer. 2015;121(3):476-84 https://dx.doi.org/10.1002/cncr.29063.

Carlson, Linda E., Tamagawa, Rie, Stephen, Joanne, Drysdale, Elaine, Zhong, Lihong, Speca, Michael. Randomized-controlled trial of mindfulness-based cancer recovery versus supportive expressive group therapy among distressed breast cancer survivors (MINDSET): long-term follow-up results. Psycho-oncology. 2016;25(7):750-9 https://dx.doi.org/10.1002/pon.4150.

Cheng, Tse-Chou, Lee, Yi-Hua, Mar, Chun-Lin, Huang, Wen-Tsung, Chang, Yuan-Ping. The Health Promoting Mindfulness or Qigong Educational Programs for Beneficial Lifestyle Changes of Cancer Survivors. Journal of cancer education : the official journal of the American Association for Cancer Education. 2020;35(4):743-750 https://dx.doi.org/10.1007/s13187-019-01522-5.

Classen, C. C., Chivers, M. L., Urowitz, S., Barbera, L., Wiljer, D., O'Rinn, S., Ferguson, S. E.. Psychosexual distress in women with gynecologic cancer: A feasibility study of an online support group. Psycho-Oncology. 2013;22(4):930-935 https://dx.doi.org/10.1002/pon.3058.

Cohen, Caroline W., Fontaine, Kevin R., Arend, Rebecca C., Soleymani, Taraneh, Gower, Barbara A.. Favorable Effects of a Ketogenic Diet on Physical Function, Perceived Energy, and Food Cravings in Women with Ovarian or Endometrial Cancer: A Randomized, Controlled Trial. Nutrients. 2018;10(9): https://dx.doi.org/10.3390/nu10091187.

Costa, D., Mogos, I., Toma, T.. Efficacy and safety of mianserin in the treatment of depression of women with cancer. Acta Psychiatrica Scandinavica. 1985;72(SUPPL. 320):85-92.

Culos-Reed, S. Nicole, Carlson, Linda E., Daroux, Lisa M., Hately-Aldous, Susi. A pilot study of yoga for breast cancer survivors: physical and psychological benefits. Psycho-oncology. 2006;15(10):891-7.

Custers, J. A. E., Kwakkenbos, L., Levis, B., Doking, S., van der Hoeven, Y. C. W., Leermakers, L., de Wilt, J. H. W., Thewes, B., Braamse, A. M. J., Dekker, J., Prins, J. B.. Randomized controlled trial of an individual blended cognitive behavioral therapy to reduce psychological distress among distressed colorectal cancer survivors: The COloRectal canceR distrEss reduCTion trial. Psycho-Oncology. 2024;33(1):e6270 https://dx.doi.org/10.1002/pon.6270.

D'Alessandro E, G., Da Silva, A. V., Cecatto, R. B., Brito, C. M. M. D., Azevedo, R. S., Lin, C. A.. Acupuncture for Climacteric-Like Symptoms in Breast Cancer Improves Sleep, Mental and Emotional Health: A Randomized Trial. Medical Acupuncture. 2022;34(1):58-65 https://dx.doi.org/10.1089/acu.2021.0073.

Davis, Cindy, Rust, Connie, Choi, Sam. A pilot randomized study of skills training for African American cancer survivors. Social work in public health. 2014;29(6):549-60 https://dx.doi.org/10.1080/19371918.2014.892865.

Deng, Gary, Bao, Ting, Ryan, Elizabeth L., Benusis, Lara, Hogan, Pasha, Li, Qing S., Dries, Annika, Konner, Jason, Ahles,

Tim A., Mao, Jun J.. Effects of Vigorous Versus Restorative Yoga Practice on Objective Cognition Functions in Sedentary Breast and Ovarian Cancer Survivors: A Randomized Controlled Pilot Trial. Integrative cancer therapies. 2022;21:15347354221089221 https://dx.doi.org/10.1177/15347354221089221.

Dolev, Talya, Ben-David, Merav, Shahadi, Ilanit, Freed, Yaakov, Zubedat, Salman, Aga-Mizrachi, Shlomit, Brand, Zev, Galper, Shira, Jacobson, Galia, Avital, Avi. Attention Dysregulation in Breast Cancer Patients Following a Complementary Alternative Treatment Routine: A Double-Blind Randomized Trial. Integrative cancer therapies. 2021;20:15347354211019470 https://dx.doi.org/10.1177/15347354211019470.

Escalante, Carmen P., Meyers, Christina, Reuben, James M., Wang, Xuemei, Qiao, Wei, Manzullo, Ellen, Alvarez, Ricardo H., Morrow, Phuong Khanh, Gonzalez-Angulo, Ana M., Wang, Xin Shelley, Mendoza, Tito, Liu, Wenli, Holmes, Holly, Hwang, Jessica, Pisters, Katherine, Overman, Michael, Cleeland, Charles. A randomized, double-blind, 2-period, placebo-controlled crossover trial of a sustained-release methylphenidate in the treatment of fatigue in cancer patients. Cancer journal (Sudbury, Mass.). 2014;20(1):8-14 https://dx.doi.org/10.1097/PPO.0000000000000018.

Fernandez-Lao, C., Cantarero-Villanueva, I., Diaz-Rodriguez, L., Cuesta-Vargas, A. I., Fernandez-Delas-Penas, C., Arroyo-Morales, M.. Attitudes towards massage modify effects of manual therapy in breast cancer survivors: A randomised clinical trial with crossover design. European Journal of Cancer Care. 2012;21(2):233-241 https://dx.doi.org/10.1111/j.1365-2354.2011.01306.x.

Fernandez-Rodriguez, Concepcion, Gonzalez-Fernandez, Sonia, Coto-Lesmes, Rocio, Pedrosa, Ignacio. Behavioral Activation and Acceptance and Commitment Therapy in the Treatment of Anxiety and Depression in Cancer Survivors: A Randomized Clinical Trial. Behavior modification. 2021;45(5):822-859 https://dx.doi.org/10.1177/0145445520916441.

Fishbein, J. N., Judd, C. M., Genung, S., Stanton, A. L., Arch, J. J.. Intervention and mediation effects of target processes in a randomized controlled trial of Acceptance and Commitment Therapy for anxious cancer survivors in community oncology clinics. Behaviour Research and Therapy. 2022;153:104103 https://dx.doi.org/10.1016/j.brat.2022.104103.

Foster, Claire, Grimmett, Chloe, May, Christine M., Ewings, Sean, Myall, Michelle, Hulme, Claire, Smith, Peter W., Powers, Cassandra, Calman, Lynn, Armes, Jo, Breckons, Matthew, Corner, Jessica, Fenlon, Deborah, Batehup, Lynn, Lennan, Elaine, R May, Carl, Morris, Carolyn, Neylon, Amanda, Ream, Emma, Turner, Lesley, Yardley, Lucy, Richardson, Alison. A web-based intervention (RESTORE) to support self-management of cancer-related fatigue following primary cancer treatment: a multi-centre proof of concept randomised controlled trial. Supportive care in cancer : official journal of the Multinational Association of Supportive Care in Cancer. 2016;24(6):2445-53 https://dx.doi.org/10.1007/s00520-015-3044-7.

Freeman, Lyn W., White, Rebecca, Ratcliff, Chelsea G., Sutton, Sue, Stewart, Mary, Palmer, J. Lynn, Link, Judith, Cohen, Lorenzo. A randomized trial comparing live and telemedicine deliveries of an imagery-based behavioral intervention for breast cancer survivors: reducing symptoms and barriers to care. Psycho-oncology. 2015;24(8):910-8 https://dx.doi.org/10.1002/pon.3656.

Garland, S. N., Roscoe, J. A., Heckler, C. E., Barilla, H., Gehrman, P., Findley, J. C., Peoples, A. R., Morrow, G. R., Kamen, C., Perlis, M. L.. Effects of armodafinil and cognitive behavior therapy for insomnia on sleep continuity and daytime sleepiness in cancer survivors. Sleep Medicine. 2016;20:18-24 https://dx.doi.org/10.1016/j.sleep.2015.12.010.

Garland, Sheila N., Tulk, Joshua, Savard, Josee, Rash, Joshua A., Browne, Sondria, Urquhart, Robin, Seal, Melanie, Thoms, John, Laing, Kara. Randomized Controlled Trial of Virtually Delivered Cognitive Behavioral Therapy for Insomnia to Address Perceived Cancer-Related Cognitive Impairment in Cancer Survivors. Journal of clinical oncology : official journal of the American Society of Clinical Oncology. 2024;42(17):2094-2104 https://dx.doi.org/10.1200/JCO.23.02330.

Goedendorp, Martine M., Knoop, Hans, Gielissen, Marieke F. M., Verhagen, Constans A. H. H. V. M., Bleijenberg, Gijs. The effects of cognitive behavioral therapy for postcancer fatigue on perceived cognitive disabilities and neuropsychological test performance. Journal of pain and symptom management. 2014;47(1):35-44 https://dx.doi.org/10.1016/j.jpainsymman.2013.02.014.

Gonzalez-Fernandez, Sonia, Fernandez-Rodriguez, Concepcion, Paz-Caballero, Maria D., Perez-Alvarez, Marino. Treating anxiety and depression of cancer survivors: Behavioral activation versus acceptance and commitment therapy. Psicothema. 2018;30(1):14-20 https://dx.doi.org/10.7334/psicothema2017.396.

Goodarzian, Mehrnaz, Mohammadi, Kourosh, Amirfakhraei, Azita. Effectiveness of Acceptance and Commitment Therapy (ACT) on Health-Related Locus of Control and Cognitive Regulation of Emotion in Women with Breast Cancer. Women's Health Bulletin. 2023;10(4):2-10 10.30476/WHB.2023.99974.1245.

Hall, D. L., Arditte Hall, K. A., Gorman, M. J., Comander, A., Goldstein, M. R., Cunningham, T. J., Wieman, S., Mizrach, H. R., Juhel, B. C., Li, R., Markowitz, A., Grandner, M., Park, E. R.. The Survivorship Sleep Program (SSP): A synchronous, virtual cognitive behavioral therapy for insomnia pilot program among cancer survivors. Cancer. 2022;128(7):1532-1544 https://dx.doi.org/10.1002/cncr.34066.

Han, J., Jang, M. K., Lee, H., Kim, S. Y., Kim, S. H., Hee Ko, Y., Song, Y., Kang, M. J., Jeon, J. Y., Cho, Y. U., Yi, G., Kim, S.. Long Term Effects of a Social Capital-Based Exercise Adherence Intervention for Breast Cancer Survivors With Moderate Fatigue: A Randomized Controlled Trial. Integrative Cancer Therapies. 2023;22: https://dx.doi.org/10.1177/15347354231209440.

Hardy, J., Greer, R., Huggett, G., Kearney, A., Gurgenci, T., Good, P.. Phase IIb Randomized, Placebo-Controlled, Dose-Escalating, Double-Blind Study of Cannabidiol Oil for the Relief of Symptoms in Advanced Cancer (MedCan1-CBD). Journal of Clinical Oncology. 2023;41(7):1444-1452 https://dx.doi.org/10.1200/JCO.22.01632.

Hoxtermann, M. D., Buner, K., Haller, H., Kohl, W., Dobos, G., Reinisch, M., Kummel, S., Cramer, H., Voiss, P.. Efficacy and safety of auricular acupuncture for the treatment of insomnia in breast cancer survivors: A randomized controlled trial. Cancers. 2021;13(16):4082 https://dx.doi.org/10.3390/cancers13164082.

Huddar, V., Vernekar, S., Tatpati, N., Thali, S., D'Silva, P. V.. Comparative effect of progressive relaxation technique and Benson's technique on anxiety and fatigue in cancer survivors - an experimental study. Current Problems in Cancer. 2023;47(1):100933 https://dx.doi.org/10.1016/j.currproblcancer.2022.100933.

Hyland, Kelly A., Nelson, Ashley M., Eisel, Sarah L., Hoogland, Aasha I., Ibarz-Pinilla, Javier, Sweet, Kendra, Jacobsen, Paul B., Knoop, Hans, Jim, Heather S. L.. Fatigue Perpetuating Factors as Mediators of Change in a Cognitive Behavioral

Intervention for Targeted Therapy-Related Fatigue in Chronic Myeloid Leukemia: A Pilot Study. Annals of behavioral medicine : a publication of the Society of Behavioral Medicine. 2022;56(2):137-145 https://dx.doi.org/10.1093/abm/kaab035.

Irwin, M. R., Olmstead, R., Breen, E. C., Witarama, T., Carrillo, C., Sadeghi, N., Arevalo, J. M. G., Ma, J., Nicassio, P., Ganz, P. A., Bower, J. E., Cole, S.. Tai Chi, cellular inflammation, and transcriptome dynamics in breast cancer survivors with insomnia: A randomized controlled trial. Journal of the National Cancer Institute - Monographs. 2014;2014(50):295-301 https://dx.doi.org/10.1093/jncimonographs/lgu028.

Irwin, M. R., Olmstead, R., Carrillo, C., Sadeghi, N., Nicassio, P., Ganz, P. A., Bower, J. E.. Tai Chi Chih compared with cognitive behavioral therapy for the Treatment of Insomnia in Survivors of Breast Cancer: A randomized, partially blinded, noninferiority trial. Journal of Clinical Oncology. 2017;35(23):2656-2665 https://dx.doi.org/10.1200/JCO.2016.71.0285.

Jakobsen, G., Sjue, K., Paulsen, O., Kaasa, S., Hjermstad, M. J., Klepstad, P.. Zopiclone versus placebo for short-term treatment of insomnia in patients with advanced cancer-a double-blind, randomized placebo-controlled clinical multicenter phase IV trial. Supportive Care in Cancer. 2023;31(1):60 https://dx.doi.org/10.1007/s00520-022-07537-x.

Janelsins, Michelle C., Peppone, Luke J., Heckler, Charles E., Kesler, Shelli R., Sprod, Lisa K., Atkins, James, Melnik, Marianne, Kamen, Charles, Giguere, Jeffrey, Messino, Michael J., Mohile, Supriya G., Mustian, Karen M.. YOCAS© R Yoga Reduces Self-reported Memory Difficulty in Cancer Survivors in a Nationwide Randomized Clinical Trial: Investigating Relationships Between Memory and Sleep. Integrative cancer therapies. 2016;15(3):263-71 https://dx.doi.org/10.1177/1534735415617021.

Jang, M. K., Han, J., Kim, S. H., Ko, Y. H., Kim, S. Y., Kim, S.. Comparison of fatigue and fatigability correlates in Korean breast cancer survivors and differences in associations with anxiety, depression, sleep disturbance, and endocrine symptoms: a randomized controlled trial. BMC Cancer. 2021;21(1):855 https://dx.doi.org/10.1186/s12885-021-08575-0.

Jiang, L., Xu, J., Wu, Y., Liu, Y., Wang, X., Hu, Y.. Effects of the "AI-TA" Mobile App With Intelligent Design on Psychological and Related Symptoms of Young Survivors of Breast Cancer: Randomized Controlled Trial. JMIR mHealth and uHealth. 2024;12:e50783 https://dx.doi.org/10.2196/50783.

Jim, Heather S. L., Hyland, Kelly A., Nelson, Ashley M., Pinilla-Ibarz, Javier, Sweet, Kendra, Gielissen, Marieke, Bulls, Hailey, Hoogland, Aasha I., Jacobsen, Paul B., Knoop, Hans. Internet-assisted cognitive behavioral intervention for targeted therapy-related fatigue in chronic myeloid leukemia: Results from a pilot randomized trial. Cancer. 2020;126(1):174-180 https://dx.doi.org/10.1002/cncr.32521.

Johns, Shelley A., Brown, Linda F., Beck-Coon, Kathleen, Monahan, Patrick O., Tong, Yan, Kroenke, Kurt. Randomized controlled pilot study of mindfulness-based stress reduction for persistently fatigued cancer survivors. Psycho-oncology. 2015;24(8):885-93 https://dx.doi.org/10.1002/pon.3648.

Johns, Shelley A., Brown, Linda F., Beck-Coon, Kathleen, Talib, Tasneem L., Monahan, Patrick O., Giesler, R. Brian, Tong, Yan, Wilhelm, Laura, Carpenter, Janet S., Von Ah, Diane, Wagner, Christina D., de Groot, Mary, Schmidt, Karen, Monceski, Diane, Danh, Marie, Alyea, Jennifer M., Miller, Kathy D., Kroenke, Kurt. Randomized controlled pilot trial

of mindfulness-based stress reduction compared to psychoeducational support for persistently fatigued breast and colorectal cancer survivors. Supportive care in cancer : official journal of the Multinational Association of Supportive Care in Cancer. 2016;24(10):4085-96 https://dx.doi.org/10.1007/s00520-016-3220-4.

Johns, Shelley A., Von Ah, Diane, Brown, Linda F., Beck-Coon, Kathleen, Talib, Tasneem L., Alyea, Jennifer M., Monahan, Patrick O., Tong, Yan, Wilhelm, Laura, Giesler, R. Brian. Randomized controlled pilot trial of mindfulness-based stress reduction for breast and colorectal cancer survivors: effects on cancer-related cognitive impairment. Journal of cancer survivorship : research and practice. 2016;10(3):437-48 https://dx.doi.org/10.1007/s11764-015-0494-3.

Johnson, J. A., Garland, S. N., Carlson, L. E., Savard, J., Simpson, J. S. A., Ancoli-Israel, S., Campbell, T. S.. Bright light therapy improves cancer-related fatigue in cancer survivors: a randomized controlled trial. Journal of cancer survivorship : research and practice. 2018;12(2):206-215 https://dx.doi.org/10.1007/s11764-017-0659-3.

Johnston, Michael F., Hays, Ron D., Subramanian, Saskia K., Elashoff, Robert M., Axe, Eleanor K., Li, Jie-Jia, Kim, Irene, Vargas, Roberto B., Lee, Jihey, Yang, LuGe, Hui, Ka-Kit. Patient education integrated with acupuncture for relief of cancer-related fatigue randomized controlled feasibility study. BMC complementary and alternative medicine. 2011;11:49 https://dx.doi.org/10.1186/1472-6882-11-49.

JR Berenson, O Yellin, HK Shamasunder, CS Chen, V Charu, TB Woliver, S Sanani, M Schlutz, Y Nassir, RA Swift, C Andreu-Vieyra, R Vescio. A phase 3 trial of armodafinil for the treatment of cancer-related fatigue for patients with multiple myeloma.. Supportive care in cancer : official journal of the Multinational Association of Supportive Care in Cancer. 2015;23(6): 10.1007/s00520-014-2486-7.

Kemerer, Bibiana M., Zdaniuk, Bozena, Higano, Celestia S., Bossio, Jennifer A., Camara Bicalho Santos, Raysa, Flannigan, Ryan, Brotto, Lori A.. A randomized comparison of group mindfulness and group cognitive behavioral therapy vs control for couples after prostate cancer with sexual dysfunction. The journal of sexual medicine. 2023;20(3):346-366 https://dx.doi.org/10.1093/jsxmed/qdac038.

Kesler, Shelli, Hadi Hosseini, S. M., Heckler, Charles, Janelsins, Michelle, Palesh, Oxana, Mustian, Karen, Morrow, Gary. Cognitive training for improving executive function in chemotherapy-treated breast cancer survivors. Clinical breast cancer. 2013;13(4):299-306 https://dx.doi.org/10.1016/j.clbc.2013.02.004.

Kim, S., Ko, Y. H., Song, Y., Kang, M. J., Lee, H., Kim, S. H., Jeon, J. Y., Cho, Y. U., Yi, G., Han, J.. Pre-post analysis of a social capital-based exercise adherence intervention for breast cancer survivors with moderate fatigue: a randomized controlled trial. Supportive Care in Cancer. 2020;28(11):5281-5289 https://dx.doi.org/10.1007/s00520-020-05363-7.

Kleinknecht, Karl R., Bierend, Mira, Keim, Lisa-Maria, Bartels, Frederik, Lampit, Amit, Finke, Carsten. Computerized cognitive training improves cognitive function in primary breast cancer survivors. NPJ breast cancer. 2024;10(1):85 https://dx.doi.org/10.1038/s41523-024-00694-8.

Kohli, Sadhna, Fisher, Susan G., Tra, Yolande, Adams, M. Jacob, Mapstone, Mark E., Wesnes, Keith A., Roscoe, Joseph A., Morrow, Gary R.. The effect of modafinil on cognitive function in breast cancer survivors. Cancer. 2009;115(12):2605-16 https://dx.doi.org/10.1002/cncr.24287.

Korstjens, Irene, Mesters, Ilse, May, Anne M., van Weert, Ellen, van den Hout, Johanna H. C., Ros, Wynand, Hoekstra-Weebers, Josette E. H. M., van der Schans, Cees P., van den Borne, Bart. Effects of cancer rehabilitation on problem-solving, anxiety and depression: a RCT comparing physical and cognitive-behavioural training versus physical training. Psychology & health. 2011;26 Suppl 1:63-82 https://dx.doi.org/10.1080/08870441003611569.

Krebber, A. M. H., Jansen, F., Witte, B. I., Cuijpers, P., de Bree, R., Becker-Commissaris, A., Smit, E. F., van Straten, A., Eeckhout, A. M., Beekman, A. T. F., Leemans, C. R., Verdonck-de Leeuw, I. M.. Stepped care targeting psychological distress in head and neck cancer and lung cancer patients: a randomized, controlled trial. Annals of oncology : official journal of the European Society for Medical Oncology. 2016;27(9):1754-60 https://dx.doi.org/10.1093/annonc/mdw230.

Lengacher, Cecile A., Reich, Richard R., Paterson, Carly L., Ramesar, Sophia, Park, Jong Y., Alinat, Carissa, Johnson-Mallard, Versie, Moscoso, Manolete, Budhrani-Shani, Pinky, Miladinovic, Branko, Jacobsen, Paul B., Cox, Charles E., Goodman, Matthew, Kip, Kevin E.. Examination of Broad Symptom Improvement Resulting From Mindfulness-Based Stress Reduction in Breast Cancer Survivors: A Randomized Controlled Trial. Journal of clinical oncology : official journal of the American Society of Clinical Oncology. 2016;34(24):2827-34 https://dx.doi.org/10.1200/JCO.2015.65.7874.

Lepore, S. J., Buzaglo, J. S., Lieberman, M. A., Golant, M., Greener, J. R., Davey, A.. Comparing standard versus prosocial internet support groups for patients with breast cancer: A randomized controlled trial of the helper therapy principle. Journal of Clinical Oncology. 2014;32(36):4081-4086 https://dx.doi.org/10.1200/JCO.2014.57.0093.

Li, J., Xie, J., Guo, X., Fu, R., Wang, Y., Guan, X.. Effects of Mind-Regulation Acupuncture Therapy on Serum Ghrelin, Gastric Inhibitory Polypeptide, Leptin, and Insulin Levels in Breast Cancer Survivors with Cancer-Related Fatigue: A Randomized Controlled Trial. International Journal of General Medicine. 2023;16:1017-1027 https://dx.doi.org/10.2147/IJGM.S405977.

Li, X., Liou, K. T., Chimonas, S., Bryl, K., Wong, G., Spiguel, E., Li, S. Q., Garland, S. N., Bao, T., Mao, J. J.. Addressing cancer-related fatigue through sleep: A secondary analysis of a randomized trial comparing acupuncture and cognitive behavioral therapy for insomnia. Integrative Medicine Research. 2023;12(1):100922 https://dx.doi.org/10.1016/j.imr.2023.100922.

Lin, P. J., Altman, B. J., Gilmore, N. J., Loh, K. P., Dunne, R. F., Bautista, J., Fung, C., Janelsins, M. C., Peppone, L. J., Melnik, M. K., Gococo, K. O., Messino, M. J., Mustian, K. M.. Effect of Yoga and Mediational Influence of Fatigue on Walking, Physical Activity, and Quality of Life Among Cancer Survivors. JNCCN Journal of the National Comprehensive Cancer Network. 2023;21(2):153-162 https://dx.doi.org/10.6004/jnccn.2022.7080.

Lin, Zheng-Gen, Li, Ren-Dong, Ai, Fu-Lu, Li, Song, Zhang, Xin-An. Effects of cognitive behavior therapy combined with Baduanjin in patients with colorectal cancer. World journal of gastrointestinal oncology. 2022;14(1):319-333 https://dx.doi.org/10.4251/wjgo.v14.i1.319.

Liou, Kevin T., Garland, Sheila N., Li, Q. Susan, Sadeghi, Keimya, Green, Jamie, Autuori, Isidora, Orlow, Irene, Mao, Jun J.. Effects of acupuncture versus cognitive behavioral therapy on brain-derived neurotrophic factor in cancer survivors with insomnia: an exploratory analysis. Acupuncture in medicine : journal of the British Medical Acupuncture Society. 2021;39(6):637-645 https://dx.doi.org/10.1177/0964528421999395.

Liou, Kevin T., Root, James C., Garland, Sheila N., Green, Jamie, Li, Yuelin, Li, Q. Susan, Kantoff, Philip W., Ahles, Tim A., Mao, Jun J.. Effects of acupuncture versus cognitive behavioral therapy on cognitive function in cancer survivors with insomnia: A secondary analysis of a randomized clinical trial. Cancer. 2020;126(13):3042-3052 https://dx.doi.org/10.1002/cncr.32847.

Lipschitz, David L., Kuhn, Renee, Kinney, Anita Y., Donaldson, Gary W., Nakamura, Yoshio. Reduction in salivary alpha-amylase levels following a mind-body intervention in cancer survivors--an exploratory study. Psychoneuroendocrinology. 2013;38(9):1521-31 https://dx.doi.org/10.1016/j.psyneuen.2012.12.021.

Liu, Weimin, Liu, Juan, Ma, Lan, Chen, Jing. Effect of mindfulness yoga on anxiety and depression in early breast cancer patients received adjuvant chemotherapy: a randomized clinical trial. Journal of cancer research and clinical oncology. 2022;148(9):2549-2560 https://dx.doi.org/10.1007/s00432-022-04167-y.

Liu, X., Yuan, K., Ye, X., Liu, R.. Proposing a novel care program: reminiscence therapy involved care for anxiety, depression, and quality of life in postoperative cervical cancer patients. Irish journal of medical science. 2022;:2019 https://doi.org/10.1007/s11845-021-02728-7.

Lleras de Frutos, M., Medina, J. C., Vives, J., Casellas-Grau, A., Marzo, J. L., Borras, J. M., Ochoa-Arnedo, C.. Video conference vs face-to-face group psychotherapy for distressed cancer survivors: A randomized controlled trial. Psycho-Oncology. 2020;29(12):1995-2003 https://dx.doi.org/10.1002/pon.5457.

Lower, Elyse E., Fleishman, Stewart, Cooper, Alyse, Zeldis, Jerome, Faleck, Herbert, Yu, Zhinuan, Manning, Donald. Efficacy of dexmethylphenidate for the treatment of fatigue after cancer chemotherapy: a randomized clinical trial. Journal of pain and symptom management. 2009;38(5):650-62 https://dx.doi.org/10.1016/j.jpainsymman.2009.03.011.

Lundorff, L. E., Jonsson, B. H., Sjogren, P.. Modafinil for attentional and psychomotor dysfunction in advanced cancer: A double-blind, randomised, cross-over trial. Palliative Medicine. 2009;23(8):731-738 https://dx.doi.org/10.1177/0269216309106872.

Maheu, Christine, Lebel, Sophie, Bernstein, Lori J., Courbasson, Christine, Singh, Mina, Ferguson, Sarah E., Harris, Cheryl, Jolicoeur, Lynne, Baku, Lorena, Muraca, Linda, Ramanakumar, Agnihotram V., Lamonde, Frederic, Lefebvre, Monique, Tomei, Christina, Mutsaers, Brittany, Secord, Scott, Power, Joanne, Drummond, Nancy, Hébert, Maude, Wani, Rajvi J.. Fear of Cancer Recurrence Therapy (FORT): A Randomized Controlled Trial. Health Psychology. 2023;42(3):182-194 10.1037/hea0001253.

Mehnert, Anja, Veers, Silke, Howaldt, Dirk, Braumann, Klaus-Michael, Koch, Uwe, Schulz, Karl-Heinz. Effects of a physical exercise rehabilitation group program on anxiety, depression, body image, and health-related quality of life among breast cancer patients. Onkologie. 2011;34(5):248-53 https://dx.doi.org/10.1159/000327813.

Melnikov, V., Tiburcio-Jimenez, D., Mendoza-Hernandez, M. A., Delgado-Enciso, J., De-Leon-Zaragoza, L., Guzman-Esquivel, J., Rodriguez-Sanchez, I. P., Martinez-Fierro, M. L., Lara-Esqueda, A., Delgado-Enciso, O. G., Jacinto-Cortes, I., Zaizar-Fregoso, S. A., Paz-Michel, B. A., Murillo-Zamora, E., Delgado-Enciso, I., Galvan-Salazar, H. R.. Improve cognitive impairment using mefenamic acid non-steroidal anti-inflammatory therapy: Additional beneficial effect found in a controlled clinical trial for prostate cancer therapy. American Journal of Translational Research. 2021;13(5):4535-4543.

Meneses, Karen, Benz, Rachel, Bail, Jennifer R., Vo, Jacqueline B., Triebel, Kristen, Fazeli, Pariya, Frank, Jennifer, Vance, David E.. Speed of processing training in middle-aged and older breast cancer survivors (SOAR): results of a randomized controlled pilot. Breast cancer research and treatment. 2018;168(1):259-267 https://dx.doi.org/10.1007/s10549-017-4564-2.

Milne, Helen M., Wallman, Karen E., Gordon, Sandy, Courneya, Kerry S.. Effects of a combined aerobic and resistance exercise program in breast cancer survivors: a randomized controlled trial. Breast cancer research and treatment. 2008;108(2):279-88.

Molassiotis, A., Bardy, J., Finnegan-John, J., Mackereth, P., Ryder, W. D., Filshie, F., Ream, E., Eaton, D., Richardson, A.. A randomized, controlled trial of acupuncture self-needling as maintenance therapy for cancer-related fatigue after therapist-delivered acupuncture. Annals of Oncology. 2013;24(6):1645-1652 https://dx.doi.org/10.1093/annonc/mdt034.

Molassiotis, A., Sylt, P., Diggins, H.. The management of cancer-related fatigue after chemotherapy with acupuncture and acupressure: A randomised controlled trial. Complementary Therapies in Medicine. 2007;15(4):228-237 https://dx.doi.org/10.1016/j.ctim.2006.09.009.

Monti, Daniel A., Tobia, Anna, Stoner, Marie, Wintering, Nancy, Matthews, Michael, He, Xiao-Song, Doucet, Gaelle, Chervoneva, Inna, Tracy, Joseph I., Newberg, Andrew B.. Neuro emotional technique effects on brain physiology in cancer patients with traumatic stress symptoms: preliminary findings. Journal of cancer survivorship : research and practice. 2017;11(4):438-446 https://dx.doi.org/10.1007/s11764-017-0601-8.

Mulick, Amy, Walker, Jane, Puntis, Stephen, Symeonides, Stefan, Gourley, Charlie, Burke, Katy, Wanat, Marta, Frost, Chris, Sharpe, Michael. Is improvement in comorbid major depression associated with longer survival in people with cancer? A long-term follow-up of participants in the SMaRT Oncology-2 and 3 trials. Journal of Psychosomatic Research. 2019;116:106-112 https://dx.doi.org/10.1016/j.jpsychores.2018.11.008.

Muller, Fabiola, Wijayanto, Feri, Abrahams, Harriet, Gielissen, Marieke, Prinsen, Hetty, Braamse, Annemarie, van Laarhoven, Hanneke W. M., Groot, Perry, Heskes, Tom, Knoop, Hans. Potential mechanisms of the fatigue-reducing effect of cognitive-behavioral therapy in cancer survivors: Three randomized controlled trials. Psycho-oncology. 2021;30(9):1476-1484 https://dx.doi.org/10.1002/pon.5710.

Mustian, K. M., Sprod, L. K., Janelsins, M., Peppone, L. J., Palesh, O. G., Chandwani, K., Reddy, P. S., Melnik, M. K., Heckler, C., Morrow, G. R.. Multicenter, randomized controlled trial of yoga for sleep quality among cancer survivors. Journal of clinical oncology : official journal of the American Society of Clinical Oncology. 2013;31(26):3233-3241 http://dx.doi.org/10.1200/JCO.2012.43.7707.

Nakamura, Yoshio, Lipschitz, David L., Kuhn, Renee, Kinney, Anita Y., Donaldson, Gary W.. Investigating efficacy of two brief mind-body intervention programs for managing sleep disturbance in cancer survivors: a pilot randomized controlled trial. Journal of cancer survivorship : research and practice. 2013;7(2):165-82 https://dx.doi.org/10.1007/s11764-012-0252-8.

Ngu, Siew-Fei, Wei, Na, Li, Justin, Chu, Mandy M. Y., Tse, Ka Yu, Ngan, Hextan Y. S., Chan, Karen K. L.. Nurse-led

follow-up in survivorship care of gynaecological malignancies—A randomised controlled trial. European Journal of Cancer Care. 2020;29(6):1-8 10.1111/ecc.13325.

Nissen, Eva Rames, O'Connor, Maja, Kaldo, Viktor, Hojris, Inger, Borre, Michael, Zachariae, Robert, Mehlsen, Mimi. Internet-delivered mindfulness-based cognitive therapy for anxiety and depression in cancer survivors: A randomized controlled trial. Psycho-oncology. 2020;29(1):68-75 https://dx.doi.org/10.1002/pon.5237.

Nourizadeh, R., Khanipour, S., Zamiri, R. E., Namin, M. S., Khalili, A., Hakimi, S.. A Comparison of the Effects of Self-Acupressure and Aerobic Exercises on Sleep Disorders of Breast Cancer Survivors: A Controlled Randomized Clinical Trial. Medical Acupuncture. 2022;34(2):131-136 https://dx.doi.org/10.1089/acu.2020.1515.

Noyes, Katia, Zapf, Alaina L., Depner, Rachel M., Flores, Tessa, Huston, Alissa, Rashid, Hani H., McNeal, Demetria, Constine, Louis S., Fleming, Fergal J., Wilding, Gregory E., Sahler, Olle Jane Z.. Problem-solving skills training in adult cancer survivors: Bright IDEAS-AC pilot study. Cancer treatment and research communications. 2022;31:100552 https://dx.doi.org/10.1016/j.ctarc.2022.100552.

Oh, Hyunsung, Ell, Kathleen. Social support, a mediator in collaborative depression care for cancer patients. Research on Social Work Practice. 2015;25(2):229-239 https://dx.doi.org/10.1177/1049731514525240.

Oswald, L. B., Morales-Cruz, J., Eisel, S. L., Del Rio, J., Hoogland, A. I., Ortiz-Rosado, V., Soto-Lopez, G., Rodriguez-Rivera, E., Savard, J., Castro, E., Jim, H. S. L., Gonzalez, B. D.. Pilot randomized controlled trial of eHealth cognitive-behavioral therapy for insomnia among Spanish-speaking breast cancer survivors. Journal of behavioral medicine. 2022;45(3):503-508 https://dx.doi.org/10.1007/s10865-022-00313-6.

Owen, J. E., O'Carroll Bantum, E., Pagano, I. S., Stanton, A.. Randomized Trial of a Social Networking Intervention for Cancer-Related Distress. Annals of behavioral medicine : a publication of the Society of Behavioral Medicine. 2017;51(5):661-672 https://dx.doi.org/10.1007/s12160-017-9890-4.

Pagola, Itiziar, Morales, Javier S., Alejo, Lidia B., Barcelo, Olga, Montil, Marta, Olivan, Jesus, Alvarez-Bustos, Alejandro, Cantos, Blanca, Maximiano, Constanza, Hidalgo, Francisco, Valenzuela, Pedro L., Fiuza-Luces, Carmen, Lucia, Alejandro, Ruiz-Casado, Ana. Concurrent Exercise Interventions in Breast Cancer Survivors with Cancer-related Fatigue. International journal of sports medicine. 2020;41(11):790-797 https://dx.doi.org/10.1055/a-1147-1513.

Peng, Li, Yang, Ying, Chen, Muyu, Xu, Chen, Chen, Yanli, Liu, Rongqian, Cao, Xiaozhen, Li, Min. Effects of an online mindfulness-based intervention on Fear of Cancer Recurrence and quality of life among Chinese breast cancer survivors. Complementary therapies in clinical practice. 2022;49:101686 https://dx.doi.org/10.1016/j.ctcp.2022.101686.

Reich, Richard R., Lengacher, Cecile A., Alinat, Carissa B., Kip, Kevin E., Paterson, Carly, Ramesar, Sophia, Han, Heather S., Ismail-Khan, Roohi, Johnson-Mallard, Versie, Moscoso, Manolete, Budhrani-Shani, Pinky, Shivers, Steve, Cox, Charles E., Goodman, Matthew, Park, Jong. Mindfulness-Based Stress Reduction in Post-treatment Breast Cancer Patients: Immediate and Sustained Effects Across Multiple Symptom Clusters. Journal of pain and symptom management. 2017;53(1):85-95 https://dx.doi.org/10.1016/j.jpainsymman.2016.08.005.

Rini, Christine, Austin, Jane, Wu, Lisa M., Winkel, Gary, Valdimarsdottir, Heiddis, Stanton, Annette L., Isola, Luis,

Rowley, Scott, Redd, William H.. Harnessing benefits of helping others: a randomized controlled trial testing expressive helping to address survivorship problems after hematopoietic stem cell transplant. Health psychology : official journal of the Division of Health Psychology, American Psychological Association. 2014;33(12):1541-51 https://dx.doi.org/10.1037/hea0000024.

Ritterband, L. M., Bailey, E. T., Thorndike, F. P., Lord, H. R., Farrell-Carnahan, L., Baum, L. D.. Initial evaluation of an Internet intervention to improve the sleep of cancer survivors with insomnia. Psycho-Oncology. 2012;21(7):695-705 https://dx.doi.org/10.1002/pon.1969.

Sahin, F., Ozkaraman, A., Irmak Kaya, Z.. The effect of a combined treatment of foot soak and lavender oil inhalation therapy on the severity of insomnia of patients with cancer: Randomized interventional study. Explore. 2023;19(3):426-433 https://dx.doi.org/10.1016/j.explore.2022.09.003.

Savard, J., Ivers, H., Savard, M. H., Morin, C. M.. Long-term effects of two formats of cognitive behavioral therapy for insomnia comorbid with breast cancer. Sleep. 2016;39(4):813-823 https://dx.doi.org/10.5665/sleep.5634.

Savard, J., Simard, S., Ivers, H., Morin, C. M.. Randomized study on the efficacy of cognitive-behavioral therapy for insomnia secondary to breast cancer, part I: Sleep and psychological effects. Journal of Clinical Oncology. 2005;23(25):6083-6096 https://dx.doi.org/10.1200/JCO.2005.09.548.

Savard, J., Simard, S., Ivers, H., Morin, C. M.. Randomized study on the efficacy of cognitive-behavioral therapy for insomnia secondary to breast cancer, part II: Immunologic effects. Journal of Clinical Oncology. 2005;23(25):6097-6106 https://dx.doi.org/10.1200/JCO.2005.12.513.

Schad, Friedemann, Rieser, Thomas, Becker, Sarah, Groß, Jessica, Matthes, Harald, Oei, Shiao Li, Thronicke, Anja. Efficacy of Tango Argentino for Cancer-Associated Fatigue and Quality of Life in Breast Cancer Survivors: A Randomized Controlled Trial. Cancers. 2023;15(11):2920 10.3390/cancers15112920.

Schellekens, Melanie P. J., Bruggeman-Everts, Fieke Z., Wolvers, Marije D. J., Vollenbroek-Hutten, Miriam M. R., van der Lee, Marije L.. What web-based intervention for chronic cancer-related fatigue works best for whom? Explorative moderation analyses of a randomized controlled trial. Supportive care in cancer : official journal of the Multinational Association of Supportive Care in Cancer. 2022;30(10):7885-7892 https://dx.doi.org/10.1007/s00520-022-07223-y.

Schellekens, Melanie P. J., Tamagawa, Rie, Labelle, Laura E., Speca, Michael, Stephen, Joanne, Drysdale, Elaine, Sample, Sarah, Pickering, Barbara, Dirkse, Dale, Savage, Linette Lawlor, Carlson, Linda E.. Mindfulness-Based Cancer Recovery (MBCR) versus Supportive Expressive Group Therapy (SET) for distressed breast cancer survivors: evaluating mindfulness and social support as mediators. Journal of behavioral medicine. 2017;40(3):414-422 https://dx.doi.org/10.1007/s10865-016-9799-6.

Shari, Nurul Izzah, Zainal, Nor Zuraida, Ng, Chong Guan. Effects of brief acceptance and commitment therapy (ACT) on subjective cognitive impairment in breast cancer patients undergoing chemotherapy. Journal of psychosocial oncology. 2021;39(6):695-714 https://dx.doi.org/10.1080/07347332.2020.1856283.

Shi, Ru-Chun, Meng, Ai-Feng, Zhou, Weng-Lin, Yu, Xiao-Yan, Huang, Xin-En, Ji, Ai-Jun, Chen, Lei. Effects of Home

Nursing Intervention on the Quality of Life of Patients with Nasopharyngeal Carcinoma after Radiotherapy and Chemotherapy. Asian Pacific journal of cancer prevention : APJCP. 2015;16(16):7117-21.

Sikorskii, A., Badger, T., Segrin, C., Crane, T. E., Chalasani, P., Arslan, W., Hadeed, M., Morrill, K. E., Given, C.. A Sequential Multiple Assignment Randomized Trial of Symptom Management After Chemotherapy. Journal of Pain and Symptom Management. 2023;65(6):541-552.e2 https://dx.doi.org/10.1016/j.jpainsymman.2023.02.005.

Smith, Theresa M., Wang, Wanyi. Comparison of a standard computer-assisted cognitive training program to a music enhanced program: A mixed methods study. Cancer reports (Hoboken, N.J.). 2021;4(2):e1325 https://dx.doi.org/10.1002/cnr2.1325.

Spahrkas, S. S., Looijmans, A., Sanderman, R., Hagedoorn, M.. Beating cancer-related fatigue with the Untire mobile app: Results from a waiting-list randomized controlled trial. Psycho-Oncology. 2020;29(11):1823-1834 https://dx.doi.org/10.1002/pon.5492.

Spahrkas, Simon S., Looijmans, Anne, Sanderman, Robbert, Hagedoorn, Mariet. How does the Untire app alleviate cancer-related fatigue? A longitudinal mediation analysis. Psycho-oncology. 2022;31(6):970-977 https://dx.doi.org/10.1002/pon.5886.

Spathis, A., Dhillan, R., Booden, D., Forbes, K., Vrotsou, K., Fife, K.. Modafinil for the treatment of fatigue in lung cancer: A pilot study. Palliative Medicine. 2009;23(4):325-331 https://dx.doi.org/10.1177/0269216309102614.

Spathis, A., Fife, K., Blackhall, F., Dutton, S., Bahadori, R., Wharton, R., O'Brien, M., Stone, P., Benepal, T., Bates, N., Wee, B.. Modafinil for the treatment of fatigue in lung cancer: Results of a placebo-controlled, double-blind, randomized trial. Journal of Clinical Oncology. 2014;32(18):1882-1888 https://dx.doi.org/10.1200/JCO.2013.54.4346.

Syrjala, K. L., Walsh, C. A., Yi, J. C., Leisenring, W. M., Rajotte, E. J., Voutsinas, J., Ganz, P. A., Jacobs, L. A., Palmer, S. C., Partridge, A., Baker, K. S.. Cancer survivorship care for young adults: a risk-stratified, multicenter randomized controlled trial to improve symptoms. Journal of cancer survivorship : research and practice. 2022;16(5):1149-1164 https://dx.doi.org/10.1007/s11764-021-01105-8.

Taylor, K. L., Lamdan, R. M., Siegel, J. E., Shelby, R., Moran-Klimi, K., Hrywna, M.. Psychological adjustment among African American breast cancer patients: One-year follow-up results of a randomized psychoeducational group intervention. Health Psychology. 2003;22(3):316-323 https://dx.doi.org/10.1037/0278-6133.22.3.316.

Tong, Taishan, Pei, Chunqin, Chen, Jun, Lv, Qing, Zhang, Fuquan, Cheng, Zaohuo. Efficacy of Acupuncture Therapy for Chemotherapy-Related Cognitive Impairment in Breast Cancer Patients. Medical science monitor : international medical journal of experimental and clinical research. 2018;24:2919-2927 https://dx.doi.org/10.12659/MSM.909712.

Van Der Lee, M. L., Garssen, B.. Mindfulness-based cognitive therapy reduces chronic cancer-related fatigue: A treatment study. Psycho-Oncology. 2012;21(3):264-272 https://dx.doi.org/10.1002/pon.1890.

van der Lee, Marije L., Carssen, Bert. Mindfulness-based cognitive therapy reduces cancer-related chronic fatigue: A treatment study. Minder moe na kanker door aandachtsgerichte cognitieve therapie.. 2013;46(2):89-105.

Viruega, Hélène, Galy, Corinne, Loriette, Célia, Jacquot, Stéphane, Houpeau, Jean Louis, Gaviria, Manuel. Breast Cancer: How Hippotherapy Bridges the Gap between Healing and Recovery—A Randomized Controlled Clinical Trial. Cancers. 2023;15(4):1317 10.3390/cancers15041317.

Vo, Jacqueline B., Fazeli, Pariya L., Benz, Rachel, Bail, Jennifer R., Triebel, Kristen, Vance, David E., Meneses, Karen. Effects of a speed of processing training intervention on self-reported health outcomes in breast cancer survivors. Nursing: Research & Reviews. 2019;9:13-19 10.2147/NRR.S199664.

Vranceanu, Ana-Maria, Bannon, Sarah, Mace, Ryan, Lester, Ethan, Meyers, Emma, Gates, Melissa, Popok, Paula, Lin, Ann, Salgueiro, Danielle, Tehan, Tara, Macklin, Eric, Rosand, Jonathan. Feasibility and Efficacy of a Resiliency Intervention for the Prevention of Chronic Emotional Distress Among Survivor-Caregiver Dyads Admitted to the Neuroscience Intensive Care Unit: A Randomized Clinical Trial. JAMA network open. 2020;3(10):e2020807 https://dx.doi.org/10.1001/jamanetworkopen.2020.20807.

Wieman, S. T., Arditte Hall, K. A., Park, E. R., Gorman, M. J., Comander, A., Goldstein, M. R., Cunningham, T. J., Mizrach, H. R., Juhel, B., Li, R., Markowitz, A., Grandner, M., Liverant, G. I., Hall, D. L.. Treatment-related changes in insomnia, anticipatory pleasure, and depression symptoms: A proof-of-concept study with cancer survivors. Sleep Medicine. 2023;103:29-32 https://dx.doi.org/10.1016/j.sleep.2023.01.011.

Wilkinson, S. M., Love, S. B., Westcombe, A. M., Gambles, M. A., Burgess, C. C., Cargill, A., Young, T., Maher, E. J., Ramirez, A. J.. Effectiveness of aromatherapy massage in the management of anxiety and depression in patients with cancer: A multicenter randomized controlled trial. Journal of Clinical Oncology. 2007;25(5):532-539 https://dx.doi.org/10.1200/JCO.2006.08.9987.

Wright, A. A., Poort, H., Tavormina, A., Schmiege, S. J., Matulonis, U. A., Campos, S. M., Liu, J. F., Slivjak, E. T., Gilmour, A. L., Salinger, J. M., Haggerty, A. F., Arch, J. J.. Pilot randomized trial of an acceptance-based telehealth intervention for women with ovarian cancer and PARP inhibitor-related fatigue. Gynecologic Oncology. 2023;177:165-172 https://dx.doi.org/10.1016/j.ygyno.2023.08.020.

Xia, Shuli. Effect of cognitive behavioral stress management on anxiety, depression, and quality of life in colorectal cancer patients post tumor resection: a randomized, controlled study. Irish journal of medical science. 2023;192(4):1637-1644 https://dx.doi.org/10.1007/s11845-023-03405-7.

Yennurajalingam, S., Carmack, C., Balachandran, D., Eng, C., Lim, B., Delgado, M., Guzman Gutierrez, D., Raznahan, M., Park, M., Hess, K. R., Williams, J. L., Lu, Z., Ochoa, J., Bruera, E.. Sleep disturbance in patients with cancer: A feasibility study of multimodal therapy. BMJ Supportive and Palliative Care. 2021;11(2):170-179 https://dx.doi.org/10.1136/bmjspcare-2019-001877.

Yennurajalingam, S., Frisbee-Hume, S., Palmer, J. L., Delgado-Guay, M. O., Bull, J., Phan, A. T., Tannir, N. M., Litton, J. K., Reddy, A., Hui, D., Dalal, S., Massie, L., Reddy, S. K., Bruera, E.. Reduction of cancer-related fatigue with dexamethasone: A double-blind, randomized, placebo-controlled trial in patients with advanced cancer. Journal of Clinical Oncology. 2013;31(25):3076-3082 https://dx.doi.org/10.1200/JCO.2012.44.4661.

Yennurajalingam, S., Valero, V., Lu, Z., Liu, D. D., Busaidy, N. L., Reuben, J. M., Fleming, C. D., Williams, J. L., Hess, K. R., Basen-Engquist, K., Bruera, E.. Combination Therapy of Physical Activity and Dexamethasone for Cancer-Related Fatigue: A Phase II Randomized Double-Blind Controlled Trial. JNCCN Journal of the National Comprehensive Cancer Network. 2022;20(3):235-243 https://dx.doi.org/10.6004/JNCCN.2021.7066.

Zachariae, R., Amidi, A., Damholdt, M. F., Clausen, C. D. R., Dahlgaard, J., Lord, H., Thorndike, F. P., Ritterband, L. M.. Internet-Delivered cognitive-Behavioral therapy for insomnia in Breast cancer survivors: A randomized controlled trial. Journal of the National Cancer Institute. 2018;110(8):880-887 https://dx.doi.org/10.1093/jnci/djx293.

Zernicke, Kristin A., Campbell, Tavis S., Speca, Michael, McCabe-Ruff, Kelley, Flowers, Steven, Carlson, Linda E.. A randomized wait-list controlled trial of feasibility and efficacy of an online mindfulness-based cancer recovery program: the eTherapy for cancer applying mindfulness trial. Psychosomatic medicine. 2014;76(4):257-67 https://dx.doi.org/10.1097/PSY.0000000000000053.

Zetzl, T., Renner, A., Pittig, A., Jentschke, E., Roch, C., van Oorschot, B.. Yoga effectively reduces fatigue and symptoms of depression in patients with different types of cancer. Supportive Care in Cancer. 2021;29(6):2973-2982 https://dx.doi.org/10.1007/s00520-020-05794-2.

Zhang, J., Qin, Z., So, T. H., Chang, T. Y., Yang, S., Chen, H., Yeung, W. F., Chung, K. F., Chan, P. Y., Huang, Y., Xu, S., Chiang, C. Y., Lao, L., Zhang, Z. J.. Acupuncture for chemotherapy-associated insomnia in breast cancer patients: an assessor-participant blinded, randomized, sham-controlled trial. Breast Cancer Research. 2023;25(1):49 https://dx.doi.org/10.1186/s13058-023-01645-0.

Zhang, J., Qin, Z., So, T. H., Chen, H., Lam, W. L., Yam, L. L., Yan Chan, P., Lao, L., Zhang, Z. J.. Electroacupuncture Plus Auricular Acupressure for Chemotherapy-Associated Insomnia in Breast Cancer Patients: A Pilot Randomized Controlled Trial. Integrative Cancer Therapies. 2021;20: https://dx.doi.org/10.1177/15347354211019103.

Zhang, Jia-Yuan, Li, Sha-Sha, Meng, Li-Na, Zhou, Yu-Qiu. Effectiveness of a nurse-led Mindfulness-based Tai Chi Chuan (MTCC) program on Posttraumatic Growth and perceived stress and anxiety of breast cancer survivors. European journal of psychotraumatology. 2022;13(1):2023314 https://dx.doi.org/10.1080/20008198.2021.2023314.

Zhang, Jia-Yuan, Zhou, Yu-Qiu, Feng, Zi-Wei, Fan, Yi-Nan, Zeng, Guang-Chun, Wei, Li. Randomized controlled trial of mindfulness-based stress reduction (MBSR) on posttraumatic growth of Chinese breast cancer survivors. Psychology, health & medicine. 2017;22(1):94-109.

Zhao, C., Diao, W., Xu, X., Li, L., Jia, Z.. Effect of cognitive behavior therapy based on the health education pathway on psychology of papillary thyroid carcinoma patients: a randomized controlled trial. Nuclear Medicine Communications. 2024;45(4):304-311 https://dx.doi.org/10.1097/MNM.0000000000001818.

Zhao, H., Zhang, Q., Zhao, L., Huang, X., Wang, J., Kang, X.. Spore powder of Ganoderma lucidum improves cancer-related fatigue in breast cancer patients undergoing endocrine therapy: A pilot clinical trial. Evidence-based Complementary and Alternative Medicine. 2012;2012:809614 https://dx.doi.org/10.1155/2012/809614.

Zhao, Yue, Liu, Jun- E., Lewis, Frances Marcus, Nie, Zhi-Hong, Qiu, Hui, Han, Jing, Su, Ya-Li, Yang, Shen-Shen. Effects

of mindfulness-based cognitive therapy on breast cancer survivors with insomnia: A randomised controlled trial. European journal of cancer care. 2020;29(5):e13259 https://dx.doi.org/10.1111/ecc.13259.

Zhao, Yue, Liu, Jun- E., Lewis, Frances Marcus, Nie, Zhi-Hong, Qiu, Hui, Han, Jing, Su, Ya-Li, Yang, Shen-Shen. Effects of mindfulness-based cognitive therapy on breast cancer survivors with insomnia: A randomised controlled trial. European Journal of Cancer Care. 2020;29(5):1-12 10.1111/ecc.13259.

Zick, S. M., Sen, A., Hassett, A. L., Schrepf, A., Wyatt, G. K., Murphy, S. L., Arnedt, J. T., Harris, R. E.. Impact of Self-Acupressure on Co-Occurring Symptoms in Cancer Survivors. JNCI Cancer Spectrum. 2018;2(4):pky064 https://dx.doi.org/10.1093/JNCICS/PKY064.

Zick, S. M., Sen, A., Wyatt, G. K., Murphy, S. L., Arnedt, J. T., Harris, R. E.. Investigation of 2 Types of Self-administered Acupressure for Persistent Cancer-Related Fatigue in Breast Cancer Survivors: A Randomized Clinical Trial. JAMA oncology. 2016;2(11):1470-1476 https://dx.doi.org/10.1001/jamaoncol.2016.1867.

Zimmerman, C. S., Temereanca, S., Daniels, D., Penner, C., Cannonier, T., Jones, S. R., Kerr, C.. A Randomized Controlled Pilot Trial Comparing Effects of Qigong and Exercise/Nutrition Training on Fatigue and Other Outcomes in Female Cancer Survivors. Integrative Cancer Therapies. 2023;22: https://dx.doi.org/10.1177/15347354231162584.

No relevant quantitative data

Henneghan, Ashley M., Fico, Brandon G., Wright, Michelle L., Kesler, Shelli R., Harrison, Michelle L.. Effects of meditation compared to music listening on biomarkers in breast cancer survivors with cognitive complaints: secondary outcomes of a pilot randomized control trial. Explore (New York, N.Y.). 2022;18(6):657-662 https://dx.doi.org/10.1016/j.explore.2021.10.011.

Koevoets, E. W., Geerlings, M. I., Monninkhof, E. M., Mandl, R., Witlox, L., van der Wall, E., Stuiver, M. M., Sonke, G. S., Velthuis, M. J., Jobsen, J. J., van der Palen, J., Bos, M. E. M. M., Goker, E., Menke-Pluijmers, M. B. E., Sommeijer, D. W., May, A. M., de Ruiter, M. B., Schagen, S. B.. Effect of physical exercise on the hippocampus and global grey matter volume in breast cancer patients: A randomized controlled trial (PAM study). NeuroImage. Clinical. 2023;37:103292 https://dx.doi.org/10.1016/j.nicl.2022.103292.

Koevoets, Emmie W., Petr, Jan, Monninkhof, Evelyn M., Geerlings, Mirjam I., Witlox, Lenja, van der Wall, Elsken, Stuiver, Martijn M., Sonke, Gabe S., Velthuis, Miranda J., Jobsen, Jan J., van der Palen, Job, Mutsaerts, Henk J. M. M., de Ruiter, Michiel B., May, Anne M., Schagen, Sanne B.. Effect of Physical Exercise on MRI-Assessed Brain Perfusion in Chemotherapy-Treated Breast Cancer Patients: A Randomized Controlled Trial. Journal of magnetic resonance imaging : JMRI. 2024;59(5):1667-1680 https://dx.doi.org/10.1002/jmri.28967.

Melis, Michelle, Blommaert, Jeroen, Radwan, Ahmed, Smeets, Ann, Van der Gucht, Katleen, Deprez, Sabine, Sunaert, Stefan. Structural brain changes after a mindfulness-based intervention in breast cancer survivors with cognitive complaints. Mindfulness. 2023;:No-Specified https://dx.doi.org/10.1007/s12671-023-02140-5.

Melis, Michelle, Blommaert, Jeroen, Van der Gucht, Katleen, Smeets, Ann, McDonald, Brenna C., Sunaert, Stefan, Smith, Andra, Deprez, Sabine. The impact of mindfulness on working memory-related brain activation in breast cancer survivors with cognitive complaints. Journal of cancer survivorship : research and practice. 2023;:

https://dx.doi.org/10.1007/s11764-023-01484-0.

Melis, Michelle, Schroyen, Gwen, Blommaert, Jeroen, Leenaerts, Nicolas, Smeets, Ann, Van Der Gucht, Katleen, Sunaert, Stefan, Deprez, Sabine. The Impact of Mindfulness on Functional Brain Connectivity and Peripheral Inflammation in Breast Cancer Survivors with Cognitive Complaints. Cancers. 2023;15(14): https://dx.doi.org/10.3390/cancers15143632.

Vega, Jennifer N., Albert, Kimberly M., Mayer, Ingrid A., Taylor, Warren D., Newhouse, Paul A.. Subjective cognition and mood in persistent chemotherapy-related cognitive impairment. Journal of cancer survivorship : research and practice. 2022;16(3):614-623 https://dx.doi.org/10.1007/s11764-021-01055-1.

Yao, Senbang, Ding, Ke, Liu, Shaochun, Zhang, Qianqian, Li, Wen, Tang, Lingxue, Yu, Sheng, Pang, Lulian, Yin, Xiangxiang, Cheng, Huaidong. The Managing Cancer and Living Meaningfully (CALM) Intervention Alleviates Chemotherapy-Related Cognitive Impairment in Patients with Breast Cancer by Modulating Pan-Immune-Inflammation Values. Integrative cancer therapies. 2022;21:15347354221140498 https://dx.doi.org/10.1177/15347354221140498.

Excluded for other reasons

Bellens, Anne, Roelant, Ella, Sabbe, Bernard, Peeters, Marc, van Dam, Peter A.. A video-game based cognitive training for breast cancer survivors with cognitive impairment: A prospective randomized pilot trial. Breast (Edinburgh, Scotland). 2020;53:23-32 https://dx.doi.org/10.1016/j.breast.2020.06.003.

Bray, Victoria J., Dhillon, Haryana M., Bell, Melanie L., Kabourakis, Michael, Fiero, Mallorie H., Yip, Desmond, Boyle, Frances, Price, Melanie A., Vardy, Janette L.. Evaluation of a Web-Based Cognitive Rehabilitation Program in Cancer Survivors Reporting Cognitive Symptoms After Chemotherapy. Journal of clinical oncology : official journal of the American Society of Clinical Oncology. 2017;35(2):217-225.

Chapman, Bethany, Louis, Courtney C., Moser, Jason, Grunfeld, Elizabeth A., Derakshan, Nazanin. Benefits of adaptive cognitive training on cognitive abilities in women treated for primary breast cancer: Findings from a 1-year randomised control trial intervention. Psycho-oncology. 2023;32(12):1848-1857 https://dx.doi.org/10.1002/pon.6232.

Ding, Ke, Zhang, Xiuqing, Zhao, Jingjing, Zuo, He, Bi, Ziran, Cheng, Huaidong. Managing Cancer and Living Meaningfully (CALM) Intervention on Chemotherapy-Related Cognitive Impairment in Breast Cancer Survivors. Integrative cancer therapies. 2020;19:1534735420938450 https://dx.doi.org/10.1177/1534735420938450.

Hartman, Sheri J., Nelson, Sandahl H., Myers, Emily, Natarajan, Loki, Sears, Dorothy D., Palmer, Barton W., Weiner, Lauren S., Parker, Barbara A., Patterson, Ruth E.. Randomized controlled trial of increasing physical activity on objectively measured and self-reported cognitive functioning among breast cancer survivors: The memory & motion study. Cancer. 2018;124(1):192-202 https://dx.doi.org/10.1002/cncr.30987.

Klaver, Kete M., Duijts, Saskia F. A., Geusgens, Chantal A. V., Kieffer, Jacobien M., Agelink van Rentergem, Joost, Hendriks, Mathijs P., Nuver, Janine, Marsman, Hendrik A., Poppema, Boelo J., Oostergo, Tanja, Doeksen, Annemiek, Aarts, Maureen J. B., Ponds, Rudolf W. H. M., van der Beek, Allard J., Schagen, Sanne B.. Internet-based cognitive rehabilitation for working cancer survivors: results of a multicenter randomized controlled trial. JNCI cancer spectrum. 2024;8(1): https://dx.doi.org/10.1093/jncics/pkad110.

Maeir, Talia, Makranz, Chen, Peretz, Tamar, Odem, Ester, Tsabari, Shani, Nahum, Mor, Gilboa, Yafit. Cognitive Retraining and Functional Treatment (CRAFT) for adults with cancer related cognitive impairment: a preliminary efficacy study. Supportive care in cancer : official journal of the Multinational Association of Supportive Care in Cancer. 2023;31(3):152 https://dx.doi.org/10.1007/s00520-023-07611-y.

Mihuta, Mary E., Green, Heather J., Shum, David H. K.. Web-based cognitive rehabilitation for survivors of adult cancer: A randomised controlled trial. Psycho-oncology. 2018;27(4):1172-1179 https://dx.doi.org/10.1002/pon.4615.

Myers, Jamie S., Mitchell, Melissa, Krigel, Susan, Steinhoff, Andreanna, Boyce-White, Alyssa, Van Goethem, Karla, Valla, Mary, Dai, Junqiang, He, Jianghua, Liu, Wen, Sereika, Susan M., Bender, Catherine M.. Qigong intervention for breast cancer survivors with complaints of decreased cognitive function. Supportive care in cancer : official journal of the Multinational Association of Supportive Care in Cancer. 2019;27(4):1395-1403 https://dx.doi.org/10.1007/s00520-018-4430-8.

Myers, Jamie S., Wertheimer, Jeffrey C., Kim, Jaromme Geebum, He, Jianghua, Shirazipour, Celina H., Hooper, Dedrick, Penne Mays, Mary, Klemp, Jennifer R., Zegers, Carli, Asher, Arash. Emerging From the Haze TM: Pilot Feasibility Study Comparing Two Virtual Formats of a Cognitive Rehabilitation Intervention. Oncology nursing forum. 2024;51(3):223-242 https://dx.doi.org/10.1188/24.ONF.223-242.

Sarvghadi, P., Ghaffari, A., Rostami, H. R.. The effects of neurofeedback training on short-term memory and quality of life in women with breast cancer. International journal of therapy and rehabilitation. 2019;26(11):1.

Tack, Laura, Lefebvre, Tessa, Lycke, Michelle, Langenaeken, Chistine, Fontaine, Christel, Borms, Marleen, Hanssens, Marianne, Knops, Christel, Meryck, Kathleen, Boterberg, Tom, Pottel, Hans, Schofield, Patricia, Debruyne, Philip R.. A randomised wait-list controlled trial to evaluate Emotional Freedom Techniques for self-reported cancer-related cognitive impairment in cancer survivors (EMOTICON). EClinicalMedicine. 2021;39:101081 https://dx.doi.org/10.1016/j.eclinm.2021.101081.

Takemura, Naomi, Cheung, Denise Shuk Ting, Fong, Daniel Yee Tak, Lee, Anne Wing Mui, Lam, Tai-Chung, Ho, James Chung-Man, Kam, Tsz Yeung, Chik, Jeannie Yin Kwan, Lin, Chia-Chin. Comparative effect of Tai Chi and aerobic exercise on cognitive function in advanced lung cancer survivors with perceived cognitive impairment: a three-arm randomized controlled trial with mediation analysis. Journal of cancer survivorship : research and practice. 2024;: https://dx.doi.org/10.1007/s11764-024-01607-1.

Vega, Jennifer N., Albert, Kimberly M., Mayer, Ingrid A., Taylor, Warren D., Newhouse, Paul A.. Nicotinic treatment of post-chemotherapy subjective cognitive impairment: a pilot study. Journal of cancer survivorship : research and practice. 2019;13(5):673-686 https://dx.doi.org/10.1007/s11764-019-00786-6.

Von Ah, Diane, Carpenter, Janet S., Saykin, Andrew, Monahan, Patrick, Wu, Jingwei, Yu, Menggang, Rebok, George, Ball, Karlene, Schneider, Bryan, Weaver, Michael, Tallman, Eileen, Unverzagt, Fred. Advanced cognitive training for breast cancer survivors: a randomized controlled trial. Breast cancer research and treatment. 2012;135(3):799-809 https://dx.doi.org/10.1007/s10549-012-2210-6.

Wu, Lisa M., Amidi, Ali, Tanenbaum, Molly L., Winkel, Gary, Gordon, Wayne A., Hall, Simon J., Bovbjerg, Katrin,

Diefenbach, Michael A.. Computerized cognitive training in prostate cancer patients on androgen deprivation therapy: a pilot study. Supportive care in cancer : official journal of the Multinational Association of Supportive Care in Cancer. 2018;26(6):1917-1926 https://dx.doi.org/10.1007/s00520-017-4026-8.

Wyant, Sheryl. Feasibility and acceptability of a computerized working memory training in breast cancer survivors. Dissertation Abstracts International: Section B: The Sciences and Engineering. 2018;79(1-B(E)):No-Specified.

Yao, Senbang, Zhu, Qinqin, Zhang, Qianqian, Cai, Yinlian, Liu, Shaochun, Pang, Lulian, Jing, Yanyan, Yin, Xiangxiang, Cheng, Huaidong. Managing Cancer and Living Meaningfully (CALM) alleviates chemotherapy related cognitive impairment (CTRCI) in breast cancer survivors: A pilot study based on resting-state fMRI. Cancer medicine. 2023;: https://dx.doi.org/10.1002/cam4.6285.

File S6: Findings from transitivity exploration

Transitivity was explored both subjectively, using box plots and data tables, and semi-objectively, using recently published methodology[1]. For the primary analyses, subjective explorations of transitivity suggested that compared to other intervention contrasts, COG/EDU_GD_GRP vs waitlist (n = 1 study[15]) was evaluated in the oldest patients (mean age 59 years), with the most years of education (mean 16.9 years), who had severe cognitive impairment and fatigue at baseline and on average had completed chemotherapy for the longest duration (53 months). Also, exercise interventions and their associated controls were on average of longer duration than other interventions (i.e., 25 weeks vs 7.3 weeks). Box plots of effect modifiers by treatment comparison were prepared for investigation by the study team; we have provided these plots for the learning outcome from our analyses (see further below; this represented the most well populated network), while analogous information for other outcomes are available upon request from the authors.
Further visual assessments other than by treatment comparison suggested that feasibility studies (n = 3)[16-18] potentially differed from RCTs in sample size (smaller), baseline cognitive impairment (more severe), and time since chemotherapy (fewer months).
Semi-objective transitivity assessment[1] was conducted on the learning and cognitive flexibility domains, which both had the highest number of treatment comparisons across all outcomes (n = 17), although with slightly differing comparisons in both outcomes. These assessments suggested possible intransitivity within two comparisons: EXE_GD_GRP vs waitlist and MBI vs waitlist. The two studies evaluating EXE_GD_GRP vs waitlist differed across several effect modifiers of interest: one was a very small (n = 19) feasibility study of highly-educated breast cancer survivors (70% received a college or post-graduate degree), with severe cognitive impairment at baseline, who had finished chemotherapy on average less than 12 months before recruitment, while the second was a large (n = 181) RCT of moderately educated breast cancer survivors (43% 'high' education level), with moderate cognitive impairment at baseline, who had finished chemotherapy on average over 2.5 years before recruitment. The two studies evaluating MBI vs waitlist differed with respect to study objective and size (n = 26 feasibility study vs n =68 intervention comparison), as well as level of education (13.6 vs 15 years) and time since chemotherapy completion (19 vs 25 months). Potential concerns regarding the COG/EDU_GD_GRP vs waitlist comparison were not identified in semi-objective assessments of transitivity, possibly because some key effect modifiers were dropped due to incomplete reporting across all studies (i.e., depression, anxiety, fatigue).

As noted above, boxplots of the anticipated effect modifiers of clinical relevance were investigated. Plots for the learning outcome (immediate post-intervention) are provided below.

Box Plots for Learning Outcome (Immediate post-intervention)

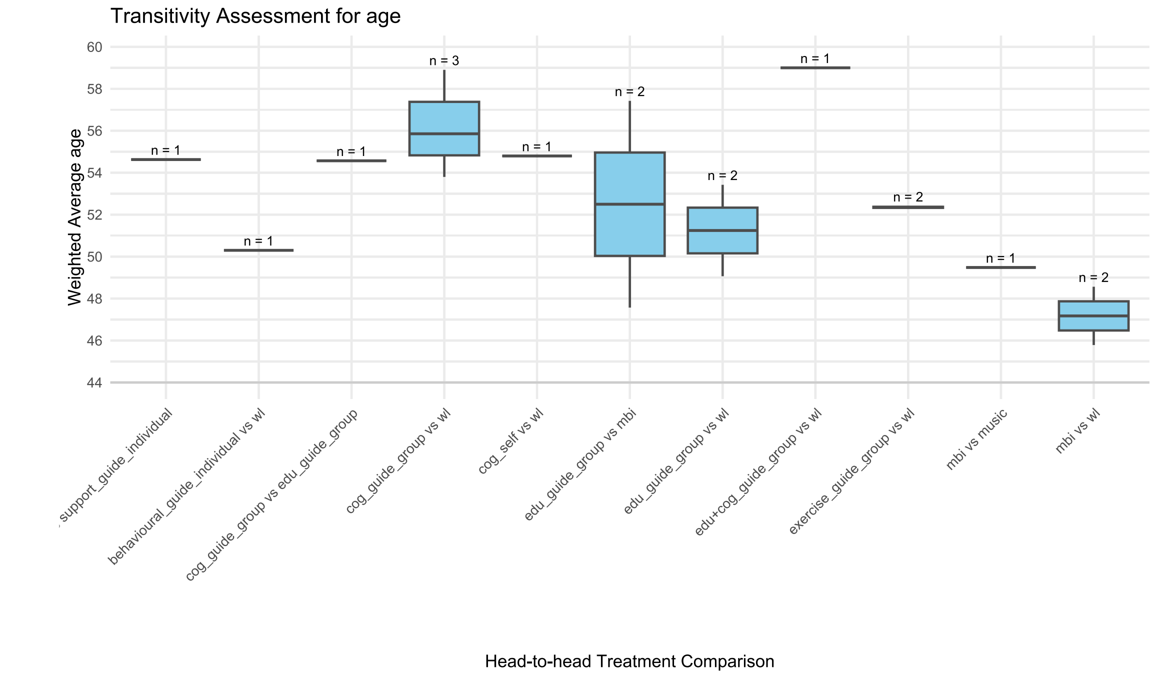

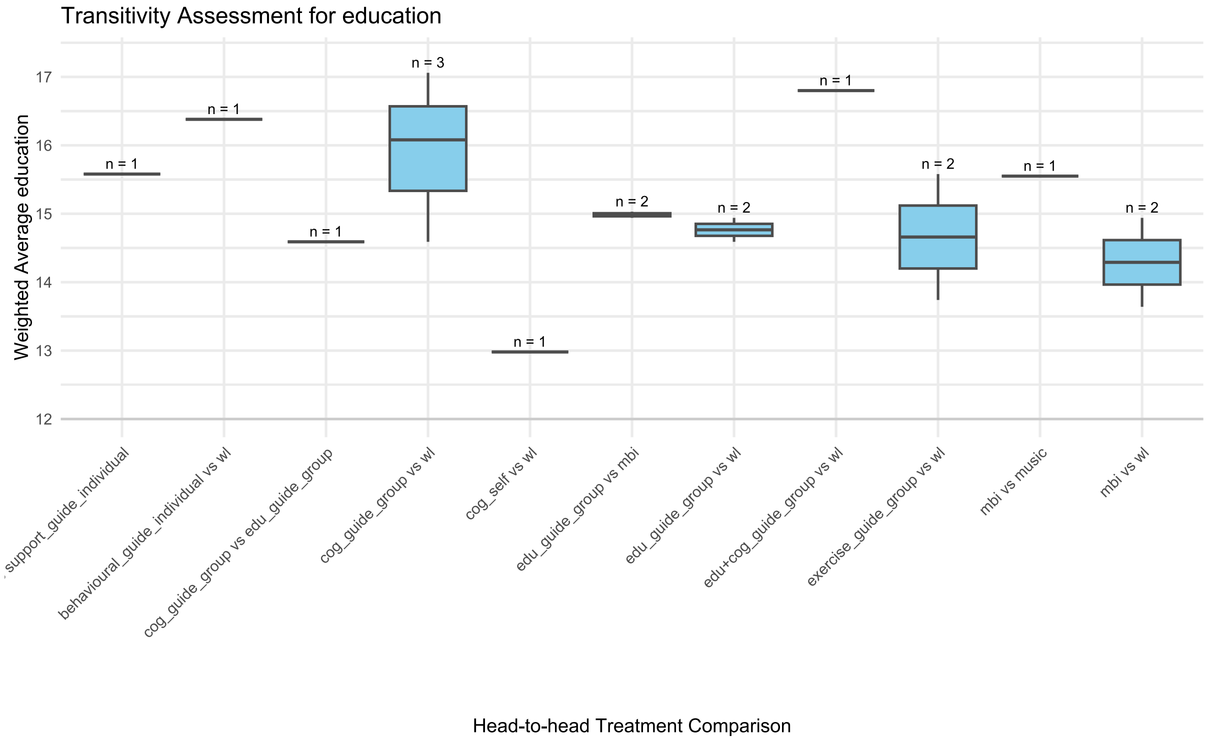

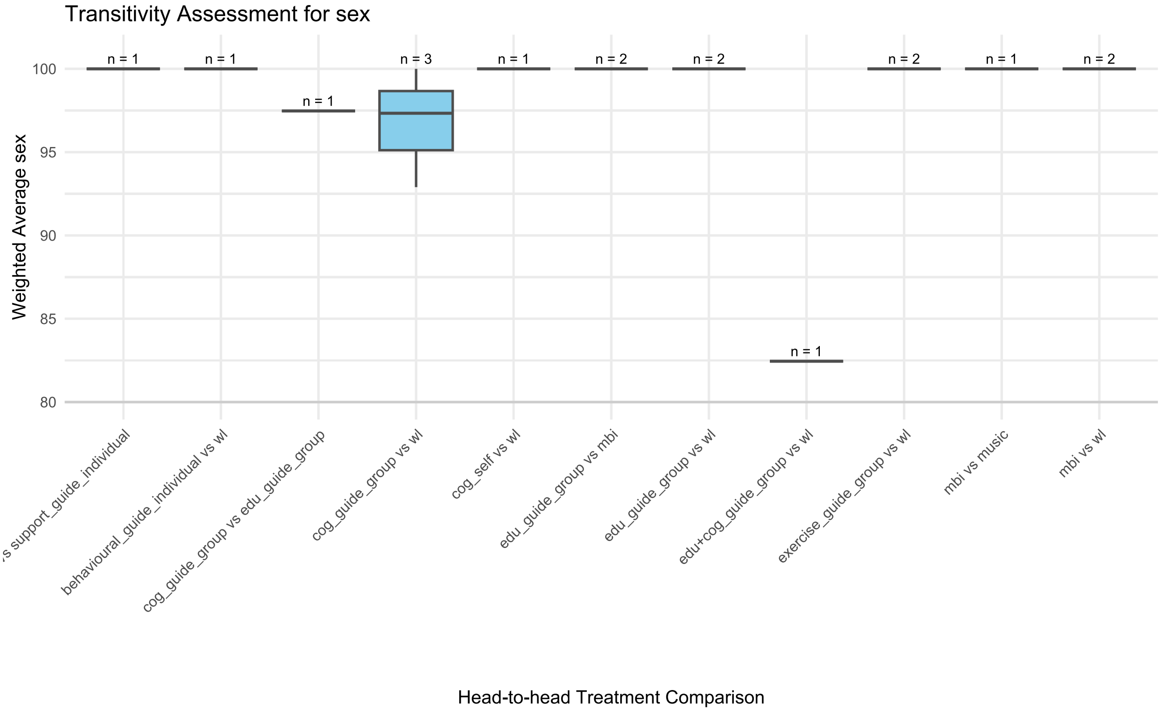
Transitivity Assessment for sex
Weighted Average sex
100
95
90
85
80
n = 1
n = 1
n = 1
n = 3
n = 1
n = 2
n = 2
n = 1
n = 2
n = 1
n = 2
support_guide_individual
behavioural_guide_individual vs wl
cog_guide_group vs edu_guide_group
cog_guide_group vs wl
cog_self vs wl
edu_guide_group vs mbi
edu_guide_group vs wl
edu+cog_guide_group vs wl
exercise_guide_group vs wl
mbi vs music
mbi vs wl
Head-to-head Treatment Comparison

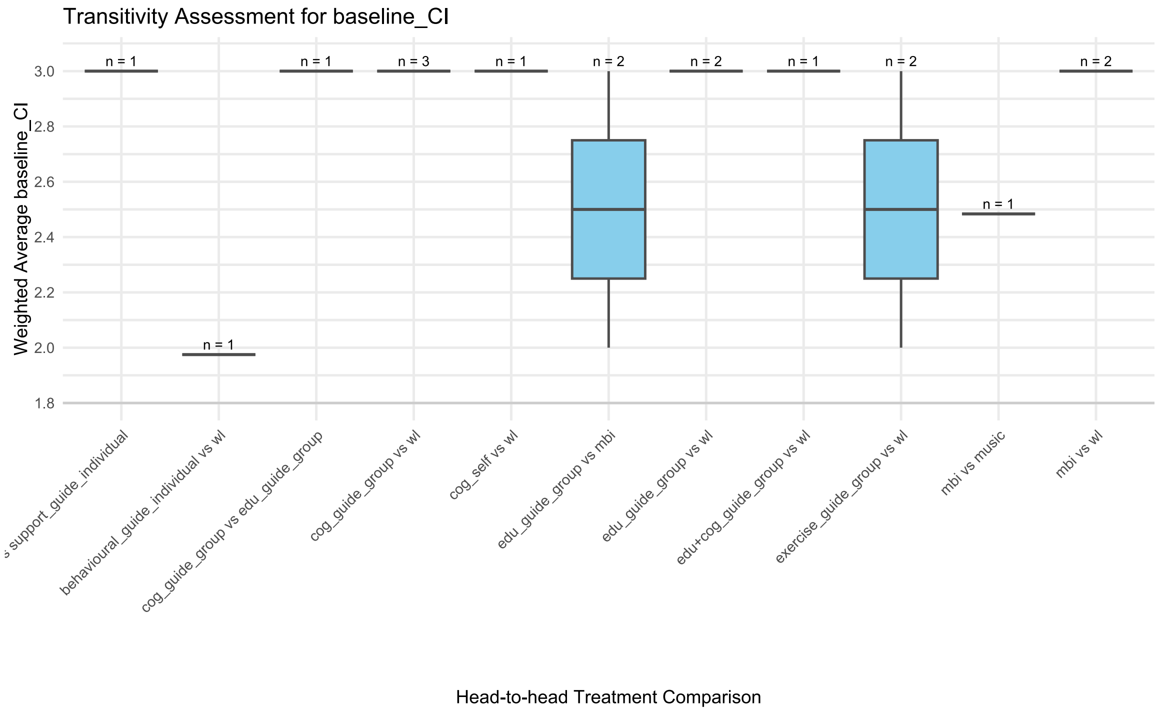
Transitivity Assessment for baseline_CI
Weighted Average baseline_CI
3.0
2.8
2.6
2.4
2.2
2.0
1.8
n = 1
n = 1
n = 1
n = 3
n = 1
n = 2
n = 2
n = 1
n = 2
n = 1
n = 2
support_guide_individual
behavioural_guide_individual vs wl
cog_guide_group vs edu_guide_group
cog_guide_group vs wl
cog_self vs wl
edu_guide_group vs mbi
edu_guide_group vs wl
edu+cog_guide_group vs wl
exercise_guide_group vs wl
mbi vs music
mbi vs wl
Head-to-head Treatment Comparison

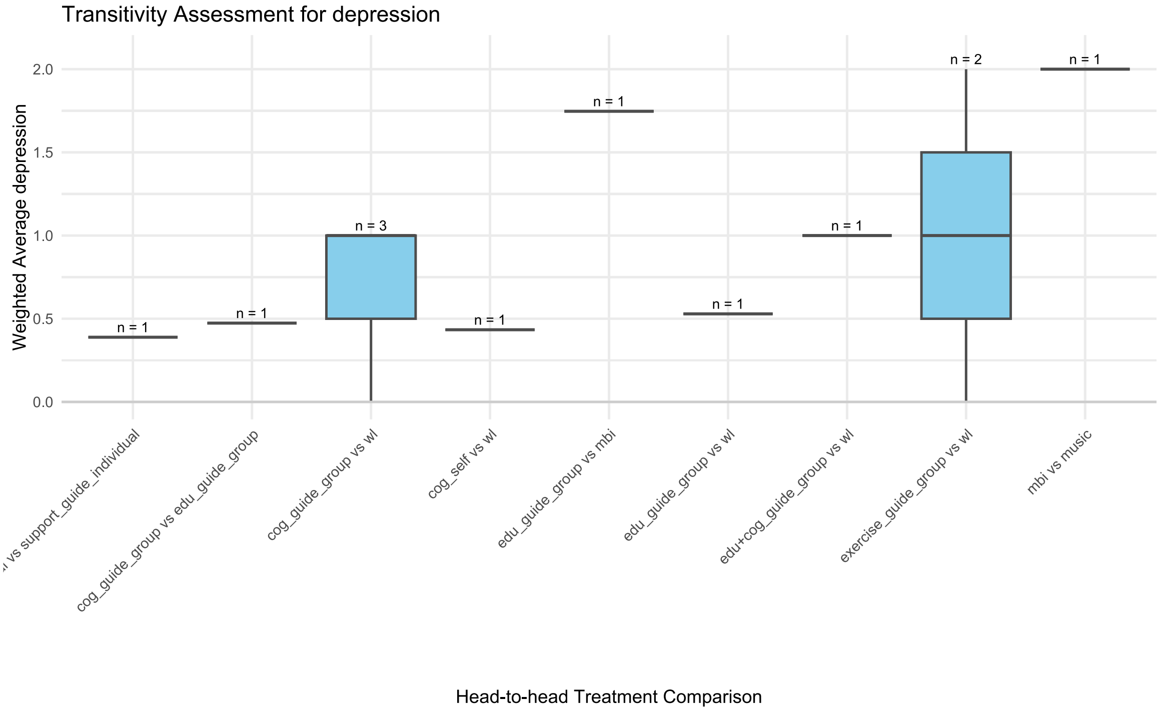
Transitivity Assessment for depression
Weighted Average depression
2.0
1.5
1.0
0.5
0.0
n = 1
n = 1
n = 3
n = 1
n = 1
n = 1
n = 1
n = 2
n = 1
vs support_guide_individual
cog_guide_group vs edu_guide_group
cog_guide_group vs wl
cog_self vs wl
edu_guide_group vs mbi
edu_guide_group vs wl
edu+cog_guide_group vs wl
exercise_guide_group vs wl
mbi vs music
Head-to-head Treatment Comparison

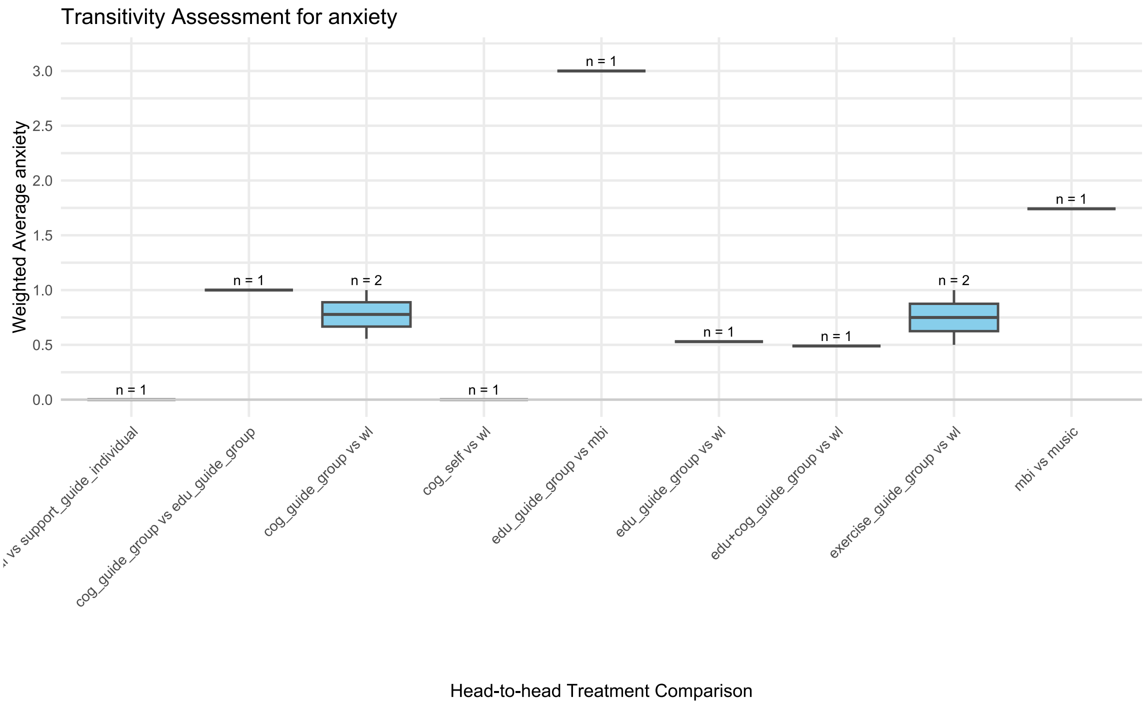
Transitivity Assessment for anxiety
Weighted Average anxiety
3.0
2.5
2.0
1.5
1.0
0.5
0.0
n = 1
n = 1
n = 2
n = 1
n = 1
n = 1
n = 1
n = 2
n = 1
vs support_guide_individual
cog_guide_group vs edu_guide_group
cog_guide_group vs wl
cog_self vs wl
edu_guide_group vs mbi
edu_guide_group vs wl
edu+cog_guide_group vs wl
exercise_guide_group vs wl
mbi vs music
Head-to-head Treatment Comparison

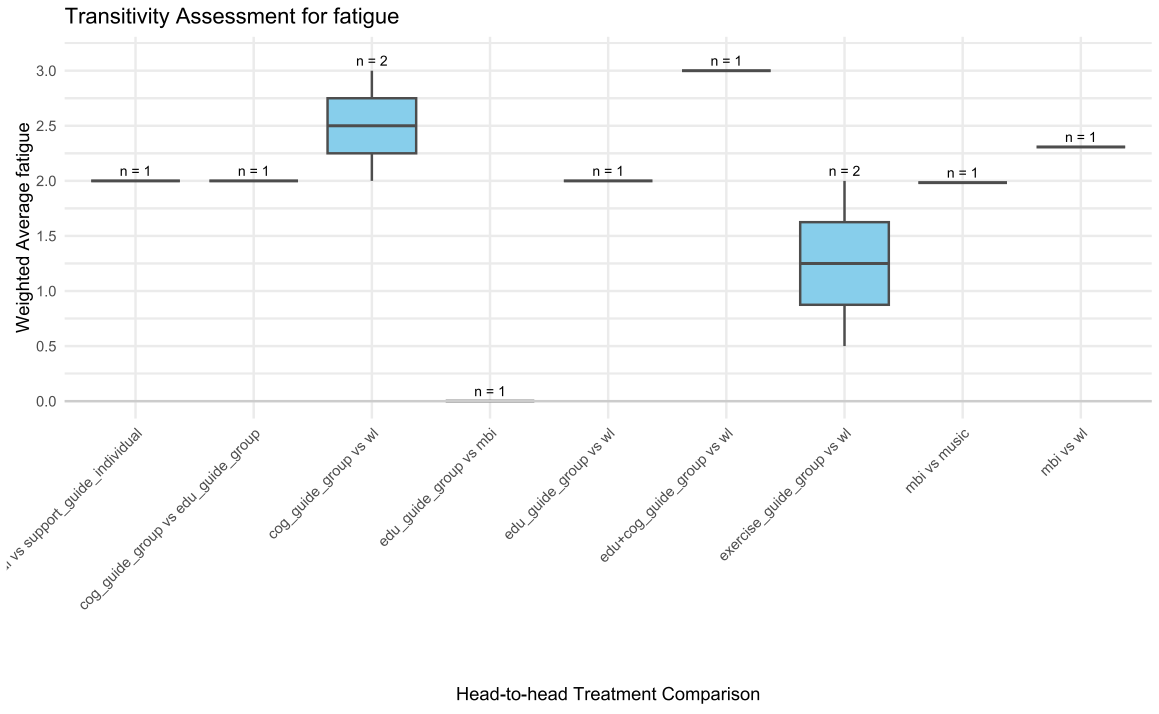
Transitivity Assessment for fatigue
Weighted Average fatigue
Head-to-head Treatment Comparison
n = 1
n = 2
cog_guide_group vs edu_guide_group
cog_guide_group vs wl
edu_guide_group vs mbi
edu_guide_group vs wl
edu+cog_guide_group vs wl
exercise_guide_group vs wl
mbi vs music
mbi vs wl

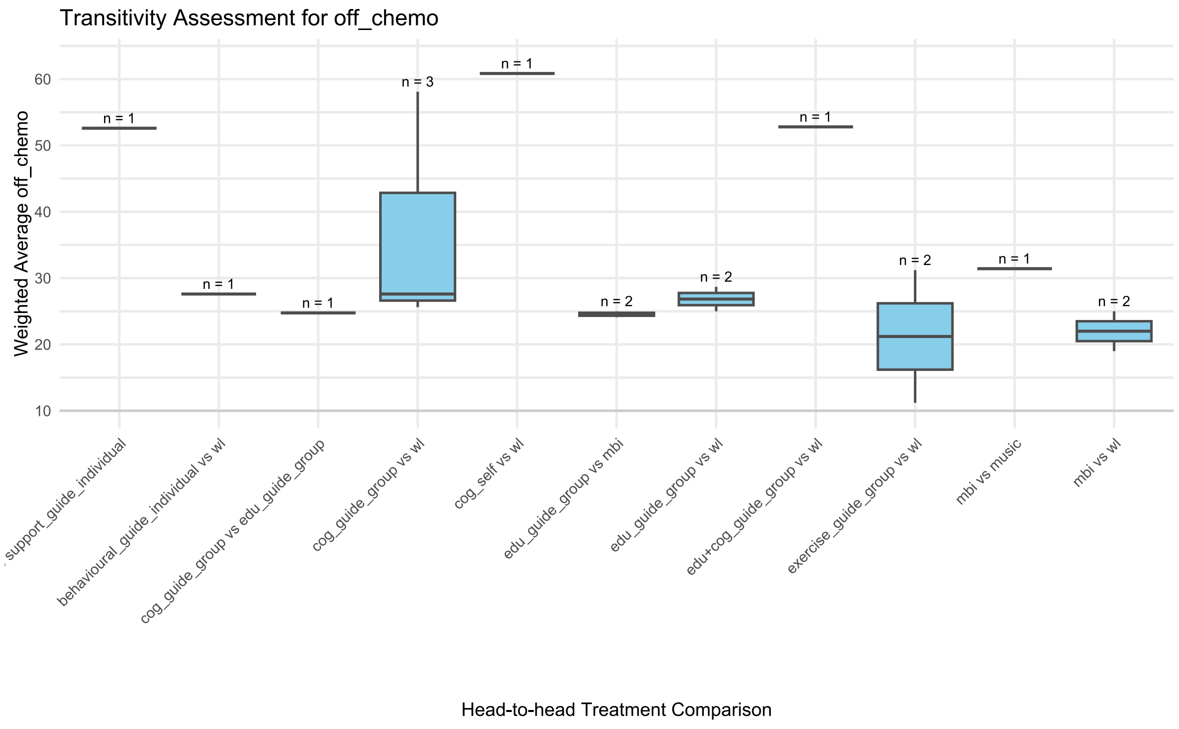
Transitivity Assessment for off_chemo
Weighted Average off_chemo
Head-to-head Treatment Comparison
n = 1
n = 2
n = 3
behavioural_guide_individual vs wl
cog_guide_group vs edu_guide_group
cog_guide_group vs wl
cog_self vs wl
edu_guide_group vs mbi
edu_guide_group vs wl
edu+cog_guide_group vs wl
exercise_guide_group vs wl
mbi vs music
mbi vs wl

File S7: Study-level risk of bias appraisals

Overall appraisal is for any objective cognitive function outcome. Incomplete outcome data was assessed to be at High risk of bias if there was >15% attrition rate and it was unbalanced between groups. “Other” sources of bias considered were (1) baseline imbalances between groups for key effect modifiers and (2) industry funding sources. Regarding baseline imbalances, we assessed bias of the raw data that we extracted not the bias of the results reported in the study. If adjusted findings were reported by study authors because there were imbalances at baseline between groups, we still assigned High ROB because the raw data that we used were not adjusted. The overall appraisal was “Low” if all judgments for a study were low, “High” if one or more judgments were high, and “Unclear” for all other combinations. However, if only the two blinding domain judgments were high, the overall appraisal was Unclear, unless the test to measure objective cognitive function was interpreted by a human, in which case, the overall appraisal was High, if the outcome assessors weren’t blinded. Detailed explanations of the judgments are available upon request.

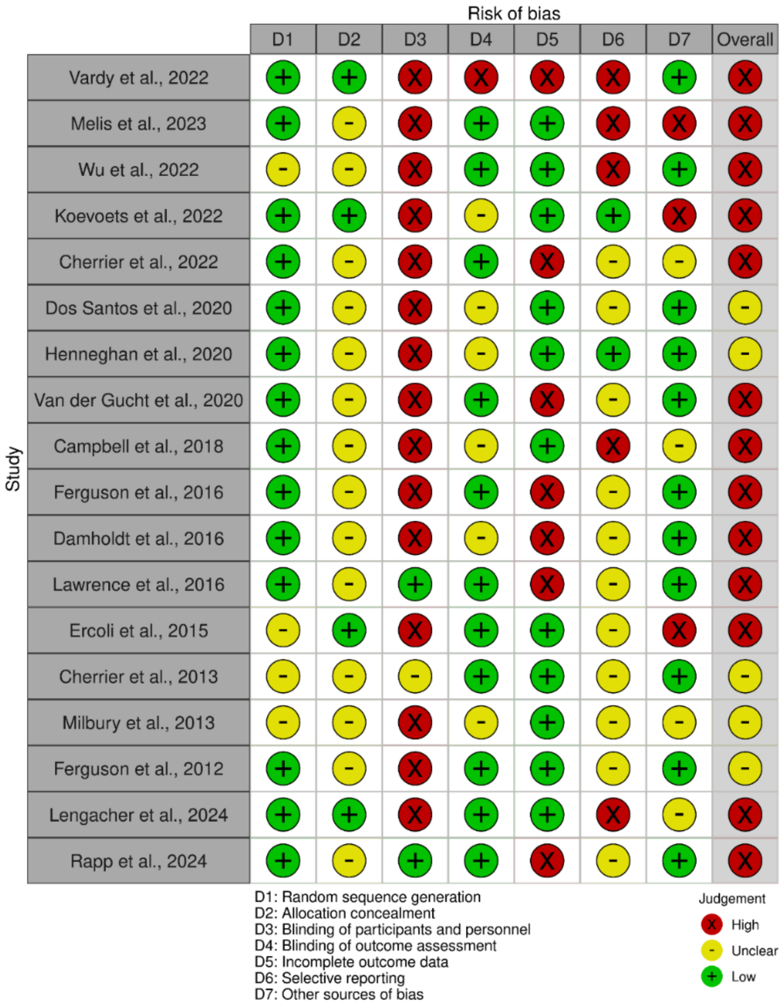

File S8: Network meta-analysis: supporting statistical data from immediate post-intervention analyses and certainty of evidence (CINeMA) appraisals

Primary network meta-analyses immediate post-intervention: findings for all treatment comparisons

League tables were generated for each outcome, demonstrating all direct (above the diagonal) and NMA (below the diagonal) estimates for all treatment comparisons. Effect estimates are reported as standardized mean differences (SMDs) with 95% confidence intervals (CIs) and 95% prediction intervals (PIs) below. Treatments are ordered by P-score, which is reported on the diagonal, with significant effects in bold text and highlighted in orange. Comparisons highlighted in red were found to have 95% CIs and PIs that differed in relation to the null value (i.e., the 95% CI did not cross SMD = 0 and the 95% PI did), suggesting potential heterogeneity issues in that future trials may yield treatment effects that are smaller than observed or even null, despite the current meta-analytic evidence indicating a statistically significant effect.

Learning

Treatment	COG/EDU_GD_GRP	SUP_GD_IND	BT_GD_IND	COG_GD_GRP	COG_SELF_IND	MBI	EDU_GD_GRP	EXE_GD_GRP	WL	MUSIC
**COG/EDU_GD_GRP**	**0.95**	.	.	.	.	.	.	.	**0.88<br>(0.46 to 1.29)**	.
**SUP_GD_IND**	0.17<br>(-0.84 to 1.19)<br>(-1.09 to 1.44)	**0.82**	0.36<br>(-0.31 to 1.04)	.	.	.	.	.	.	.
**BT_GD_IND**	0.54<br>(-0.21 to 1.29)<br>(-0.40 to 1.48)	0.36<br>(-0.31 to 1.04)<br>(-0.48 to 1.21)	**0.61**	.	.	.	.	.	0.34<br>(-0.29 to 0.96)	.
**COG_GD_GRP**	**0.66<br>(0.10 to 1.22)<br>(-0.04 to 1.36)**	0.48<br>(-0.51 to 1.48)<br>(-0.75 to 1.72)	0.12<br>(-0.61 to 0.85)<br>(-0.79 to 1.03)	**0.54**	.	.	-0.27<br>(-0.91 to 0.37)	.	0.26<br>(-0.13 to 0.65)	.
**COG_SELF_IND**	**0.69<br>(0.15 to 1.23)<br>(0.02 to 1.36)**	0.52<br>(-0.46 to 1.50)<br>(-0.71 to 1.74)	0.15<br>(-0.56 to 0.86)<br>(-0.74 to 1.04)	0.03<br>(-0.47 to 0.54)<br>(-0.60 to 0.66)	**0.50**	.	.	.	0.19<br>(-0.15 to 0.53)	.
**MBI**	**0.75<br>(0.21 to 1.29)<br>(0.07 to 1.42)**	0.57<br>(-0.41 to 1.56)<br>(-0.65 to 1.80)	0.21<br>(-0.50 to 0.92)<br>(-0.68 to 1.10)	0.09<br>(-0.38 to 0.56)<br>(-0.49 to 0.67)	0.06<br>(-0.42 to 0.54)<br>(-0.54 to 0.66)	**0.43**	0.02<br>(-0.24 to 0.28)	.	0.06<br>(-0.34 to 0.47)	0.26<br>(-0.45 to 0.97)
**EDU_GD_GRP**	**0.75<br>(0.22 to 1.29)<br>(0.08 to 1.43)**	0.58<br>(-0.40 to 1.56)<br>(-0.64 to 1.80)	0.22<br>(-0.49 to 0.93)<br>(-0.67 to 1.10)	0.09<br>(-0.35 to 0.54)<br>(-0.46 to 0.65)	0.06<br>(-0.42 to 0.54)<br>(-0.53 to 0.66)	0.01<br>(-0.25 to 0.26)<br>(-0.31 to 0.32)	**0.42**	.	0.03<br>(-0.36 to 0.43)	.
**EXE_GD_GRP**	**0.86<br>(0.35 to 1.37)<br>(0.22 to 1.50)**	0.68<br>(-0.28 to 1.65)<br>(-0.52 to 1.89)	0.32<br>(-0.37 to 1.01)<br>(-0.54 to 1.18)	0.20<br>(-0.27 to 0.67)<br>(-0.39 to 0.79)	0.17<br>(-0.28 to 0.62)<br>(-0.39 to 0.73)	0.11<br>(-0.34 to 0.56)<br>(-0.45 to 0.67)	0.11<br>(-0.34 to 0.55)<br>(-0.45 to 0.66)	**0.28**	0.02<br>(-0.27 to 0.31)	.
**WL**	**0.88<br>(0.46 to 1.29)<br>(0.35 to 1.40)**	0.70<br>(-0.22 to 1.62)<br>(-0.45 to 1.85)	0.34<br>(-0.29 to 0.96)<br>(-0.44 to 1.12)	0.22<br>(-0.16 to 0.59)<br>(-0.25 to 0.68)	0.19<br>(-0.15 to 0.53)<br>(-0.24 to 0.61)	0.13<br>(-0.21 to 0.47)<br>(-0.30 to 0.56)	0.12<br>(-0.22 to 0.46)<br>(-0.30 to 0.54)	0.02<br>(-0.27 to 0.31)<br>(-0.35 to 0.38)	**0.23**	.
**MUSIC**	**1.01<br>(0.12 to 1.90)<br>(-0.10 to 2.12)**	0.83<br>(-0.38 to 2.04)<br>(-0.68 to 2.34)	0.47<br>(-0.53 to 1.47)<br>(-0.78 to 1.72)	0.35<br>(-0.50 to 1.20)<br>(-0.71 to 1.41)	0.32<br>(-0.54 to 1.17)<br>(-0.75 to 1.39)	0.26<br>(-0.45 to 0.97)<br>(-0.62 to 1.14)	0.25<br>(-0.50 to 1.00)<br>(-0.68 to 1.19)	0.15<br>(-0.69 to 0.99)<br>(-0.90 to 1.19)	0.13<br>(-0.65 to 0.92)<br>(-0.85 to 1.11)	**0.22**

Memory

Treatment	COG/EDU_GD_GRP	COG_GD_GRP	MBI	EDU_GD_GRP	COG_SELF_IND	WL	MUSIC	EXE_GD_GRP
COG/EDU_GD_GRP	**0.92**	.	.	.	.	**0.54 (0.14 to 0.95)**	.	.
COG_GD_GRP	0.21 (-0.35 to 0.76) (-0.48 to 0.90)	**0.77**	.	-0.17 (-0.81 to 0.47)	.	0.39 (0.00 to 0.78)	.	.
MBI	0.32 (-0.22 to 0.85) (-0.35 to 0.98)	0.11 (-0.36 to 0.58) (-0.47 to 0.69)	**0.66**	0.07 (-0.19 to 0.34)	.	0.09 (-0.31 to 0.50)	0.43 (-0.28 to 1.14)	.
EDU_GD_GRP	0.38 (-0.15 to 0.91) (-0.28 to 1.04)	0.17 (-0.28 to 0.62) (-0.39 to 0.73)	0.06 (-0.19 to 0.31) (-0.25 to 0.38)	**0.56**	.	0.13 (-0.27 to 0.53)	.	.
COG_SELF_IND	0.49 (-0.04 to 1.02) (-0.18 to 1.15)	0.28 (-0.22 to 0.78) (-0.35 to 0.91)	0.17 (-0.31 to 0.65) (-0.43 to 0.77)	0.11 (-0.37 to 0.59) (-0.49 to 0.71)	**0.42**	0.06 (-0.28 to 0.40)	.	.
WL	**0.54 (0.14 to 0.95) (0.03 to 1.05)**	0.34 (-0.04 to 0.71) (-0.13 to 0.80)	0.23 (-0.12 to 0.57) (-0.20 to 0.66)	0.17 (-0.17 to 0.50) (-0.26 to 0.59)	0.06 (-0.28 to 0.40) (-0.37 to 0.48)	**0.33**	.	0.19 (-0.10 to 0.49)
MUSIC	0.75 (-0.14 to 1.64) (-0.36 to 1.86)	0.54 (-0.31 to 1.39) (-0.52 to 1.60)	0.43 (-0.28 to 1.14) (-0.46 to 1.32)	0.37 (-0.39 to 1.13) (-0.57 to 1.31)	0.26 (-0.60 to 1.12) (-0.81 to 1.33)	0.20 (-0.59 to 1.00) (-0.78 to 1.19)	**0.22**	.
EXE_GD_GRP	**0.74 (0.24 to 1.24) (0.11 to 1.36)**	**0.53 (0.06 to 1.00) (-0.06 to 1.12)**	0.42 (-0.03 to 0.87) (-0.14 to 0.98)	0.36 (-0.09 to 0.81) (-0.20 to 0.92)	0.25 (-0.20 to 0.70) (-0.31 to 0.81)	0.19 (-0.10 to 0.49) (-0.17 to 0.56)	-0.01 (-0.85 to 0.83) (-1.06 to 1.04)	**0.12**

Processing speed

Treatment	COG/EDU_GD_GRP	MUSIC	MBI	BT_GD_IND	EDU_GD_GRP	SUP_GD_IND	COG_GD_GRP	WL
**COG/EDU_GD_GRP**	**0.80**	.	.	.	.	.	.	**0.59 (0.18 to 1.00)**
**MUSIC**	-0.01 (-0.89 to 0.88) (-1.17 to 1.15)	**0.76**	0.14 (-0.57 to 0.84)	.	.	.	.	.
**MBI**	0.13 (-0.40 to 0.67) (-0.57 to 0.83)	0.14 (-0.57 to 0.84) (-0.79 to 1.06)	**0.71**	.	0.14 (-0.12 to 0.40)	.	.	0.41 (0.00 to 0.81)
**BT_GD_IND**	0.21 (-0.54 to 0.96) (-0.77 to 1.19)	0.22 (-0.79 to 1.22) (-1.10 to 1.53)	0.08 (-0.64 to 0.79) (-0.86 to 1.02)	**0.61**	.	0.50 (-0.18 to 1.18)	.	0.38 (-0.24 to 1.01)
**EDU_GD_GRP**	0.26 (-0.27 to 0.79) (-0.44 to 0.96)	0.27 (-0.48 to 1.02) (-0.71 to 1.25)	0.13 (-0.12 to 0.38) (-0.20 to 0.46)	0.05 (-0.66 to 0.76) (-0.88 to 0.99)	**0.53**	.	0.44 (-0.20 to 1.08)	0.38 (-0.02 to 0.78)
**SUP_GD_IND**	0.71 (-0.30 to 1.72) (-0.62 to 2.03)	0.72 (-0.50 to 1.93) (-0.87 to 2.31)	0.58 (-0.41 to 1.56) (-0.72 to 1.87)	0.50 (-0.18 to 1.18) (-0.39 to 1.39)	0.45 (-0.54 to 1.43) (-0.84 to 1.74)	**0.20**	.	.
**COG_GD_GRP**	**0.61 (0.06 to 1.16) (-0.11 to 1.33)**	0.62 (-0.23 to 1.46) (-0.49 to 1.73)	**0.48 (0.01 to 0.94) (-0.13 to 1.09)**	0.40 (-0.33 to 1.13) (-0.55 to 1.35)	0.35 (-0.10 to 0.80) (-0.24 to 0.94)	-0.10 (-1.09 to 0.90) (-1.40 to 1.21)	**0.19**	-0.02 (-0.41 to 0.36)
**WL**	**0.59 (0.18 to 1.00) (0.06 to 1.13)**	0.60 (-0.19 to 1.38) (-0.43 to 1.63)	**0.46 (0.11 to 0.80) (0.01 to 0.91)**	0.38 (-0.24 to 1.01) (-0.44 to 1.20)	0.33 (-0.01 to 0.67) (-0.11 to 0.78)	-0.12 (-1.04 to 0.81) (-1.33 to 1.10)	-0.02 (-0.39 to 0.35) (-0.50 to 0.47)	**0.19**

Word generation

Treatment	EXE_GD_GRP	MUSIC	MBI	COG_GD_IND	COG_SELF_IND	WL	EDU_GD_GRP	COG_GD_GRP
**EXE_GD_GRP**	**0.87**	.	.	.	.	0.75 (-0.18 to 1.68)	.	.
**MUSIC**	0.27 (-0.94 to 1.49) (-1.32 to 1.87)	**0.75**	0.17 (-0.54 to 0.87)	.	.	.	.	.
**MBI**	0.44 (-0.55 to 1.43) (-0.86 to 1.74)	0.17 (-0.54 to 0.87) (-0.76 to 1.09)	**0.71**	.	.	0.18 (-0.23 to 0.59)	**0.40 (0.14 to 0.66)**	.
**COG_GD_IND**	0.53 (-0.47 to 1.53) (-0.79 to 1.84)	0.25 (-0.62 to 1.12) (-0.89 to 1.39)	0.09 (-0.42 to 0.59) (-0.58 to 0.75)	**0.61**	0.26 (-0.15 to 0.67)	0.15 (-0.25 to 0.54)	.	.
**COG_SELF_IND**	0.70 (-0.26 to 1.67) (-0.57 to 1.97)	0.43 (-0.40 to 1.26) (-0.66 to 1.51)	0.26 (-0.17 to 0.69) (-0.30 to 0.83)	0.18 (-0.20 to 0.55) (-0.32 to 0.67)	**0.37**	0.05 (-0.21 to 0.31)	.	.
**WL**	0.75 (-0.18 to 1.68) (-0.47 to 1.97)	0.47 (-0.31 to 1.26) (-0.56 to 1.50)	0.31 (-0.04 to 0.65) (-0.14 to 0.76)	0.22 (-0.15 to 0.59) (-0.26 to 0.70)	0.05 (-0.21 to 0.31) (-0.30 to 0.39)	**0.30**	0.10 (-0.30 to 0.49)	0.04 (-0.41 to 0.49)
**EDU_GD_GRP**	0.82 (-0.17 to 1.81) (-0.48 to 2.12)	0.55 (-0.20 to 1.30) (-0.44 to 1.53)	**0.38 (0.13 to 0.64) (0.05 to 0.72)**	0.30 (-0.21 to 0.80) (-0.36 to 0.95)	0.12 (-0.31 to 0.55) (-0.44 to 0.68)	0.07 (-0.27 to 0.41) (-0.37 to 0.52)	**0.21**	0.36 (-0.28 to 1.00)
**COG_GD_GRP**	0.87 (-0.15 to 1.90) (-0.47 to 2.21)	0.60 (-0.27 to 1.46) (-0.54 to 1.73)	0.43 (-0.07 to 0.93) (-0.22 to 1.09)	0.34 (-0.22 to 0.91) (-0.39 to 1.08)	0.17 (-0.33 to 0.67) (-0.48 to 0.82)	0.12 (-0.30 to 0.55) (-0.43 to 0.68)	0.05 (-0.43 to 0.53) (-0.58 to 0.68)	**0.18**

Cognitive flexibility

Treatment	COG/EDU_GD_GRP	EXE_GD_GRP	MUSIC	COG_GD_IND	MBI	COG_SELF_IND	COG_GD_GRP	EDU_GD_GRP	BT_GD_IND	WL
**COG/EDU_GD_GRP**	**0.74**	.	.	.	.	.	.	.	.	0.39<br>(-0.01 to 0.79)
**EXE_GD_GRP**	-0.11<br>(-1.11 to 0.89)<br>(-1.42 to 1.20)	**0.73**	.	.	.	.	.	.	.	0.50<br>(-0.41 to 1.42)
**MUSIC**	-0.05<br>(-0.94 to 0.83)<br>(-1.21 to 1.11)	0.06<br>(-1.15 to 1.26)<br>(-1.52 to 1.64)	**0.72**	.	0.28<br>(-0.43 to 0.99)	.	.	.	.	.
**COG_GD_IND**	0.09<br>(-0.48 to 0.65)<br>(-0.66 to 0.83)	0.20<br>(-0.80 to 1.19)<br>(-1.11 to 1.50)	0.14<br>(-0.74 to 1.02)<br>(-1.02 to 1.29)	**0.66**	.	0.14<br>(-0.27 to 0.55)	.	.	.	0.31<br>(-0.09 to 0.70)
**MBI**	0.23<br>(-0.30 to 0.76)<br>(-0.47 to 0.92)	0.34<br>(-0.64 to 1.31)<br>(-0.94 to 1.62)	0.28<br>(-0.43 to 0.99)<br>(-0.65 to 1.21)	0.14<br>(-0.38 to 0.66)<br>(-0.55 to 0.83)	**0.50**	.	.	0.09<br>(-0.17 to 0.35)	.	0.15<br>(-0.25 to 0.56)
**COG_SELF_IND**	0.22<br>(-0.35 to 0.80)<br>(-0.53 to 0.97)	0.34<br>(-0.66 to 1.34)<br>(-0.98 to 1.65)	0.28<br>(-0.61 to 1.16)<br>(-0.88 to 1.44)	0.14<br>(-0.27 to 0.55)<br>(-0.40 to 0.68)	-0.00<br>(-0.53 to 0.53)<br>(-0.70 to 0.69)	**0.48**	.	.	.	0.17<br>(-0.24 to 0.57)
**COG_GD_GRP**	0.34<br>(-0.20 to 0.89)<br>(-0.37 to 1.06)	0.46<br>(-0.53 to 1.44)<br>(-0.84 to 1.75)	0.40<br>(-0.45 to 1.24)<br>(-0.71 to 1.51)	0.26<br>(-0.28 to 0.80)<br>(-0.45 to 0.97)	0.12<br>(-0.35 to 0.58)<br>(-0.49 to 0.73)	0.12<br>(-0.43 to 0.67)<br>(-0.60 to 0.84)	**0.33**	0.07<br>(-0.56 to 0.71)	.	0.01<br>(-0.37 to 0.40)
**EDU_GD_GRP**	0.33<br>(-0.19 to 0.86)<br>(-0.36 to 1.02)	0.44<br>(-0.53 to 1.42)<br>(-0.83 to 1.72)	0.39<br>(-0.37 to 1.14)<br>(-0.60 to 1.37)	0.25<br>(-0.27 to 0.77)<br>(-0.44 to 0.93)	0.11<br>(-0.15 to 0.36)<br>(-0.22 to 0.44)	0.11<br>(-0.42 to 0.63)<br>(-0.58 to 0.80)	-0.01<br>(-0.46 to 0.43)<br>(-0.60 to 0.57)	**0.33**	.	0.12<br>(-0.28 to 0.52)
**BT_GD_IND**	0.43<br>(-0.31 to 1.17)<br>(-0.54 to 1.40)	0.54<br>(-0.57 to 1.65)<br>(-0.91 to 1.99)	0.48<br>(-0.52 to 1.48)<br>(-0.83 to 1.80)	0.34<br>(-0.39 to 1.08)<br>(-0.62 to 1.31)	0.20<br>(-0.51 to 0.91)<br>(-0.73 to 1.13)	0.20<br>(-0.54 to 0.94)<br>(-0.77 to 1.18)	0.08<br>(-0.64 to 0.81)<br>(-0.86 to 1.03)	0.10<br>(-0.61 to 0.80)<br>(-0.83 to 1.02)	**0.28**	-0.04<br>(-0.66 to 0.58)
**WL**	0.39<br>(-0.01 to 0.79)<br>(-0.14 to 0.92)	0.50<br>(-0.41 to 1.42)<br>(-0.70 to 1.70)	0.44<br>(-0.34 to 1.23)<br>(-0.59 to 1.48)	0.31<br>(-0.09 to 0.70)<br>(-0.21 to 0.82)	0.16<br>(-0.18 to 0.51)<br>(-0.29 to 0.61)	0.17<br>(-0.24 to 0.57)<br>(-0.36 to 0.70)	0.05<br>(-0.32 to 0.42)<br>(-0.44 to 0.53)	0.06<br>(-0.28 to 0.40)<br>(-0.38 to 0.50)	-0.04<br>(-0.66 to 0.58)<br>(-0.85 to 0.78)	**0.23**

Attention

Treatment	COG/EDU_GD_GRP	COG_GD_GRP	EDU_GD_GRP	COG_SELF_IND	COG_GD_IND	MBI	WL
COG/EDU_GD_GRP	**0.99**	.	.	.	.	.	**0.92 (0.50 to 1.34)**
COG_GD_GRP	0.49 (-0.13 to 1.12) (-0.33 to 1.32)	**0.68**	-0.04 (-0.68 to 0.59)	.	.	.	0.48 (-0.03 to 0.98)
EDU_GD_GRP	**0.68 (0.14 to 1.22) (-0.03 to 1.39)**	0.18 (-0.32 to 0.69) (-0.48 to 0.84)	**0.48**	.	.	0.04 (-0.22 to 0.30)	0.26 (-0.14 to 0.66)
COG_SELF_IND	**0.69 (0.20 to 1.19) (0.04 to 1.34)**	0.20 (-0.34 to 0.73) (-0.50 to 0.90)	0.01 (-0.42 to 0.44) (-0.55 to 0.58)	**0.46**	-0.06 (-0.47 to 0.35)	.	0.23 (-0.03 to 0.49)
COG_GD_IND	**0.69 (0.13 to 1.25) (-0.05 to 1.42)**	0.19 (-0.40 to 0.79) (-0.59 to 0.97)	0.01 (-0.49 to 0.51) (-0.65 to 0.67)	-0.00 (-0.38 to 0.37) (-0.50 to 0.49)	**0.45**	.	0.18 (-0.21 to 0.58)
MBI	**0.73 (0.19 to 1.28) (0.02 to 1.45)**	0.24 (-0.29 to 0.76) (-0.45 to 0.93)	0.05 (-0.20 to 0.31) (-0.28 to 0.38)	0.04 (-0.39 to 0.47) (-0.53 to 0.61)	0.04 (-0.46 to 0.55) (-0.62 to 0.71)	**0.37**	0.08 (-0.32 to 0.49)
WL	**0.92 (0.50 to 1.34) (0.37 to 1.47)**	0.42 (-0.04 to 0.89) (-0.19 to 1.04)	0.24 (-0.10 to 0.58) (-0.21 to 0.69)	0.23 (-0.03 to 0.49) (-0.12 to 0.57)	0.23 (-0.14 to 0.60) (-0.25 to 0.71)	0.19 (-0.16 to 0.53) (-0.27 to 0.64)	**0.07**

Working memory

Treatment	COG/EDU_GD_GRP	COG_GD_GRP	COG_GD_IND	COG_SELF_IND	MBI	EDU_GD_GRP	WL
COG/EDU_GD_GRP	**0.87**	.	.	.	.	.	**0.74 (0.19 to 1.30)**
COG_GD_GRP	0.26 (-0.45 to 0.96) (-0.79 to 1.30)	**0.67**	.	.	.	0.33 (-0.41 to 1.07)	0.43 (-0.02 to 0.88)
COG_GD_IND	0.27 (-0.48 to 1.02) (-0.83 to 1.37)	0.01 (-0.65 to 0.68) (-0.99 to 1.01)	**0.64**	0.11 (-0.44 to 0.66)	.	.	0.49 (-0.05 to 1.04)
COG_SELF_IND	0.40 (-0.26 to 1.07) (-0.60 to 1.41)	0.15 (-0.43 to 0.72) (-0.75 to 1.04)	0.13 (-0.38 to 0.64) (-0.70 to 0.96)	**0.49**	.	.	0.34 (-0.03 to 0.71)
MBI	0.47 (-0.26 to 1.20) (-0.60 to 1.55)	0.21 (-0.40 to 0.83) (-0.73 to 1.16)	0.20 (-0.50 to 0.90) (-0.83 to 1.23)	0.07 (-0.54 to 0.67) (-0.86 to 1.00)	**0.41**	-0.16 (-0.77 to 0.45)	0.33 (-0.16 to 0.83)
EDU_GD_GRP	0.51 (-0.20 to 1.23) (-0.54 to 1.57)	0.26 (-0.29 to 0.80) (-0.61 to 1.12)	0.24 (-0.44 to 0.92) (-0.77 to 1.26)	0.11 (-0.47 to 0.69) (-0.80 to 1.02)	0.04 (-0.49 to 0.57) (-0.81 to 0.89)	**0.36**	0.13 (-0.35 to 0.61)
WL	**0.74 (0.19 to 1.30) (-0.13 to 1.62)**	**0.48 (0.05 to 0.92) (-0.26 to 1.23)**	0.47 (-0.04 to 0.98) (-0.35 to 1.29)	0.34 (-0.03 to 0.71) (-0.35 to 1.03)	0.27 (-0.21 to 0.75) (-0.52 to 1.06)	0.23 (-0.22 to 0.68) (-0.54 to 0.99)	**0.06**

Explorations of heterogeneity

Single arms of two three-arm trials[15,19] demonstrated substantial impact on heterogeneity estimates. The waitlist control arm of a three-arm trial was demonstrated to add substantial statistical heterogeneity across all outcomes (see table below)[19]. The participants in this arm performed significantly better at baseline than those of other arms for multiple

neuropsychological tests, resulting in a higher likelihood of better scores post-intervention for the control group. We could not identify a specific clinical source for this baseline heterogeneity, although the control group was slightly older, may have been less likely to have had anthracycline chemotherapy, was significantly more likely to be unemployed (not retired) than the educational group, and had lower income than the MBI group. Given its substantial impact on statistical heterogeneity, this arm was removed from all NMAs. In a second study[15], as per the study authors, an active control arm was added partway through the trial to satisfy ethics review. This educational control arm (EDU_GD_GRP) was substantially different from the active intervention (COG/EDU_GD_GRP) and waitlist control arms in terms of timing of randomization and key effect modifiers (fewer years since treatment, higher proportion had received chemotherapy), and a relatively much smaller sample size (n = 10 vs 49 and 47 in other arms). We elected to remove this small arm from NMAs due to clinical heterogeneity and methodological issues.

Regarding the processing speed outcome, effect estimates in preliminary analyses suggested unreliably large benefits for one intervention (exercise) and high SMDs for COG_GD_IND and COG_SELF_IND. We explored the data to understand these issues and identified two studies associated with these comparisons[16,20], both of which reported group-level change scores in lieu of raw data at follow-up. One was a very small study (n = 19), with substantial differences between groups in processing speed at baseline[16]. The second study reported no baseline processing speed data, with significant benefits for the waitlist control group over both COG_GD_IND and COG_SELF_IND. No explanation was provided by the study authors. Combining follow-up scores and change scores is only acceptable if there are no baseline differences across studies[21-23]. When these studies were removed, the effect estimates of the remaining comparisons were unchanged and waitlist was ranked as the lowest performing treatment. Because of baseline group differences and poor reporting, we removed these studies from the final analysis of processing speed. Other outcomes did not demonstrate statistical issues with change score data (see sensitivity analysis below).

Network meta-analysis: sensitivity analyses

In our protocol we planned to conduct meta-regressions, subgroup analyses, and/or sensitivity analyses on many potential sources of clinical and methodological heterogeneity, including follow-up times, study context, participant eligibility criteria (e.g., cancer type/stage, age, type of chemotherapy, use of selective serotonin reuptake inhibitors (SSRIs), confounding conditions such as fatigue), intervention characteristics, outcome definitions, and control group baseline risk. We conducted separate NMAs for immediate post-intervention and longest follow-up time; however, due to sparsity of networks and limited reporting of many covariates, other sensitivity analyses exploring the impact of potential effect modifiers could not be pursued.

In addition to the leave-one-out sensitivity analyses described above to explore heterogeneity, we also assessed the impact of including change scores (i.e., mean difference from baseline) that were reported in two studies[16,20] in lieu of follow-up raw data. Inclusion of both raw follow-up data and change scores is theoretically not advisable when NMA is conducted on the SMD scale[24]; however, recent empirical studies have demonstrated no difference between pooled estimates based on SMDs of follow-up data or change SMDs[21,22], except potentially for smaller trials with baseline imbalances[22]. Removal of change scores from our NMAs did not impact estimates of statistical heterogeneity (see table below) or intervention effects, except for the processing speed outcome, as described above.

Publication bias

Publication bias could not be assessed due to data sparseness: no comparison within any of the networks was informed by more than three trials, precluding publication bias assessment.**Table. Impact of sensitivity analyses on statistical heterogeneity**

Yellow highlight denotes analyses where $I^2 > 0$; green highlight denotes analyses where $I^2 = 0$.

Outcome/ Analysis	Learning	Memory	Processing speed	Word generation	Cognitive flexibility	Attention	Working memory
**All data**	tau^2 = 0.0065<br>tau = 0.0808<br>I^2 = 7.1% (0.0% to 67.3%)	tau^2 = 0.0123<br>tau = 0.1110<br>I^2 = 12.6% (0.0% to 54.5%)	tau^2 = 0.0589<br>tau = 0.2426<br>I^2 = 41.9% (0.0% to 74.3%)	tau^2 = 0<br>tau = 0<br>I^2 = 0% (0.0% to 70.8%)	tau^2 = 0<br>tau = 0<br>I^2 = 0% (0.0% to 67.6%)	tau^2 = 0<br>tau = 0<br>I^2 = 0% (0.0% to 67.6%)	tau^2 = 0.0316<br>tau = 0.1777<br>I^2 = 27.7% (0% to 68.8%)
**No Lengacher waitlist control**	tau^2 = 0<br>tau = 0<br>I^2 = 0% (0.0% to 67.6%)	tau^2 = 0<br>tau = 0<br>I^2 = 0% (0.0% to 67.6%)	tau^2 = 0<br>tau = 0<br>I^2 = 0% (0.0% to 70.8%)	tau^2 = 0<br>tau = 0<br>I^2 = 0% (0.0% to 74.6%)	tau^2 = 0<br>tau = 0<br>I^2 = 0% (0.0% to 70.8%)	tau^2 = 0<br>tau = 0<br>I^2 = 0% (0.0% to 70.8%)	No data removed (Lengacher not in the original dataset)
**FINAL data set: No Cherrier educational control OR Lengacher waitlist control**	tau^2 = 0<br>tau = 0<br>I^2 = 0% [0.0%; 70.8%]	tau^2 = 0<br>tau = 0<br>I^2 = 0% [0.0%; 70.8%]	tau^2 = 0<br>tau = 0<br>I^2 = 0% [0.0%; 74.6%]	tau^2 = 0<br>tau = 0<br>I^2 = 0% [0.0%; 74.6%]	tau^2 = 0<br>tau = 0<br>I^2 = 0% [0.0%; 74.6%]	tau^2 = 0<br>tau = 0<br>I^2 = 0% [0.0%; 74.6%]	tau^2 = 0.0357<br>tau = 0.1889<br>I^2 = 31.2% (0% to 72.1%)
**No change scores or Cherrier educational control OR Lengacher waitlist control**	tau^2 = 0<br>tau = 0<br>I^2 = 0% [0.0%; 74.6%]	tau^2 = 0<br>tau = 0<br>I^2 = 0% [0.0%; 74.6%]	tau^2 = 0<br>tau = 0<br>I^2 = 0% [0.0%; 74.6%]	tau^2 = 0<br>tau = 0<br>I^2 = 0% [0.0%; 79.2%]	tau^2 = 0<br>tau = 0<br>I^2 = 0% [0.0%; 74.6%]	tau^2 = 0<br>tau = 0<br>I^2 = 0% [0.0%; 79.2%]	tau^2 = 0.1091<br>tau = 0.3303<br>I^2 = 49.8% (0.0% to 81.6%)

Tests of inconsistency

Learning

No inconsistency was observed in the NMA globally (design-by-treatment interaction model: p = 0.408) or locally (node-splitting back-calculation method; see table below).

Comparison	Number of studies	Proportion of direct evidence	NMA effect SMD (95% CI)	Direct evidence effect SMD (95% CI)	Indirect evidence effect SMD (95% CI)	Difference between direct and indirect effects SMD (95% CI)	p-value (back-calculation method)
COG_GD_GRP vs WL	3	0.91	0.22 (-0.16 to 0.59)	0.26 (-0.13 to 0.65)	-0.22 (-1.48 to 1.04)	0.47 (-0.85 to 1.79)	0.481
EDU_GD_GRP vs MBI	2	0.93	-0.01 (-0.26 to 0.25)	-0.02 (-0.28 to 0.24)	0.23 (-0.73 to 1.18)	-0.25 (-1.24 to 0.74)	0.620
EDU_GD_GRP vs WL	2	0.72	0.12 (-0.22 to 0.46)	0.03 (-0.36 to 0.43)	0.34 (-0.29 to 0.97)	-0.31 (-1.06 to 0.44)	0.417
MBI vs WL	2	0.71	0.13 (-0.21 to 0.47)	0.06 (-0.34 to 0.47)	0.30 (-0.35 to 0.94)	-0.24 (-0.99 to 0.52)	0.543
COG_GD_GRP vs EDU_GD_GRP	1	0.49	0.09 (-0.35 to 0.54)	-0.27 (-0.91 to 0.37)	0.45 (-0.18 to 1.07)	-0.72 (-1.61 to 0.18)	0.117

Memory

No inconsistency was observed in the NMA globally (design-by-treatment interaction model: p = 0.378) or locally (node-splitting back-calculation method; see table below).

Comparison	Number of studies	Proportion of direct evidence	NMA effect SMD (95% CI)	Direct evidence effect SMD (95% CI)	Indirect evidence effect SMD (95% CI)	Difference between direct and indirect effects SMD (95% CI)	p-value (back-calculation method)
COG_GD_GRP vs WL	3	0.91	0.34 (-0.04 to 0.71)	0.39 (0.00 to 0.78)	-0.20 (-1.46 to 1.05)	0.59 (-0.72 to 1.91)	0.377
EDU_GD_GRP vs MBI	2	0.93	-0.06 (-0.31 to 0.19)	-0.07 (-0.34 to 0.19)	0.12 (-0.84 to 1.07)	-0.19 (-1.18 to 0.80)	0.708
EDU_GD_GRP vs WL	2	0.72	0.17 (-0.17 to 0.50)	0.13 (-0.27 to 0.53)	0.25 (-0.39 to 0.88)	-0.12 (-0.86 to 0.63)	0.763
MBI vs WL	2	0.71	0.23 (-0.12 to 0.57)	0.09 (-0.31 to 0.50)	0.56 (-0.08 to 1.20)	-0.46 (-1.23 to 0.30)	0.231
COG_GD_GRP vs EDU_GD_GRP	1	0.49	0.17 (-0.28 to 0.62)	-0.17 (-0.81 to 0.47)	0.50 (-0.13 to 1.13)	-0.67 (-1.57 to 0.22)	0.141

Processing speed

No inconsistency was observed in the NMA globally (design-by-treatment interaction model: p = 0.745) or locally (node-splitting back-calculation method; see table below).

Comparison	Number of studies	Proportion of direct evidence	NMA effect SMD (95% CI)	Direct evidence effect SMD (95% CI)	Indirect evidence effect SMD (95% CI)	Difference between direct and indirect effects SMD (95% CI)	p-value (back-calculation method)
COG_GD_GRP vs WL	3	0.92	-0.02 (-0.39 to 0.35)	-0.02 (-0.41 to 0.36)	0.03 (-1.24 to 1.31)	-0.06 (-1.39 to 1.28)	0.933
EDU_GD_GRP vs MBI	2	0.93	-0.13 (-0.38 to 0.12)	-0.14 (-0.40 to 0.12)	0.02 (-0.93 to 0.98)	-0.16 (-1.15 to 0.83)	0.749
EDU_GD_GRP vs WL	2	0.72	0.33 (-0.01 to 0.67)	0.38 (-0.02 to 0.78)	0.20 (-0.43 to 0.84)	0.18 (-0.57 to 0.93)	0.641
MBI vs WL	2	0.71	0.46 (0.11 to 0.80)	0.41 (0.00 to 0.81)	0.60 (-0.05 to 1.24)	-0.19 (-0.95 to 0.57)	0.625
COG_GD_GRP vs EDU_GD_GRP	1	0.49	-0.35 (-0.80 to 0.10)	-0.44 (-1.08 to 0.20)	-0.26 (-0.88 to 0.36)	-0.18 (-1.08 to 0.71)	0.690

Word generation

No inconsistency was observed in the NMA globally (design-by-treatment interaction model: p = 0.522) or locally (node-splitting back-calculation method; see table below).

Comparison	Number of studies	Proportion of direct evidence	NMA effect SMD (95% CI)	Direct evidence effect SMD (95% CI)	Indirect evidence effect SMD (95% CI)	Difference between direct and indirect effects SMD (95% CI)	p-value (back-calculation method)
COG_GD_GRP vs WL	2	0.89	-0.12 (-0.55 to 0.30)	-0.04 (-0.49 to 0.41)	-0.82 (-2.08 to 0.45)	0.78 (-0.57 to 2.13)	0.256
EDU_GD_GRP vs MBI	2	0.93	-0.38 (-0.64 to -0.13)	-0.40 (-0.66 to -0.14)	-0.12 (-1.10 to 0.85)	-0.28 (-1.28 to 0.73)	0.590
EDU_GD_GRP vs WL	2	0.73	-0.07 (-0.41 to 0.27)	-0.10 (-0.49 to 0.30)	-0.02 (-0.67 to 0.63)	-0.08 (-0.84 to 0.69)	0.841
MBI vs WL	2	0.72	0.31 (-0.04 to 0.65)	0.18 (-0.23 to 0.59)	0.64 (-0.01 to 1.29)	-0.46 (-1.23 to 0.31)	0.239
COG_GD_GRP vs EDU_GD_GRP	1	0.56	-0.05 (-0.53 to 0.43)	-0.36 (-1.00 to 0.28)	0.34 (-0.38 to 1.05)	-0.69 (-1.65 to 0.27)	0.158
COG_GD_IND vs COG_SELF_IND	1	0.84	0.18 (-0.20 to 0.55)	0.26 (-0.15 to 0.67)	-0.26 (-1.19 to 0.66)	0.52 (-0.49 to 1.53)	0.311
COG_GD_IND vs WL	1	0.87	0.22 (-0.15 to 0.59)	0.15 (-0.25 to 0.54)	0.71 (-0.31 to 1.73)	-0.56 (-1.66 to 0.53)	0.311

Cognitive flexibility

No inconsistency was observed in the NMA globally (design-by-treatment interaction model: p = 0.827) or locally (node-splitting back-calculation method; see table below).

Comparison	Number of studies	Proportion of direct evidence	NMA effect SMD (95% CI)	Direct evidence effect SMD (95% CI)	Indirect evidence effect SMD (95% CI)	Difference between direct and indirect effects SMD (95% CI)	p-value (back-calculation method)
COG_GD_GRP vs WL	3	0.91	0.05 (-0.32 to 0.42)	0.01 (-0.37 to 0.40)	0.44 (-0.82 to 1.69)	-0.43 (-1.74 to 0.88)	0.524
EDU_GD_GRP vs MBI	2	0.93	-0.11 (-0.36 to 0.15)	-0.09 (-0.35 to 0.17)	-0.28 (-1.24 to 0.67)	0.19 (-0.80 to 1.18)	0.706
EDU_GD_GRP vs WL	2	0.71	0.06 (-0.28 to 0.40)	0.12 (-0.28 to 0.52)	-0.09 (-0.72 to 0.54)	0.21 (-0.54 to 0.95)	0.587
MBI vs WL	2	0.72	0.16 (-0.18 to 0.51)	0.15 (-0.25 to 0.56)	0.20 (-0.44 to 0.84)	-0.05 (-0.81 to 0.71)	0.898
COG_GD_GRP vs EDU_GD_GRP	1	0.49	-0.01 (-0.46 to 0.43)	0.07 (-0.56 to 0.71)	-0.09 (-0.72 to 0.53)	0.17 (-0.72 to 1.06)	0.714

Attention

No inconsistency was observed in the NMA globally (design-by-treatment interaction model: p = 0.667) or locally (node-splitting back-calculation method; see table below).

**Comparison**	**Number of studies**	**Proportion of direct evidence**	**NMA effect SMD (95% CI)**	**Direct evidence effect SMD (95% CI)**	**Indirect evidence effect SMD (95% CI)**	**Difference between direct and indirect effects SMD (95% CI)**	**p-value (back-calculation method)**
COG_GD_GRP vs WL	2	0.86	0.42 (-0.04 to 0.89)	0.48 (-0.03 to 0.98)	0.09 (-1.16 to 1.34)	0.39 (-0.96 to 1.74)	0.571
EDU_GD_GRP vs MBI	2	0.93	0.05 (-0.20 to 0.31)	0.04 (-0.22 to 0.30)	0.30 (-0.68 to 1.27)	-0.26 (-1.27 to 0.75)	0.611
EDU_GD_GRP vs WL	2	0.73	0.24 (-0.10 to 0.58)	0.26 (-0.14 to 0.66)	0.19 (-0.47 to 0.85)	0.07 (-0.70 to 0.84)	0.858
MBI vs WL	2	0.72	0.19 (-0.16 to 0.53)	0.08 (-0.32 to 0.49)	0.46 (-0.20 to 1.12)	-0.38 (-1.15 to 0.39)	0.338
COG_GD_GRP vs EDU_GD_GRP	1	0.62	0.18 (-0.32 to 0.69)	-0.04 (-0.68 to 0.59)	0.56 (-0.26 to 1.38)	-0.60 (-1.64 to 0.44)	0.256
COG_GD_IND vs COG_SELF_IND	1	0.84	0.00 (-0.37 to 0.38)	0.06 (-0.35 to 0.47)	-0.28 (-1.21 to 0.64)	0.35 (-0.67 to 1.36)	0.505
COG_GD_IND vs WL	1	0.87	0.23 (-0.14 to 0.60)	0.18 (-0.21 to 0.58)	0.55 (-0.46 to 1.57)	-0.37 (-1.46 to 0.72)	0.505

Working memory

No inconsistency was observed in the NMA globally (design-by-treatment interaction model: p = 0.932) or locally (node-splitting back-calculation method; see table below).

**Comparison**	**Number of studies**	**Proportion of direct evidence**	**NMA effect SMD (95% CI)**	**Direct evidence effect SMD (95% CI)**	**Indirect evidence effect SMD (95% CI)**	**Difference between direct and indirect effects SMD (95% CI)**	**p-value (back-calculation method)**
COG_GD_GRP vs WL	3	0.93	0.48 (0.05 to 0.92)	0.43 (-0.02 to 0.88)	1.21 (-0.43 to 2.84)	-0.78 (-2.47 to 0.92)	0.368
EDU_GD_GRP vs WL	2	0.88	0.23 (-0.22 to 0.68)	0.13 (-0.35 to 0.61)	0.93 (-0.35 to 2.21)	-0.80 (-2.17 to 0.57)	0.253
MBI vs WL	2	0.92	0.27 (-0.21 to 0.75)	0.33 (-0.16 to 0.83)	-0.47 (-2.16 to 1.21)	0.81 (-0.95 to 2.56)	0.368
COG_GD_GRP vs EDU_GD_GRP	1	0.55	0.26 (-0.29 to 0.80)	0.33 (-0.41 to 1.07)	0.16 (-0.65 to 0.98)	0.17 (-0.93 to 1.27)	0.759
COG_GD_IND vs COG_SELF_IND	1	0.86	0.13 (-0.38 to 0.64)	0.11 (-0.44 to 0.66)	0.27 (-1.09 to 1.62)	-0.16 (-1.62 to 1.30)	0.831
COG_GD_IND vs WL	1	0.87	0.47 (-0.04 to 0.98)	0.49 (-0.05 to 1.04)	0.33 (-1.08 to 1.73)	0.16 (-1.34 to 1.67)	0.831
EDU_GD_GRP vs MBI	1	0.77	-0.04 (-0.57 to 0.49)	0.16 (-0.45 to 0.77)	-0.70 (-1.80 to 0.39)	0.86 (-0.39 to 2.12)	0.177

Certainty of evidence (CINeMA) appraisals

In the tables below, final confidence ratings were assessed using an indirectness value of ‘2’ (Some concerns) as well as ‘1’ (No concerns) as a sensitivity analysis.

Learning 0 weeks post-intervention CINeMA appraisal

Comparison	Number of studies	Within-study bias	Reporting bias	Imprecision	Heterogeneity	Indirectness	Incoherence	Confidence rating (Indirectness = 2)	Confidence rating (Indirectness = 1)
Mixed evidence									
behavioural_guide_individual:support_guide_individual	1	Major concerns	Some concerns	Some concerns	Some concerns	Some concerns	No concerns	Very low	Very low
behavioural_guide_individual:wl	1	Major concerns	Some concerns	Some concerns	No concerns	Some concerns	No concerns	Very low	Very low
cog_guide_group:edu_guide_group	1	Major concerns	Some concerns	Some concerns	No concerns	Some concerns	No concerns	Very low	Very low
cog_guide_group:wl	3	Major concerns	Some concerns	Some concerns	No concerns	Some concerns	No concerns	Very low	Very low
cog_self:wl	1	Major concerns	Some concerns	Some concerns	No concerns	Some concerns	No concerns	Very low	Very low
edu+cog_guide_group:wl	1	Major concerns	Some concerns	No concerns	No concerns	Some concerns	No concerns	Very low	Low
edu_guide_group:mbi	2	Major concerns	Some concerns	No concerns	No concerns	Some concerns	No concerns	Very low	Low
edu_guide_group:wl	2	Major concerns	Some concerns	No concerns	Some concerns	Some concerns	No concerns	Very low	Very low
exercise_guide_group:wl	2	Major concerns	Some concerns	No concerns	No concerns	Some concerns	No concerns	Very low	Low
mbi:music	1	Major concerns	Some concerns	Some concerns	Some concerns	Some concerns	No concerns	Very low	Very low
mbi:wl	2	Major concerns	Some concerns	No concerns	Some concerns	Some concerns	No concerns	Very low	Very low
Indirect evidence									
behavioural_guide_individual:cog_guide_group	0	Major concerns	Some concerns	Major concerns	No concerns	Some concerns	No concerns	Very low	Very low
behavioural_guide_individual:cog_self	0	Major concerns	Some concerns	Major concerns	No concerns	Some concerns	No concerns	Very low	Very low
behavioural_guide_individual:edu+cog_guide_group	0	Major concerns	Some concerns	Some concerns	No concerns	Some concerns	No concerns	Very low	Very low
behavioural_guide_individual:edu_guide_group	0	Major concerns	Some concerns	Some concerns	Some concerns	Some concerns	No concerns	Very low	Very low
behavioural_guide_individual:exercise_guide_group	0	Major concerns	Some concerns	Some concerns	Some concerns	Some concerns	No concerns	Very low	Very low
behavioural_guide_individual:mbi	0	Major concerns	Some concerns	Major concerns	No concerns	Some concerns	No concerns	Very low	Very low
behavioural_guide_individual:music	0	Major concerns	Some concerns	Major concerns	No concerns	Some concerns	No concerns	Very low	Very low
cog_guide_group:cog_self	0	Major concerns	Some concerns	Some concerns	Some concerns	Some concerns	No concerns	Very low	Very low
cog_guide_group:edu+cog_guide_group	0	Major concerns	Some concerns	No concerns	Some concerns	Some concerns	No concerns	Very low	Very low
cog_guide_group:exercise_guide_group	0	Major concerns	Some concerns	Some concerns	No concerns	Some concerns	No concerns	Very low	Very low
cog_guide_group:mbi	0	Major concerns	Some concerns	Some concerns	Some concerns	Some concerns	No concerns	Very low	Very low
cog_guide_group:music	0	Major concerns	Some concerns	Major concerns	No concerns	Some concerns	No concerns	Very low	Very low
cog_guide_group:support_guide_individual	0	Major concerns	Some concerns	Major concerns	No concerns	Some concerns	No concerns	Very low	Very low
cog_self:edu+cog_guide_group	0	Major concerns	Some concerns	No concerns	Some concerns	Some concerns	No concerns	Very low	Very low
cog_self:edu_guide_group	0	Major concerns	Some concerns	Some concerns	Some concerns	Some concerns	No concerns	Very low	Very low
cog_self:exercise_guide_group	0	Major concerns	Some concerns	Some concerns	No concerns	Some concerns	No concerns	Very low	Very low
cog_self:mbi	0	Major concerns	Some concerns	Some concerns	Some concerns	Some concerns	No concerns	Very low	Very low
cog_self:music	0	Major concerns	Some concerns	Major concerns	No concerns	Some concerns	No concerns	Very low	Very low
cog_self:support_guide_individual	0	Major concerns	Some concerns	Some concerns	Some concerns	Some concerns	No concerns	Very low	Very low
edu+cog_guide_group:edu_guide_group	0	Major concerns	Some concerns	No concerns	No concerns	Some concerns	No concerns	Very low	Low
edu+cog_guide_group:exercise_guide_group	0	Major concerns	Some concerns	No concerns	No concerns	Some concerns	No concerns	Very low	Low
edu+cog_guide_group:mbi	0	Major concerns	Some concerns	No concerns	No concerns	Some concerns	No concerns	Very low	Low
edu+cog_guide_group:music	0	Major concerns	Some concerns	No concerns	Some concerns	Some concerns	No concerns	Very low	Very low
edu+cog_guide_group:support_guide_individual	0	Major concerns	Some concerns	Major concerns	No concerns	Some concerns	No concerns	Very low	Very low
edu_guide_group:exercise_guide_group	0	Major concerns	Some concerns	Some concerns	No concerns	Some concerns	No concerns	Very low	Very low
edu_guide_group:music	0	Major concerns	Some concerns	Major concerns	No concerns	Some concerns	No concerns	Very low	Very low
edu_guide_group:support_guide_individual	0	Major concerns	Some concerns	Some concerns	Some concerns	Some concerns	No concerns	Very low	Very low
exercise_guide_group:mbi	0	Major concerns	Some concerns	Some concerns	No concerns	Some concerns	No concerns	Very low	Very low
exercise_guide_group:music	0	Major concerns	Some concerns	Major concerns	No concerns	Some concerns	No concerns	Very low	Very low
exercise_guide_group:support_guide_individual	0	Major concerns	Some concerns	Some concerns	Some concerns	Some concerns	No concerns	Very low	Very low
mbi:support_guide_individual	0	Major concerns	Some concerns	Some concerns	Some concerns	Some concerns	No concerns	Very low	Very low
music:support_guide_individual	0	Major concerns	Some concerns	Some concerns	Some concerns	Some concerns	No concerns	Very low	Very low
music:wl	0	Major concerns	Some concerns	Major concerns	No concerns	Some concerns	No concerns	Very low	Very low
support_guide_individual:wl	0	Major concerns	Some concerns	Some concerns	Some concerns	Some concerns	No concerns	Very low	Very low

Memory 0 weeks post-intervention CINeMA appraisal

Comparison	Number of studies	Within-study bias	Reporting bias	Indirectness	Imprecision	Heterogeneity	Incoherence	Confidence rating (Indirectness = 2)	Confidence rating (Indirectness = 1)
Mixed evidence									
cog_guide_group:edu_guide_group	1	Major concerns	Some concerns	Some concerns	Some concerns	No concerns	No concerns	Very low	Very low
cog_guide_group:wl	3	Major concerns	Some concerns	Some concerns	Some concerns	No concerns	No concerns	Very low	Very low
cog_self:wl	1	Major concerns	Some concerns	Some concerns	No concerns	Some concerns	No concerns	Very low	Very low
edu+cog_guide_group:wl	1	Major concerns	Some concerns	Some concerns	No concerns	No concerns	No concerns	Very low	Low
edu_guide_group:mbi	2	Major concerns	Some concerns	Some concerns	No concerns	No concerns	No concerns	Very low	Low
edu_guide_group:wl	2	Major concerns	Some concerns	Some concerns	No concerns	Some concerns	No concerns	Very low	Very low
exercise_guide_group:wl	2	Major concerns	Some concerns	Some concerns	No concerns	Some concerns	No concerns	Very low	Very low
mbi:music	1	Major concerns	Some concerns	Some concerns	Some concerns	Some concerns	No concerns	Very low	Very low
mbi:wl	2	Major concerns	Some concerns	Some concerns	Some concerns	No concerns	No concerns	Very low	Very low
Indirect evidence									
cog_guide_group:cog_self	0	Major concerns	Some concerns	Some concerns	Some concerns	No concerns	No concerns	Very low	Very low
cog_guide_group:edu+cog_guide_group	0	Major concerns	Some concerns	Some concerns	Some concerns	Some concerns	No concerns	Very low	Very low
cog_guide_group:exercise_guide_group	0	Major concerns	Some concerns	Some concerns	No concerns	Some concerns	No concerns	Very low	Very low
cog_guide_group:mbi	0	Major concerns	Some concerns	Some concerns	Some concerns	Some concerns	No concerns	Very low	Very low
cog_guide_group:music	0	Major concerns	Some concerns	Some concerns	Some concerns	Some concerns	No concerns	Very low	Very low
cog_self:edu+cog_guide_group	0	Major concerns	Some concerns	Some concerns	Some concerns	No concerns	No concerns	Very low	Very low
cog_self:edu_guide_group	0	Major concerns	Some concerns	Some concerns	Some concerns	Some concerns	No concerns	Very low	Very low
cog_self:exercise_guide_group	0	Major concerns	Some concerns	Some concerns	Some concerns	No concerns	No concerns	Very low	Very low
cog_self:mbi	0	Major concerns	Some concerns	Some concerns	Some concerns	No concerns	No concerns	Very low	Very low
cog_self:music	0	Major concerns	Some concerns	Some concerns	Major concerns	No concerns	No concerns	Very low	Very low
edu+cog_guide_group:edu_guide_group	0	Major concerns	Some concerns	Some concerns	Some concerns	No concerns	No concerns	Very low	Very low
edu+cog_guide_group:exercise_guide_group	0	Major concerns	Some concerns	Some concerns	No concerns	No concerns	No concerns	Very low	Low
edu+cog_guide_group:mbi	0	Major concerns	Some concerns	Some concerns	Some concerns	No concerns	No concerns	Very low	Very low
edu+cog_guide_group:music	0	Major concerns	Some concerns	Some concerns	Some concerns	No concerns	No concerns	Very low	Very low
edu_guide_group:exercise_guide_group	0	Major concerns	Some concerns	Some concerns	Some concerns	No concerns	No concerns	Very low	Very low
edu_guide_group:music	0	Major concerns	Some concerns	Some concerns	Some concerns	Some concerns	No concerns	Very low	Very low
exercise_guide_group:mbi	0	Major concerns	Some concerns	Some concerns	Some concerns	No concerns	No concerns	Very low	Very low
exercise_guide_group:music	0	Major concerns	Some concerns	Some concerns	Major concerns	No concerns	No concerns	Very low	Very low
music:wl	0	Major concerns	Some concerns	Some concerns	Major concerns	No concerns	No concerns	Very low	Very low

Processing speed 0 weeks post-intervention CINeMA appraisal

Comparison	Number of studies	Within-study bias	Reporting bias	Indirectness	Imprecision	Heterogeneity	Incoherence	Confidence rating (Indirectness=2)	Confidence rating (Indirectness=1)
Mixed evidence									
behavioural_guide_individual:support_guide_individual	1	Major concerns	Some concerns	Some concerns	Some concerns	No concerns	No concerns	Very low	Very low
behavioural_guide_individual:wl	1	Major concerns	Some concerns	Some concerns	Some concerns	Some concerns	No concerns	Very low	Very low
cog_guide_group:edu_guide_group	1	Major concerns	Some concerns	Some concerns	Some concerns	No concerns	No concerns	Very low	Very low
cog_guide_group:wl	3	Major concerns	Some concerns	Some concerns	No concerns	No concerns	No concerns	Very low	Low
edu+cog_guide_group:wl	1	Major concerns	Some concerns	Some concerns	No concerns	No concerns	No concerns	Very low	Low
edu_guide_group:mbi	2	Major concerns	Some concerns	Some concerns	No concerns	No concerns	No concerns	Very low	Low
edu_guide_group:wl	2	Major concerns	Some concerns	Some concerns	Some concerns	No concerns	No concerns	Very low	Very low
mbi:music	1	Major concerns	Some concerns	Some concerns	Major concerns	No concerns	No concerns	Very low	Very low
mbi:wl	2	Major concerns	Some concerns	Some concerns	No concerns	Some concerns	No concerns	Very low	Very low
Indirect evidence									
behavioural_guide_individual:cog_guide_group	0	Major concerns	Some concerns	Some concerns	Some concerns	Some concerns	No concerns	Very low	Very low
behavioural_guide_individual:edu+cog_guide_group	0	Major concerns	Some concerns	Some concerns	Major concerns	No concerns	No concerns	Very low	Very low
behavioural_guide_individual:edu_guide_group	0	Major concerns	Some concerns	Some concerns	Major concerns	No concerns	No concerns	Very low	Very low
behavioural_guide_individual:mbi	0	Major concerns	Some concerns	Some concerns	Major concerns	No concerns	No concerns	Very low	Very low
behavioural_guide_individual:music	0	Major concerns	Some concerns	Some concerns	Major concerns	No concerns	No concerns	Very low	Very low
cog_guide_group:edu+cog_guide_group	0	Major concerns	Some concerns	Some concerns	No concerns	Some concerns	No concerns	Very low	Very low
cog_guide_group:mbi	0	Major concerns	Some concerns	Some concerns	No concerns	Some concerns	No concerns	Very low	Very low
cog_guide_group:music	0	Major concerns	Some concerns	Some concerns	Some concerns	Some concerns	No concerns	Very low	Very low
cog_guide_group:support_guide_individual	0	Major concerns	Some concerns	Some concerns	Major concerns	No concerns	No concerns	Very low	Very low
edu+cog_guide_group:edu_guide_group	0	Major concerns	Some concerns	Some concerns	Some concerns	No concerns	No concerns	Very low	Very low
edu+cog_guide_group:mbi	0	Major concerns	Some concerns	Some concerns	Some concerns	Some concerns	No concerns	Very low	Very low
edu+cog_guide_group:music	0	Major concerns	Some concerns	Some concerns	Major concerns	No concerns	No concerns	Very low	Very low
edu+cog_guide_group:support_guide_individual	0	Major concerns	Some concerns	Some concerns	Some concerns	Some concerns	No concerns	Very low	Very low
edu_guide_group:music	0	Major concerns	Some concerns	Some concerns	Some concerns	Some concerns	No concerns	Very low	Very low
edu_guide_group:support_guide_individual	0	Major concerns	Some concerns	Some concerns	Major concerns	No concerns	No concerns	Very low	Very low
mbi:support_guide_individual	0	Major concerns	Some concerns	Some concerns	Some concerns	Some concerns	No concerns	Very low	Very low
music:support_guide_individual	0	Major concerns	Some concerns	Some concerns	Major concerns	No concerns	No concerns	Very low	Very low
music:wl	0	Major concerns	Some concerns	Some concerns	Some concerns	Some concerns	No concerns	Very low	Very low
support_guide_individual:wl	0	Major concerns	Some concerns	Some concerns	Major concerns	No concerns	No concerns	Very low	Very low

Word generation 0 weeks post-intervention CINeMA appraisal

Comparison	Number of studies	Within-study bias	Reporting bias	Indirectness	Imprecision	Heterogeneity	Incoherence	Confidence rating (Indirectness=2)	Confidence rating (Indirectness=1)
**Mixed evidence**									
cog_guide_group:edu_guide_group	1	Major concerns	Some concerns	Some concerns	Some concerns	Some concerns	No concerns	Very low	Very low
cog_guide_group:wl	2	Major concerns	Some concerns	Some concerns	Some concerns	No concerns	No concerns	Very low	Very low
cog_guide_individual:cog_self	1	Major concerns	Some concerns	Some concerns	Some concerns	No concerns	No concerns	Very low	Very low
cog_guide_individual:wl	1	Major concerns	Some concerns	Some concerns	Some concerns	No concerns	No concerns	Very low	Very low
cog_self:wl	2	Major concerns	Some concerns	Some concerns	No concerns	No concerns	No concerns	Very low	Low
edu_guide_group:mbi	2	Major concerns	Some concerns	Some concerns	No concerns	No concerns	No concerns	Very low	Low
edu_guide_group:wl	2	Major concerns	Some concerns	Some concerns	No concerns	Some concerns	No concerns	Very low	Very low
exercise_guide_group:wl	1	Major concerns	Some concerns	Some concerns	Some concerns	Some concerns	No concerns	Very low	Very low
mbi:music	1	Major concerns	Some concerns	Some concerns	Major concerns	No concerns	No concerns	Very low	Very low
mbi:wl	2	Major concerns	Some concerns	Some concerns	Some concerns	No concerns	No concerns	Very low	Very low
**Indirect evidence**									
cog_guide_group:cog_guide_individual	0	Major concerns	Some concerns	Some concerns	Some concerns	No concerns	No concerns	Very low	Very low
cog_guide_group:cog_self	0	Major concerns	Some concerns	Some concerns	Some concerns	Some concerns	No concerns	Very low	Very low
cog_guide_group:exercise_guide_group	0	Major concerns	Some concerns	Some concerns	Some concerns	Some concerns	No concerns	Very low	Very low
cog_guide_group:mbi	0	Major concerns	Some concerns	Some concerns	Some concerns	No concerns	No concerns	Very low	Very low
cog_guide_group:music	0	Major concerns	Some concerns	Some concerns	Some concerns	Some concerns	No concerns	Very low	Very low
cog_guide_individual:edu_guide_group	0	Major concerns	Some concerns	Some concerns	Some concerns	No concerns	No concerns	Very low	Very low
cog_guide_individual:exercise_guide_group	0	Major concerns	Some concerns	Some concerns	Major concerns	No concerns	No concerns	Very low	Very low
cog_guide_individual:mbi	0	Major concerns	Some concerns	Some concerns	Some concerns	Some concerns	No concerns	Very low	Very low
cog_guide_individual:music	0	Major concerns	Some concerns	Some concerns	Major concerns	No concerns	No concerns	Very low	Very low
cog_self:edu_guide_group	0	Major concerns	Some concerns	Some concerns	Some concerns	No concerns	No concerns	Very low	Very low
cog_self:exercise_guide_group	0	Major concerns	Some concerns	Some concerns	Some concerns	Some concerns	No concerns	Very low	Very low
cog_self:mbi	0	Major concerns	Some concerns	Some concerns	Some concerns	No concerns	No concerns	Very low	Very low
cog_self:music	0	Major concerns	Some concerns	Some concerns	Some concerns	Some concerns	No concerns	Very low	Very low
edu_guide_group:exercise_guide_group	0	Major concerns	Some concerns	Some concerns	Some concerns	Some concerns	No concerns	Very low	Very low
edu_guide_group:music	0	Major concerns	Some concerns	Some concerns	Some concerns	Some concerns	No concerns	Very low	Very low
exercise_guide_group:mbi	0	Major concerns	Some concerns	Some concerns	Major concerns	No concerns	No concerns	Very low	Very low
exercise_guide_group:music	0	Major concerns	Some concerns	Some concerns	Major concerns	No concerns	No concerns	Very low	Very low
music:wl	0	Major concerns	Some concerns	Some concerns	Some concerns	Some concerns	No concerns	Very low	Very low

Cognitive flexibility 0 weeks post-intervention CINeMA appraisal

Comparison	Number of studies	Within-study bias	Reporting bias	Indirectness	Imprecision	Heterogeneity	Incoherence	Confidence rating (Indirectness=2)	Confidence rating (Indirectness=1)
**Mixed evidence**									
behavioural_guide_individual:wl	1	Major concerns	Some concerns	Some concerns	Major concerns	No concerns	No concerns	Very low	Very low
cog_guide_group:edu_guide_group	1	Major concerns	Some concerns	Some concerns	No concerns	Major concerns	No concerns	Very low	Very low
cog_guide_group:wl	3	Major concerns	Some concerns	Some concerns	No concerns	Some concerns	No concerns	Very low	Very low
cog_guide_individual:cog_self	1	Major concerns	Some concerns	Some concerns	Some concerns	No concerns	No concerns	Very low	Very low
cog_guide_individual:wl	1	Major concerns	Some concerns	Some concerns	Some concerns	No concerns	No concerns	Very low	Very low
cog_self:wl	1	Major concerns	Some concerns	Some concerns	Some concerns	No concerns	No concerns	Very low	Very low
edu+cog_guide_group:wl	1	Major concerns	Some concerns	Some concerns	Some concerns	No concerns	No concerns	Very low	Very low
edu_guide_group:mbi	2	Major concerns	Some concerns	Some concerns	No concerns	No concerns	No concerns	Very low	Low
edu_guide_group:wl	2	Major concerns	Some concerns	Some concerns	No concerns	Some concerns	No concerns	Very low	Very low
exercise_guide_group:wl	1	Major concerns	Some concerns	Some concerns	Some concerns	Some concerns	No concerns	Very low	Very low
mbi:music	1	Major concerns	Some concerns	Some concerns	Some concerns	Some concerns	No concerns	Very low	Very low
mbi:wl	2	Major concerns	Some concerns	Some concerns	Some concerns	No concerns	No concerns	Very low	Very low
**Indirect evidence**									
behavioural_guide_individual:cog_guide_group	0	Major concerns	Some concerns	Some concerns	Major concerns	No concerns	No concerns	Very low	Very low
behavioural_guide_individual:cog_guide_individual	0	Major concerns	Some concerns	Some concerns	Some concerns	Some concerns	No concerns	Very low	Very low
behavioural_guide_individual:cog_self	0	Major concerns	Some concerns	Some concerns	Major concerns	No concerns	No concerns	Very low	Very low
behavioural_guide_individual:edu+cog_guide_group	0	Major concerns	Some concerns	Some concerns	Some concerns	Some concerns	No concerns	Very low	Very low
behavioural_guide_individual:edu_guide_group	0	Major concerns	Some concerns	Some concerns	Major concerns	No concerns	No concerns	Very low	Very low
behavioural_guide_individual:exercise_guide_group	0	Major concerns	Some concerns	Some concerns	Major concerns	No concerns	No concerns	Very low	Very low
behavioural_guide_individual:mbi	0	Major concerns	Some concerns	Some concerns	Major concerns	No concerns	No concerns	Very low	Very low
behavioural_guide_individual:music	0	Major concerns	Some concerns	Some concerns	Major concerns	No concerns	No concerns	Very low	Very low
cog_guide_group:cog_guide_individual	0	Major concerns	Some concerns	Some concerns	Some concerns	Some concerns	No concerns	Very low	Very low
cog_guide_group:cog_self	0	Major concerns	Some concerns	Some concerns	Some concerns	Some concerns	No concerns	Very low	Very low
cog_guide_group:edu+cog_guide_group	0	Major concerns	Some concerns	Some concerns	Some concerns	No concerns	No concerns	Very low	Very low
cog_guide_group:exercise_guide_group	0	Major concerns	Some concerns	Some concerns	Major concerns	No concerns	No concerns	Very low	Very low
cog_guide_group:mbi	0	Major concerns	Some concerns	Some concerns	Some concerns	Some concerns	No concerns	Very low	Very low
cog_guide_group:music	0	Major concerns	Some concerns	Some concerns	Some concerns	Some concerns	No concerns	Very low	Very low
cog_guide_individual:edu+cog_guide_group	0	Major concerns	Some concerns	Some concerns	Some concerns	Some concerns	No concerns	Very low	Very low
cog_guide_individual:edu_guide_group	0	Major concerns	Some concerns	Some concerns	Some concerns	No concerns	No concerns	Very low	Very low
cog_guide_individual:exercise_guide_group	0	Major concerns	Some concerns	Some concerns	Major concerns	No concerns	No concerns	Very low	Very low
cog_guide_individual:mbi	0	Major concerns	Some concerns	Some concerns	Some concerns	Some concerns	No concerns	Very low	Very low
cog_guide_individual:music	0	Major concerns	Some concerns	Some concerns	Major concerns	No concerns	No concerns	Very low	Very low
cog_self:edu+cog_guide_group	0	Major concerns	Some concerns	Some concerns	Some concerns	Some concerns	No concerns	Very low	Very low
cog_self:edu_guide_group	0	Major concerns	Some concerns	Some concerns	Some concerns	Some concerns	No concerns	Very low	Very low
cog_self:exercise_guide_group	0	Major concerns	Some concerns	Some concerns	Major concerns	No concerns	No concerns	Very low	Very low
cog_self:mbi	0	Major concerns	Some concerns	Some concerns	Major concerns	No concerns	No concerns	Very low	Very low
cog_self:music	0	Major concerns	Some concerns	Some concerns	Major concerns	No concerns	No concerns	Very low	Very low
edu+cog_guide_group:edu_guide_group	0	Major concerns	Some concerns	Some concerns	Some concerns	No concerns	No concerns	Very low	Very low
edu+cog_guide_group:exercise_guide_group	0	Major concerns	Some concerns	Some concerns	Major concerns	No concerns	No concerns	Very low	Very low
edu+cog_guide_group:mbi	0	Major concerns	Some concerns	Some concerns	Some concerns	Some concerns	No concerns	Very low	Very low
edu+cog_guide_group:music	0	Major concerns	Some concerns	Some concerns	Major concerns	No concerns	No concerns	Very low	Very low
edu_guide_group:exercise_guide_group	0	Major concerns	Some concerns	Some concerns	Major concerns	No concerns	No concerns	Very low	Very low
edu_guide_group:music	0	Major concerns	Some concerns	Some concerns	Some concerns	Some concerns	No concerns	Very low	Very low
exercise_guide_group:mbi	0	Major concerns	Some concerns	Some concerns	Major concerns	No concerns	No concerns	Very low	Very low
exercise_guide_group:music	0	Major concerns	Some concerns	Some concerns	Major concerns	No concerns	No concerns	Very low	Very low
music:wl	0	Major concerns	Some concerns	Some concerns	Some concerns	Some concerns	No concerns	Very low	Very low

Attention 0 weeks post-intervention CINeMA appraisal

Comparison	Number of studies	Within-study bias	Reporting bias	Indirectness	Imprecision	Heterogeneity	Incoherence	Confidence rating (Indirectness=2)	Confidence rating (Indirectness=1)
Mixed evidence									
cog_guide_group:edu_guide_group	1	Major concerns	Some concerns	Some concerns	Some concerns	Some concerns	No concerns	Very low	Very low
cog_guide_group:wl	2	Major concerns	Some concerns	Some concerns	Some concerns	No concerns	No concerns	Very low	Very low
cog_guide_individual:cog_self	1	Major concerns	Some concerns	Some concerns	No concerns	Major concerns	No concerns	Very low	Very low
cog_guide_individual:wl	1	Major concerns	Some concerns	Some concerns	Some concerns	No concerns	No concerns	Very low	Very low
cog_self:wl	2	Major concerns	Some concerns	Some concerns	No concerns	Some concerns	No concerns	Very low	Very low
edu+cog_guide_group:wl	1	Major concerns	Some concerns	Some concerns	No concerns	No concerns	No concerns	Very low	Low
edu_guide_group:mbi	2	Major concerns	Some concerns	Some concerns	No concerns	No concerns	No concerns	Very low	Low
edu_guide_group:wl	2	Major concerns	Some concerns	Some concerns	Some concerns	No concerns	No concerns	Very low	Very low
mbi:wl	2	Major concerns	Some concerns	Some concerns	Some concerns	No concerns	No concerns	Very low	Very low
Indirect evidence									
cog_guide_group:cog_guide_individual	0	Major concerns	Some concerns	Some concerns	Some concerns	Some concerns	No concerns	Very low	Very low
cog_guide_group:cog_self	0	Major concerns	Some concerns	Some concerns	Some concerns	Some concerns	No concerns	Very low	Very low
cog_guide_group:edu+cog_guide_group	0	Major concerns	Some concerns	Some concerns	Some concerns	No concerns	No concerns	Very low	Very low
cog_guide_group:mbi	0	Major concerns	Some concerns	Some concerns	Some concerns	Some concerns	No concerns	Very low	Very low
cog_guide_individual:edu+cog_guide_group	0	Major concerns	Some concerns	Some concerns	No concerns	Some concerns	No concerns	Very low	Very low
cog_guide_individual:edu_guide_group	0	Major concerns	Some concerns	Some concerns	Some concerns	Some concerns	No concerns	Very low	Very low
cog_guide_individual:mbi	0	Major concerns	Some concerns	Some concerns	Some concerns	Some concerns	No concerns	Very low	Very low
cog_self:edu+cog_guide_group	0	Major concerns	Some concerns	Some concerns	No concerns	Some concerns	No concerns	Very low	Very low
cog_self:edu_guide_group	0	Major concerns	Some concerns	Some concerns	No concerns	Major concerns	No concerns	Very low	Very low
cog_self:mbi	0	Major concerns	Some concerns	Some concerns	No concerns	Major concerns	No concerns	Very low	Very low
edu+cog_guide_group:edu_guide_group	0	Major concerns	Some concerns	Some concerns	No concerns	Some concerns	No concerns	Very low	Very low
edu+cog_guide_group:mbi	0	Major concerns	Some concerns	Some concerns	No concerns	Some concerns	No concerns	Very low	Very low

Working memory 0 weeks post-intervention CINeMA appraisal

Comparison	Number of studies	Within-study bias	Reporting bias	Indirectness	Imprecision	Heterogeneity	Incoherence	Confidence rating (Indirectness=2)	Confidence rating (Indirectness=1)
**Mixed evidence**									
cog_guide_group:edu_guide_group	1	Major concerns	Some concerns	Some concerns	Some concerns	Some concerns	No concerns	Very low	Very low
cog_guide_group:wl	3	Major concerns	Some concerns	Some concerns	No concerns	Some concerns	No concerns	Very low	Very low
cog_guide_individual:cog_self	1	Major concerns	Some concerns	Some concerns	Some concerns	Some concerns	No concerns	Very low	Very low
cog_guide_individual:wl	1	Major concerns	Some concerns	Some concerns	Some concerns	No concerns	No concerns	Very low	Very low
cog_self:wl	2	Major concerns	Some concerns	Some concerns	Some concerns	No concerns	No concerns	Very low	Very low
edu+cog_guide_group:wl	1	Major concerns	Some concerns	Some concerns	No concerns	Some concerns	No concerns	Very low	Very low
edu_guide_group:mbi	1	Major concerns	Some concerns	Some concerns	Some concerns	Some concerns	No concerns	Very low	Very low
edu_guide_group:wl	2	Major concerns	Some concerns	Some concerns	Some concerns	Some concerns	No concerns	Very low	Very low
mbi:wl	2	Major concerns	Some concerns	Some concerns	Some concerns	Some concerns	No concerns	Very low	Very low
**Indirect evidence**									
cog_guide_group:cog_guide_individual	0	Major concerns	Some concerns	Some concerns	Major concerns	No concerns	No concerns	Very low	Very low
cog_guide_group:cog_self	0	Major concerns	Some concerns	Some concerns	Some concerns	Some concerns	No concerns	Very low	Very low
cog_guide_group:edu+cog_guide_group	0	Major concerns	Some concerns	Some concerns	Some concerns	Some concerns	No concerns	Very low	Very low
cog_guide_group:mbi	0	Major concerns	Some concerns	Some concerns	Some concerns	Some concerns	No concerns	Very low	Very low
cog_guide_individual:edu+cog_guide_group	0	Major concerns	Some concerns	Some concerns	Some concerns	Some concerns	No concerns	Very low	Very low
cog_guide_individual:edu_guide_group	0	Major concerns	Some concerns	Some concerns	Some concerns	Some concerns	No concerns	Very low	Very low
cog_guide_individual:mbi	0	Major concerns	Some concerns	Some concerns	Some concerns	Some concerns	No concerns	Very low	Very low
cog_self:edu+cog_guide_group	0	Major concerns	Some concerns	Some concerns	Some concerns	Some concerns	No concerns	Very low	Very low
cog_self:edu_guide_group	0	Major concerns	Some concerns	Some concerns	Some concerns	Some concerns	No concerns	Very low	Very low
cog_self:mbi	0	Major concerns	Some concerns	Some concerns	Major concerns	No concerns	No concerns	Very low	Very low
edu+cog_guide_group:edu_guide_group	0	Major concerns	Some concerns	Some concerns	Some concerns	Some concerns	No concerns	Very low	Very low
edu+cog_guide_group:mbi	0	Major concerns	Some concerns	Some concerns	Some concerns	Some concerns	No concerns	Very low	Very low

File S9: Additional findings

Results from longer-term follow-up NMAs

League tables can be interpreted as described in File S8.

Learning

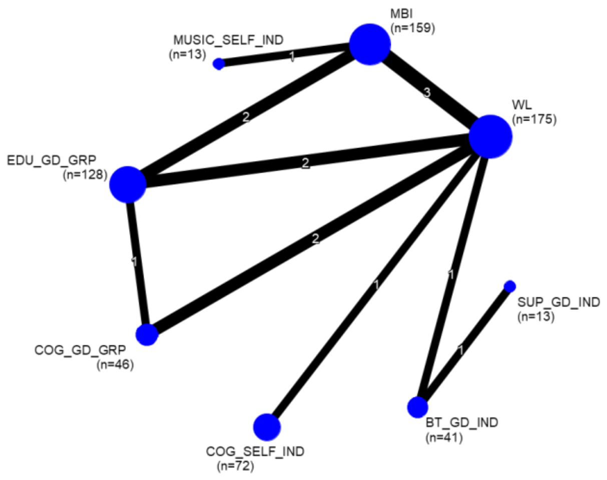

Treatment	SUP_GD_IND	BT_GD_IND	COG_GD_GRP	MBI	COG_SELF_IND	EDU_GD_GRP	MUSIC_SELF_IND	WL
SUP_GD_IND	**0.91**	0.50 (-0.20 to 1.20)	.	.	.	.	.	.
BT_GD_IND	0.50 (-0.20 to 1.20) (-0.41 to 1.41)	**0.60**	.	.	.	.	.	0.40 (-0.23 to 1.02)
COG_GD_GRP	0.57 (-0.46 to 1.60) (-0.79 to 1.92)	0.07 (-0.69 to 0.83) (-0.93 to 1.07)	**0.57**	.	.	-0.10 (-0.74 to 0.55)	.	0.34 (-0.12 to 0.80)
MBI	0.59 (-0.40 to 1.57) (-0.70 to 1.88)	0.09 (-0.61 to 0.79) (-0.83 to 1.00)	0.02 (-0.47 to 0.51) (-0.62 to 0.67)	**0.57**	.	0.03 (-0.23 to 0.29)	0.43 (-0.35 to 1.21)	0.28 (-0.07 to 0.63)
COG_SELF_IND	0.60 (-0.40 to 1.60) (-0.71 to 1.91)	0.10 (-0.62 to 0.82) (-0.84 to 1.04)	0.03 (-0.52 to 0.59) (-0.70 to 0.76)	0.01 (-0.45 to 0.48) (-0.60 to 0.62)	**0.54**	.	.	0.30 (-0.05 to 0.65)
EDU_GD_GRP	0.63 (-0.36 to 1.62) (-0.67 to 1.93)	0.13 (-0.57 to 0.84) (-0.79 to 1.05)	0.06 (-0.42 to 0.54) (-0.57 to 0.69)	0.04 (-0.21 to 0.29) (-0.28 to 0.37)	0.03 (-0.45 to 0.50) (-0.59 to 0.65)	**0.49**	.	0.20 (-0.20 to 0.60)
MUSIC_SELF_IND	1.02 (-0.24 to 2.27) (-0.63 to 2.66)	0.52 (-0.53 to 1.56) (-0.85 to 1.89)	0.45 (-0.47 to 1.37) (-0.76 to 1.65)	0.43 (-0.35 to 1.21) (-0.59 to 1.45)	0.41 (-0.49 to 1.32) (-0.77 to 1.60)	0.39 (-0.43 to 1.20) (-0.68 to 1.46)	**0.18**	.
WL	0.90 (-0.04 to 1.83) (-0.33 to 2.13)	0.40 (-0.23 to 1.02) (-0.42 to 1.22)	0.33 (-0.10 to 0.76) (-0.24 to 0.90)	**0.31 (0.00 to 0.62) (-0.09 to 0.71)**	0.30 (-0.05 to 0.65) (-0.16 to 0.75)	0.27 (-0.05 to 0.59) (-0.15 to 0.69)	-0.12 (-0.95 to 0.72) (-1.21 to 0.98)	**0.13**

Memory

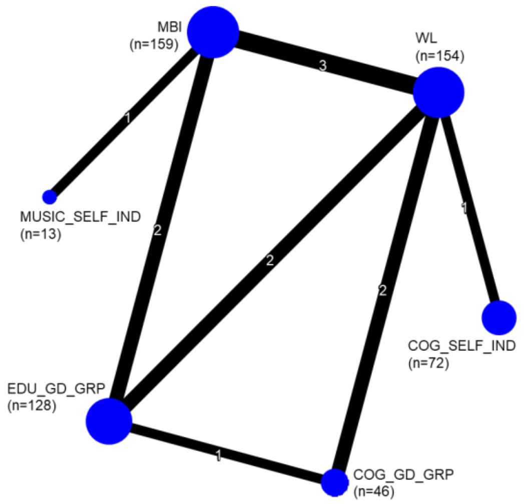

Treatment	COG_GD_GRP	MBI	COG_SELF_IND	EDU_GD_GRP	WL	MUSIC_SELF_IND
COG_GD_GRP	**0.71**	.	.	0.03 (-0.61 to 0.68)	0.24 (-0.22 to 0.70)	.
MBI	0.05 (-0.44 to 0.54) (-0.59 to 0.69)	**0.67**	.	0.05 (-0.21 to 0.31)	0.14 (-0.20 to 0.49)	0.35 (-0.43 to 1.12)
COG_SELF_IND	0.07 (-0.49 to 0.62) (-0.66 to 0.79)	0.02 (-0.45 to 0.48) (-0.59 to 0.62)	**0.62**	.	0.18 (-0.17 to 0.53)	.
EDU_GD_GRP	0.12 (-0.36 to 0.60) (-0.51 to 0.75)	0.07 (-0.18 to 0.32) (-0.26 to 0.40)	0.05 (-0.42 to 0.53) (-0.57 to 0.67)	**0.51**	0.13 (-0.28 to 0.53)	.
WL	0.25 (-0.19 to 0.68) (-0.32 to 0.81)	0.20 (-0.11 to 0.50) (-0.20 to 0.60)	0.18 (-0.17 to 0.53) (-0.28 to 0.64)	0.13 (-0.19 to 0.45) (-0.29 to 0.55)	**0.25**	.
MUSIC_SELF_IND	0.40 (-0.52 to 1.32) (-0.80 to 1.60)	0.35 (-0.43 to 1.12) (-0.67 to 1.37)	0.33 (-0.57 to 1.24) (-0.85 to 1.52)	0.28 (-0.53 to 1.09) (-0.79 to 1.35)	0.15 (-0.68 to 0.99) (-0.94 to 1.24)	**0.25**

Processing speed

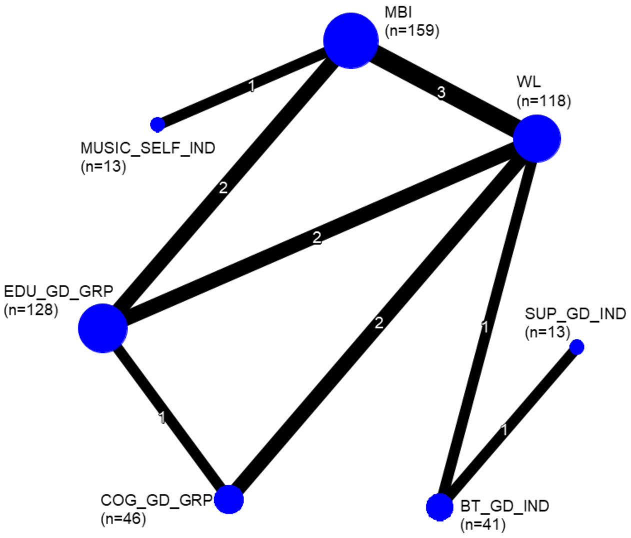

Treatment	MBI	BT_GD_IND	EDU_GD_GRP	MUSIC_SELF_IND	SUP_GD_IND	WL	COG_GD_GRP
**MBI**	**0.81**	.	0.12 (-0.14 to 0.38)	0.22 (-0.55 to 1.00)	.	**0.41 (0.06 to 0.76)**	.
**BT_GD_IND**	0.05 (-0.65 to 0.75) (-0.86 to 0.97)	**0.72**	.	.	0.40 (-0.29 to 1.09)	0.37 (-0.26 to 0.99)	.
**EDU_GD_GRP**	0.11 (-0.14 to 0.36) (-0.22 to 0.43)	0.06 (-0.65 to 0.76) (-0.87 to 0.98)	**0.66**	.	.	0.32 (-0.09 to 0.72)	0.54 (-0.11 to 1.19)
**MUSIC_SELF_IND**	0.22 (-0.55 to 1.00) (-0.79 to 1.24)	0.17 (-0.87 to 1.21) (-1.19 to 1.54)	0.12 (-0.69 to 0.93) (-0.95 to 1.18)	**0.52**	.	.	.
**SUP_GD_IND**	0.45 (-0.53 to 1.43) (-0.84 to 1.74)	0.40 (-0.29 to 1.09) (-0.51 to 1.31)	0.34 (-0.65 to 1.33) (-0.95 to 1.63)	0.22 (-1.02 to 1.47) (-1.41 to 1.86)	**0.33**	.	.
**WL**	**0.42 (0.11 to 0.73) (0.02 to 0.82)**	0.37 (-0.26 to 0.99) (-0.45 to 1.19)	0.31 (-0.01 to 0.63) (-0.11 to 0.73)	0.19 (-0.64 to 1.02) (-0.89 to 1.28)	-0.03 (-0.96 to 0.90) (-1.25 to 1.19)	**0.29**	0.15 (-0.31 to 0.61)
**COG_GD_GRP**	**0.58 (0.08 to 1.07) (-0.07 to 1.22)**	0.52 (-0.24 to 1.28) (-0.47 to 1.52)	0.47 (-0.01 to 0.95) (-0.16 to 1.10)	0.35 (-0.56 to 1.26) (-0.85 to 1.55)	0.13 (-0.90 to 1.15) (-1.22 to 1.47)	0.16 (-0.28 to 0.59) (-0.41 to 0.72)	**0.17**

Word generation

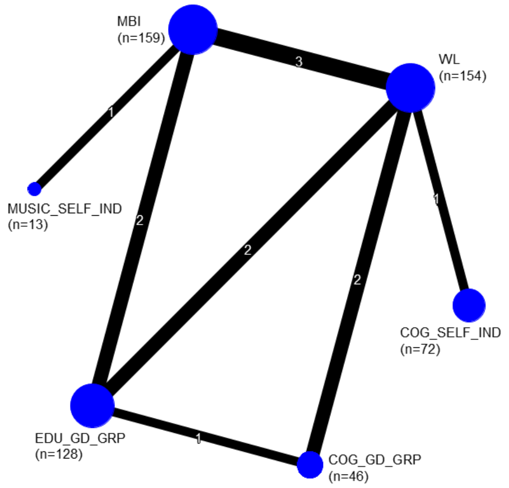

Treatment	COG_SELF_IND	MUSIC_SELF_IND	MBI	WL	EDU_GD_GRP	COG_GD_GRP
COG_SELF_IND	**0.80**	.	.	0.27 (-0.14 to 0.68)	.	.
MUSIC_SELF_IND	-0.07 (-1.03 to 0.89) (-1.35 to 1.22)	**0.77**	0.26 (-0.54 to 1.06)	.	.	.
MBI	0.19 (-0.34 to 0.71) (-0.55 to 0.93)	0.26 (-0.54 to 1.06) (-0.83 to 1.34)	**0.61**	-0.05 (-0.42 to 0.32)	0.21 (-0.09 to 0.52)	.
WL	0.27 (-0.14 to 0.68) (-0.34 to 0.87)	0.34 (-0.53 to 1.20) (-0.83 to 1.50)	0.08 (-0.25 to 0.41) (-0.43 to 0.59)	**0.46**	-0.04 (-0.48 to 0.39)	0.26 (-0.22 to 0.75)
EDU_GD_GRP	0.38 (-0.16 to 0.91) (-0.38 to 1.13)	0.45 (-0.40 to 1.30) (-0.70 to 1.60)	0.19 (-0.10 to 0.48) (-0.28 to 0.66)	0.11 (-0.24 to 0.46) (-0.43 to 0.64)	**0.28**	0.28 (-0.40 to 0.96)
COG_GD_GRP	0.60 (-0.01 to 1.21) (-0.25 to 1.45)	0.67 (-0.28 to 1.63) (-0.61 to 1.96)	0.42 (-0.11 to 0.94) (-0.32 to 1.15)	0.34 (-0.12 to 0.79) (-0.32 to 1.00)	0.23 (-0.28 to 0.73) (-0.49 to 0.95)	**0.09**

Cognitive flexibility

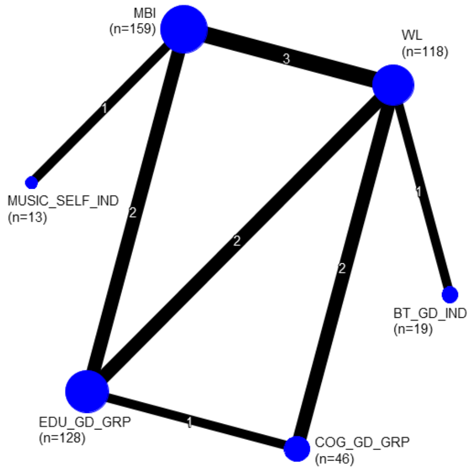

Treatment	MBI	MUSIC_SELF_IND	EDU_GD_GRP	COG_GD_GRP	BT_GD_IND	WL
MBI	**0.75**	-0.11 (-0.88 to 0.66)	0.07 (-0.19 to 0.34)	.	.	0.28 (-0.07 to 0.62)
MUSIC_SELF_IND	-0.11 (-0.88 to 0.66) (-1.12 to 0.90)	**0.73**	.	.	.	.
EDU_GD_GRP	0.10 (-0.15 to 0.35) (-0.23 to 0.42)	0.21 (-0.60 to 1.02) (-0.85 to 1.27)	**0.55**	0.11 (-0.54 to 0.75)	.	0.12 (-0.29 to 0.52)
COG_GD_GRP	0.22 (-0.27 to 0.71) (-0.42 to 0.86)	0.33 (-0.58 to 1.24) (-0.86 to 1.53)	0.12 (-0.35 to 0.60) (-0.50 to 0.75)	**0.38**	.	-0.02 (-0.48 to 0.44)
BT_GD_IND	0.31 (-0.39 to 1.00) (-0.60 to 1.21)	0.42 (-0.62 to 1.45) (-0.94 to 1.77)	0.21 (-0.49 to 0.91) (-0.71 to 1.12)	0.08 (-0.67 to 0.84) (-0.91 to 1.08)	**0.31**	-0.05 (-0.67 to 0.57)
WL	0.26 (-0.05 to 0.56) (-0.14 to 0.66)	0.37 (-0.46 to 1.20) (-0.72 to 1.45)	0.16 (-0.16 to 0.48) (-0.26 to 0.58)	0.04 (-0.40 to 0.47) (-0.53 to 0.60)	-0.05 (-0.67 to 0.57) (-0.86 to 0.76)	**0.28**

Attention

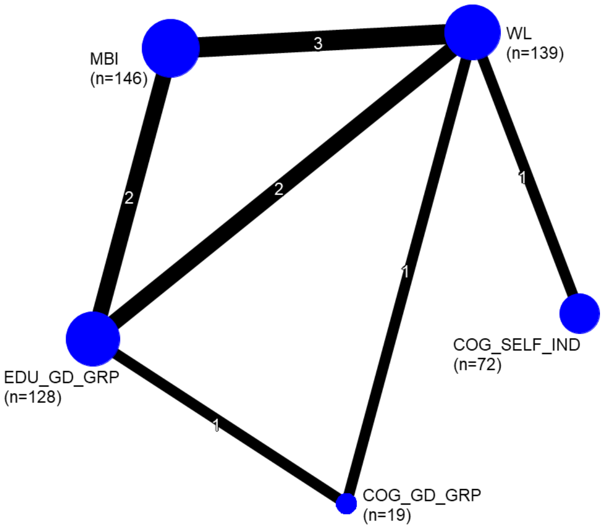

Treatment	COG_SELF_IND	MBI	COG_GD_GRP	EDU_GD_GRP	WL
COG_SELF_IND	**0.80**	.	.	.	**0.49<br>(0.13 to 0.84)**
MBI	0.14<br>(-0.33 to 0.60)<br>(-0.53 to 0.80)	**0.65**	.	0.12<br>(-0.15 to 0.38)	0.31<br>(-0.04 to 0.65)
COG_GD_GRP	0.12<br>(-0.56 to 0.81)<br>(-0.85 to 1.10)	-0.01<br>(-0.62 to 0.59)<br>(-0.87 to 0.85)	**0.61**	0.23<br>(-0.41 to 0.88)	0.26<br>(-0.41 to 0.93)
EDU_GD_GRP	0.27<br>(-0.22 to 0.75)<br>(-0.42 to 0.95)	0.13<br>(-0.12 to 0.38)<br>(-0.22 to 0.48)	0.14<br>(-0.44 to 0.73)<br>(-0.68 to 0.97)	**0.38**	0.33<br>(-0.07 to 0.74)
WL	**0.49<br>(0.13 to 0.84)<br>(-0.01 to 0.99)**	**0.35<br>(0.04 to 0.66)<br>(-0.09 to 0.79)**	0.36<br>(-0.23 to 0.95)<br>(-0.47 to 1.20)	0.22<br>(-0.11 to 0.55)<br>(-0.25 to 0.69)	**0.06**

Working memory

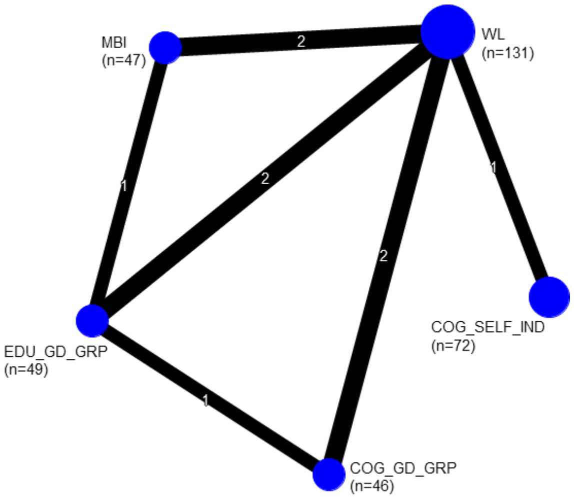

Treatment	COG_SELF_IND	COG_GD_GRP	EDU_GD_GRP	MBI	WL
COG_SELF_IND	**0.78**	.	.	.	**0.39 (0.03 to 0.75)**
COG_GD_GRP	0.03 (-0.54 to 0.60) (-0.91 to 0.96)	**0.74**	0.17 (-0.48 to 0.82)	.	0.28 (-0.18 to 0.75)
EDU_GD_GRP	0.14 (-0.39 to 0.67) (-0.73 to 1.01)	0.11 (-0.39 to 0.62) (-0.72 to 0.94)	**0.59**	0.36 (-0.14 to 0.85)	0.17 (-0.24 to 0.58)
MBI	0.35 (-0.19 to 0.89) (-0.54 to 1.23)	0.32 (-0.25 to 0.89) (-0.61 to 1.25)	0.21 (-0.23 to 0.65) (-0.52 to 0.94)	**0.25**	0.11 (-0.31 to 0.53)
WL	**0.39 (0.03 to 0.75) (-0.21 to 0.99)**	0.36 (-0.08 to 0.81) (-0.37 to 1.09)	0.25 (-0.13 to 0.64) (-0.39 to 0.90)	0.04 (-0.36 to 0.44) (-0.63 to 0.71)	**0.15**

Studies evaluating pharmacological interventions

Two studies from the same research group compared donepezil to placebo[25,26] and reported group-level data adjusted for baseline covariates. In pairwise meta-analyses, very small non-significant effects were found for all outcomes when donepezil was compared to placebo.

Outcome	SMD (95% CI)	I^2	tau^2
**Learning**	0.05 (-0.18 to 0.29)	0.0%	0.000
**Memory**	-0.07 (-0.30 to 0.17)	0.0%	0.000
**Processing speed**	-0.04 (-0.28 to 0.19)	0.0%	0.000
**Word generation**	0.08 (-0.15 to 0.32)	0.0%	0.000
**Cognitive flexibility**	-0.02 (-0.26 to 0.21)	0.0%	0.000
**Working memory**	-0.04 (-0.28 to 0.19)	0.0%	0.000

Additional findings for other non-pharmacological interventions

One study[27] compared exposure to bright white light (n = 20) to exposure to dim red light (n = 22) and reported findings from linear mixed models of group*time interaction effects, incorporating data from three time points (baseline, immediate post-intervention, and 8 weeks post-intervention). Findings from these models are reported in the table below.

Cognitive domain reported	F-statistic (numerator *df*, denominator *df*)	Partial eta^2	p-value
**Verbal memory**[a]	0.80 (2, 77)	0.02	0.43
**Visual memory**[b]	3,77 (2, 77)	0.09	0.03
**Processing speed**[c]	1.51 (2, 77)	0.04	0.23
**Verbal fluency**[d]	1.28 (2, 77)	0.03	0.28
**Executive functioning**[e]	2.42 (2, 77)	0.06	0.10
**Working memory**[f]	1.63 (2, 77)	0.04	0.20

[a] Combined HVLT-R total recall/delayed recall

[b] Combined BVMT total recall/delayed recall

[c] Combined TMT Part A, WAIS-IV Coding, D-KEFS Color-Word Inhibition (Color Name and Word Reading Time)

[d] Combined D-KEFS Verbal Fluency and Animals raw scores

[e] Combined Trail-Making Test B time (reverse scored) + D-KEFS Color-Word Inhibition

[f] WAIS-IV Digit Span: total raw score (sum of forward, backward, and sequencing scores)

Supplement Reference List

1. Spineli LM, Papadimitropoulou K, Kalyvas C. Exploring the Transitivity Assumption in Network Meta-Analysis: A Novel Approach and Its Implications. *Stat Med*. Mar 30 2025;44(7):e70068. doi:10.1002/sim.70068.
2. Hamel C, Hersi M, Kelly SE, et al. Guidance for using artificial intelligence for title and abstract screening while conducting knowledge syntheses. *BMC Med Res Methodol*. Dec 20 2021;21(1):285. doi:10.1186/s12874-021-01451-2.
3. Campbell M, Fitzpatrick R, Haines A, et al. Framework for design and evaluation of complex interventions to improve health. *BMJ*. Sep 16 2000;321(7262):694-6. doi:10.1136/bmj.321.7262.694.
4. Lokker C, McKibbon KA, Colquhoun H, Hempel S. A scoping review of classification schemes of interventions to promote and integrate evidence into practice in healthcare. *Implement Sci*. Mar 3 2015;10:27. doi:10.1186/s13012-015-0220-6.
5. Michie S, Johnston M, Abraham C, et al. Making psychological theory useful for implementing evidence based practice: a consensus approach. *Qual Saf Health Care*. Feb 2005;14(1):26-33. doi:10.1136/qshc.2004.011155.
6. Michie S, van Stralen MM, West R. The behaviour change wheel: a new method for characterising and designing behaviour change interventions. *Implement Sci*. Apr 23 2011;6:42. doi:10.1186/1748-5908-6-42.
7. Van Stan JH, Dijkers MP, Whyte J, et al. The Rehabilitation Treatment Specification System: Implications for Improvements in Research Design, Reporting, Replication, and Synthesis. *Arch Phys Med Rehabil*. Jan 2019;100(1):146-155. doi:10.1016/j.apmr.2018.09.112.
8. Lopez-Lopez JA, Page MJ, Lipsey MW, Higgins JPT. Dealing with effect size multiplicity in systematic reviews and meta-analyses. *Res Synth Methods*. Jul 3 2018;9(3):336-351. doi:10.1002/jrsm.1310.
9. Borenstein M, Hedges LV, Higgins JPT, Rothstein HR. Chapter 24: Multiple Outcomes or Time-Points within a Study. *Introduction to Meta-Analysis*. John Wiley and Sons, Ltd.; 2009:225-238:chap 24.
10. Harrer M, Cuijpers P, Furukawa TA, Ebert D. *Doing Meta-Analysis with R: A Hands-On Guide*. CRC Press; 2022.
11. Grant I, Gonzalez R, Carey CL, Natarajan L, Wolfson T. Non-acute (residual) neurocognitive effects of cannabis use: a meta-analytic study. *J Int Neuropsychol Soc*. Jul 2003;9(5):679-89. doi:10.1017/S1355617703950016.
12. Canada's Drug Agency. Grey Matters: A Tool for Searching Health-related Grey Literature. Accessed 18 March 2025, https://greymatters.cda-amc.ca.
13. Nikolakopoulou A, Higgins JPT, Papakonstantinou T, et al. CINeMA: An approach for assessing confidence in the results of a network meta-analysis. *PLoS Med*. Apr 2020;17(4):e1003082. doi:10.1371/journal.pmed.1003082.
14. Papakonstantinou T, Nikolakopoulou A, Higgins JPT, Egger M, Salanti G. CINeMA: Software for semiautomated assessment of the confidence in the results of network meta-analysis. *Campbell Syst Rev*. Mar 2020;16(1):e1080. doi:10.1002/cl2.1080.
15. Cherrier MM, Higano CS, Gray HJ. Cognitive skill training improves memory, function, and use of cognitive strategies in cancer survivors. *Support Care Cancer*. Jan 2022;30(1):711-720. doi:10.1007/s00520-021-06453-w.
16. Campbell KL, Kam JWY, Neil-Sztramko SE, et al. Effect of aerobic exercise on cancer-associated cognitive impairment: A proof-of-concept RCT. *Psychooncology*. Jan 2018;27(1):53-60. doi:10.1002/pon.4370.

17. Henneghan AM, Becker H, Harrison ML, et al. A randomized control trial of meditation compared to music listening to improve cognitive function for breast cancer survivors: Feasibility and acceptability. *Complement Ther Clin Pract*. Nov 2020;41:101228. doi:10.1016/j.ctcp.2020.101228.
18. Van der Gucht K, Ahmadoun S, Melis M, et al. Effects of a mindfulness-based intervention on cancer-related cognitive impairment: Results of a randomized controlled functional magnetic resonance imaging pilot study. *Cancer*. Sep 15 2020;126(18):4246-4255. doi:10.1002/cncr.33074.
19. Lengacher CA, Reich RR, Rodriguez CS, et al. Efficacy of Mindfulness-Based Stress Reduction for Breast Cancer (MBSR(BC)) a Treatment for Cancer-related Cognitive Impairment (CRCI): A Randomized Controlled Trial. *J Integr Complement Med*. Sep 18 2024. doi:10.1089/jicm.2024.0184.
20. Dos Santos M, Hardy-Leger I, Rigal O, et al. Cognitive rehabilitation program to improve cognition of cancer patients treated with chemotherapy: A 3-arm randomized trial. *Cancer*. Dec 15 2020;126(24):5328-5336. doi:10.1002/cncr.33186.
21. da Costa BR, Nuesch E, Rutjes AW, et al. Combining follow-up and change data is valid in meta-analyses of continuous outcomes: a meta-epidemiological study. *J Clin Epidemiol*. Aug 2013;66(8):847-55. doi:10.1016/j.jclinepi.2013.03.009.
22. Ostinelli EG, Efthimiou O, Luo Y, et al. Combining endpoint and change data did not affect the summary standardised mean difference in pairwise and network meta-analyses: An empirical study in depression. *Res Synth Methods*. Sep 2024;15(5):758-768. doi:10.1002/jrsm.1719.
23. Vickers AJ, Altman DG. Statistics notes: Analysing controlled trials with baseline and follow up measurements. *BMJ*. Nov 10 2001;323(7321):1123-4. doi:10.1136/bmj.323.7321.1123.
24. Higgins JPT, Thomas J, Chandler J, et al. Cochrane Handbook for Systematic Reviews of Interventions Version 6.3 (updated February 2022). GEN. Cochrane. Updated February 2022. Accessed 27 April 2023, 2022. www.training.cochrane.org/handbook.
25. Lawrence JA, Griffin L, Balcueva EP, et al. A study of donepezil in female breast cancer survivors with self-reported cognitive dysfunction 1 to 5 years following adjuvant chemotherapy. *J Cancer Surviv*. Feb 2016;10(1):176-84. doi:10.1007/s11764-015-0463-x.
26. Rapp SR, Dressler EV, Brown WM, et al. Phase III Randomized, Placebo-Controlled Clinical Trial of Donepezil for Treatment of Cognitive Impairment in Breast Cancer Survivors After Adjuvant Chemotherapy (WF-97116). *J Clin Oncol*. Jul 20 2024;42(21):2546-2557. doi:10.1200/JCO.23.01100.
27. Wu LM, Valdimarsdottir HB, Amidi A, et al. Examining the Efficacy of Bright Light Therapy on Cognitive Function in Hematopoietic Stem Cell Transplant Survivors. *J Biol Rhythms*. Oct 2022;37(5):471-483. doi:10.1177/07487304221107833.
